# Improved Latin hypercube sampling initialization-based whale optimization algorithm for COVID-19 X-ray multi-threshold image segmentation

**DOI:** 10.1038/s41598-024-63739-9

**Published:** 2024-06-09

**Authors:** Zhen Wang, Dong Zhao, Ali Asghar Heidari, Yi Chen, Huiling Chen, Guoxi Liang

**Affiliations:** 1 College of Computer Science and Technology, Changchun Normal University, Changchun, 130032 Jilin China; 2 School of Surveying and Geospatial Engineering, College of Engineering, University of Tehran, Tehran, Iran; 3 Key Laboratory of Intelligent Informatics for Safety & Emergency of Zhejiang Province, Wenzhou University, Wenzhou, 325035 China; 4 Department of Artificial Intelligence, Wenzhou Polytechnic, Wenzhou, 325035 China

**Keywords:** COVID-19 X-ray, Multi-threshold image segmentation, Swarm intelligence, Whale optimization algorithm, Computational science, Computer science

## Abstract

Image segmentation techniques play a vital role in aiding COVID-19 diagnosis. Multi-threshold image segmentation methods are favored for their computational simplicity and operational efficiency. Existing threshold selection techniques in multi-threshold image segmentation, such as Kapur based on exhaustive enumeration, often hamper efficiency and accuracy. The whale optimization algorithm (WOA) has shown promise in addressing this challenge, but issues persist, including poor stability, low efficiency, and accuracy in COVID-19 threshold image segmentation. To tackle these issues, we introduce a Latin hypercube sampling initialization-based multi-strategy enhanced WOA (CAGWOA). It incorporates a COS sampling initialization strategy (COSI), an adaptive global search approach (GS), and an all-dimensional neighborhood mechanism (ADN). COSI leverages probability density functions created from Latin hypercube sampling, ensuring even solution space coverage to improve the stability of the segmentation model. GS widens the exploration scope to combat stagnation during iterations and improve segmentation efficiency. ADN refines convergence accuracy around optimal individuals to improve segmentation accuracy. CAGWOA's performance is validated through experiments on various benchmark function test sets. Furthermore, we apply CAGWOA alongside similar methods in a multi-threshold image segmentation model for comparative experiments on lung X-ray images of infected patients. The results demonstrate CAGWOA's superiority, including better image detail preservation, clear segmentation boundaries, and adaptability across different threshold levels.

## Introduction

Image segmentation is a critical stage in medical image processing and analysis that involves dividing pathological images into regions with distinct properties to preserve as much detail of the lesion as possible^1–3^. Studies have shown that image segmentation has significant implications for improving COVID-19’s diagnostic accuracy^4^, assisting physicians in developing treatment plans^5^, and improving diagnostic speed^6^ and medical efficiency^7^. In the second chapter of this paper, the related work section, a brief summary of the advantages and drawbacks present in the existing image segmentation methods is presented.

Among various image segmentation methods, the multi-threshold image segmentation (MTIS) technique leverages the greyscale features of images. This method offers the advantages of simple computation and high operational efficiency. And the processing of thresholds significantly affects the performance of MTIS methods. Among them, Kapur's entropy method is a notable work. Kapur's entropy can effectively differentiate different organizations and structures in an image by maximizing the entropy of image information between segmented regions and dividing the image into regions with different features. However, Kapur's entropy calculation necessitates enumeration to determine the optimal threshold value, resulting in exponential growth in computational complexity as the number of thresholds and the search space expand^8–10^.

An effective way to deal with this is to combine the swarm intelligent optimization method^11,12^. Section "Related works" of this paper shows several real-life examples of swarm intelligent optimization algorithms combined with multi-threshold image segmentation methods. Numerous research results have shown that incorporating swarm intelligence optimization algorithms can improve the accuracy, speed, robustness, and self-adaptability of MTIS methods. In addition, the performance of the swarm intelligence optimization algorithm can significantly affect the efficiency and results of image segmentation^13^, so further optimization of the algorithm is needed to adapt and solve the optimization problems on different segmentation tasks when the algorithm is applied to the field of COVID-19 medical image segmentation.

Swarm intelligence optimization algorithms are a class of computational methods based on the intelligent behavior of populations in nature, mainly by stimulating the collaborative and adaptive behavior of biological or social groups in problem-solving to achieve optimization of goals. It has the features of a simple structure, fast convergence, and good global convergence. It is mostly applied to solve global search and large-scale multi-objective optimization problems. Since its introduction, researchers have proposed a series of optimization algorithms. Such as differential evolution algorithm (DE)^14^, ant colony optimization algorithm (ACO)^15^, wind driven optimization algorithm (WDO)^16^, moth-flame optimization algorithm (MFO)^17^, the Sine Cosine algorithm (SCA)^18^, colony predation algorithm (CPA)^19^, bat optimization algorithm (BA)^20^, hunger games search algorithm (HGS)^21^, Harris hawks optimization algorithm (HHO)^22^, particle swarm optimization algorithm (PSO)^23^, firefly optimization algorithm (FA)^24^, grey wolf optimization algorithm (GWO)^25^, and Runge Kutta optimizer (RUN)^26^ and whale optimization algorithm (WOA)^27^.

Among the swarm intelligence optimization algorithms, WOA simulates the hunting behavior of humpback whale populations. The algorithm performs a parallel search through multiple candidate solutions and combines exploration and exploitation strategies during the exploration process to gradually approach the optimal solution. It is characterized by low parameter requirements, high adaptability, and global exploration capability. Based on the advantages of the WOA, researchers have proposed many WOA variants to solve optimization problems in various domains. For example, an improved WOA was applied to army planning and strategy alignment^28^. To solve the global search problem, an improved WOA has been proposed by Chakraborty et al.^29^. Zhang et al. proposed an enhanced WOA for solving the traveler's problem^30^. Huang et al. introduced an improved WOA and used it for structural damage identification^31^. An estimation method for short-term natural gas usage based on the Volterra adaptive filter and enhanced WOA^32^. Pandey et al. use enhanced whale optimization for posture detection^33^. Chen et al. introduced a WOA based on dual adaptive and stochastic substitution^34^. Jia et al. introduced a cloud computing task scheduling model based on the improved WOA^35^. A large number of research results have shown that the improved whale optimization algorithm has good performance in dealing with optimization problems and finding the optimal values in the solution space. Therefore, WOA can be tried to solve optimization problems within the field of COVID-19 multi-threshold image segmentation.

Although numerous variants of the WOA have been proposed, they still experience slow convergence speed and low convergence accuracy when addressing complex high-dimensional problems. There is still a lot of room for improvement in the exploitation capability and algorithm adaptability of the WOA. And because of the theorem that there is no free lunch and Ref.^36^, no optimization algorithm can solve all optimization problems in all domains, it is necessary to further improve the WOA when it is applied to image segment-rays of X-rays of the lungs of patients with novel coronary pneumonia.

To further improve the segmentation efficiency and diagnosis of lung images in patients with novel coronary pneumonia, a new swarm intelligence optimization algorithm, CAGWOA, is proposed in this paper. CAGWOA introduces an adaptive global search strategy based on the WOA, using the optimal individual as a guide and introducing the random interindividual distance as a step size to increase the range of individual activities while improving the convergence speed and segmentation efficiency. Through the all-dimensional neighborhood mechanism, the backup population is exploited around the space near the optimal individual, which improves the convergence accuracy of the algorithm and the accuracy of segmentation results.

Furthermore, numerous swarm intelligence optimization algorithms encounter issues such as getting trapped in local optima and exhibiting slow convergence when addressing high-dimensional complex problems. This is largely caused by the uncertainty and instability of the algorithm's initialization method^37,38^. The traditional initialization method based on random numbers is memoryless. Its uncertain initial state distribution cannot effectively sample the features of the problem space. In particular, with a limited number of individuals, random initialization can have a significant negative impact on multimodal and mixed function optimization problems and complex optimization problems in threshold image segmentation^39–41^. In contrast, the Latin hypercubic sampling (LHS) method^42,43^, inspired by 2D Latin sampling, aims to recreate probability distributions with fewer samples by stratifying them. LHS ensures a uniform distribution of samples in each dimension, reduces the correlation between samples, and can provide a favorable representation of the solution space in a relatively small number of sample points by efficiently utilizing the samples. Linking this to swarm intelligence optimization, the initialization using LHS can improve the exploration of the solution space through a more structured and informed approach^44^. Therefore, in this paper, we design and introduce a COS sampling initialization method based on LHS to achieve uniform coverage of the sampling space with the same number of individuals to improve the stability of the segmentation model.

To validate the performance of CAGWOA, this paper conducted a series of comparison experiments on IEEE CEC 2014 benchmark functions^45^, which contained ablation experiments, comparisons with some excellent peers, and WOA variants. In addition to IEEE CEC 2014, comparison experiments with other improved algorithms were conducted on the more complex and challenging IEEE CEC 2019^46^ and IEEE CEC 2022^47^. The results demonstrate that the CAGWOA exhibits strong optimization performance. By integrating 2D Kapur’s entropy, nonlocal means, and 2D distribution histograms, this paper proposes a multi-threshold image segmentation model based on CAGWOA. To verify the model’s effectiveness in segmenting lung images of COVID-19 patients, it is compared with several similar methods in image segmentation experiments.

The contributions of this paper are categorized as follows:

- A new COS sampling initialization strategy is proposed to achieve uniform sampling of the solution space with the same number of individuals.
- An enhanced whale optimization algorithm (CAGWOA) for image segmentation is proposed.
- The performance of CAGWOA is verified by comparing it with some excellent algorithms.
- CAGWOA is applied to multi-level thresholding for COVID-19 X-ray image segmentation.

The rest of the paper is organized as follows: In Sect. "Related works", the relevant segmentation methods for image segmentation are briefly introduced according to different segmentation methods. Section "Theoretical backgrounds" presents a 2D Kapur’s entropy image segmentation model based on CAGWOA, nonlocal means, and a 2D distribution histogram. And the background of the WOA, main update phases, and specific update formulas. The flowchart and pseudo-code for CAGWOA are provided in Sect. "The proposed CAGWOA", along with a description of the algorithm's structure and optimization strategy. In Sect. "Benchmark experimental results and discussion", the performance of CAGWOA was tested on benchmark functions. Section "Segmentation experiments for COVID-19 X-ray image" tests the performance of the image segmentation model based on the CAGWOA. The conclusions and future work of this paper are presented in Sect. "Conclusion and future works". Some relevant experimental results are shown in the appendix.

## Related works

As a fundamental task in medical image processing, image segmentation aims to identify and understand the content of an image by dividing a digital image into multiple sets of pixels with the same characteristics. Image segmentation methods mainly contain the following five categories: deep learning-based image segmentation methods^48^, clustering segmentation methods^49^, histogram segmentation methods^50^, edge detection methods^51^, and thresholding segmentation methods^52^.

In recent years, deep learning-based image segmentation methods have garnered significant attention^53–55^. For instance, a convolution-based modified adaptive k-means method has been proposed^56^. Wang et al. introduced a novel deep learning-based interactive segmentation framework for 2D segmentation of multiple organs in fetal MRI sections and 3D segmentation of brain tumor cores^57^. Işın et al. explored deep learning techniques for brain tumor image segmentation and diagnosis^58^. Isensee et al. developed nnU-Net, a deep learning-based segmentation method for medical image segmentation applications^59^. Haque et al. reviewed the fundamentals of deep learning methods and their implementation in various medical applications^60^. Furthermore, several approaches have been developed for COVID-19 diagnosis using medical imaging: IP-based SCA evolved deep convolutional neural networks for chest CT scans^61^, improved deep convolutional neural networks using the chimp optimization algorithm for X-ray images^62^, automatic COVID-19 diagnosis from chest X-ray images using a deep trigonometric convolutional neural network^63^, and real-time COVID-19 diagnosis from X-ray images using deep CNN and extreme learning machines stabilized by the chimp optimization algorithm^64^. However, deep learning-based image segmentation methods tend to have disadvantages such as high time complexity, inability to perform real-time segmentation, coarse utilization of global contextual information, and unfavorable application to 3D image segmentation^65,66^.

A clustering segmentation method based on feature similarity division, such as a clustering-based approach using a hierarchical evolutionary algorithm, has been proposed for medical image segmentation. This method can automatically classify the image into appropriate classes, thereby avoiding the difficulty of determining the proper number of classes^67^. Another new defect segmentation method leverages color features and a K-means clustering unsupervised algorithm^68^. A K-means-based clustering technique has also been used for image segmentation in different color spaces^69^. Additionally, Juang et al. proposed a tumor object tracking method for MRI brain images using a color-transformed segmentation algorithm and K-means clustering technique^70^. Clustering methods can segment images into appropriately sized and compact blocks of pixels. However, it is easy to produce the wrong segmentation for objects with complex structures in the image^71,72^.

Segmentation methods are based on histograms of image gray levels. An example is an image segmentation method combining weighted histogram equalization with adaptive gamma correction for homomorphic filtering^73^. An improved FCM algorithm based on a given image histogram^74^. Bonnet et al. proposed to obtain segmented images by linking membership classes with each pixel point to deblur the relaxed membership classes^75^. To solve the problem of quantitative reduction of image data from histograms, a novel automatic peak detection algorithm was introduced by Sezan et al.^76^. Ni et al. proposed and analyzed a region-based, nonparametric active contour model based on histograms for the segmentation of cluttered scenes^77^. The segmentation method based on the histogram of the image gray level is suitable for images with high contrast and less complexity, but is susceptible to noise interference, resulting in unsatisfactory segmentation results^78,79^.

Edge detection method based on luminance and continuity segmentation. For example, Boskovitz et al. introduced an automatically adaptive neuro-fuzzy segmentation and edge detection architecture^80^. Savant proposed an improved method for range image segmentation on the basis of edge detection techniques^81^. Bellon et al. proposed a region-based discontinuous edge detection segmentation method^82^. Meftah et al. introduced a spiking neural network applied to image segmentation and edge detection^83^. Singleton et al. developed a highly sensitive edge detector using an organizational classification of pixels based on their local neighborhood data analysis^84^. Image segmentation methods based on edge detection often fail to achieve good segmentation results when the differences in the size of gray values in edge regions are small. And in the segmentation process, it is easy to be interfered with by noise or other information^85,86^.

Segmentation methods based on thresholding of foreground and background, like a technique for picture segmentation that employs a modified edge tracking methodology^87^, and a new segmentation method with local thresholding applied to $\mu C$ analysis of skeletal samples^88^. An MTIS technique based on an enhanced ant colony optimization algorithm was proposed by Zhao et al.^89^. Al-amri et al. investigated image segmentation techniques using multiple thresholding methods^90^. Abdel-Basset et al. used an enhanced heuristic algorithm for optimal threshold finding in thresholding image segmentation^91^. The threshold-based segmentation method directly utilizes the grayscale characteristics of the image, which has the advantages of simple computation and high operational efficiency. However, it is sensitive to noise and cannot obtain accurate segmentation results for images with insignificant and overlapping grayscale differences. Moreover, the threshold value obtained by using only the gray value distribution only reflects the magnitude of the pixel gray level and does not reflect the spatial relationship between the pixel and the domain, which easily causes problems such as segmentation errors and a low signal-to-noise ratio. Table 1 gives a brief description of the advantages and drawbacks of the above-mentioned image segmentation methods.

**Table 1:** Summaries of image segmentation techniques.

Image segmentation techniques		
Methods	Advantages	Disadvantages
Deep learning-based	Ability to handle complex image segmentation tasks Has strong generalization ability	High time complexity Inability to perform real-time segmentation Coarse utilization of global contextual information
Clustering-based	Relatively high accuracy and adaptability	Difficult to deal with objects with complex structures in the image
Histogram-based	Can efficiently process images with higher contrast and lower complexity	Susceptible to be interference by noise Unsatisfactory segmentation results
Edge detection-based	Effective retention of local features in images	Sensitivity to differences in grey values in edge regions Easy to be interfered by noise or other information
Thresholding-based	Simple calculation and high operational efficiency	Sensitive to image noise, grey scale differences and image overlapping Cannot reflect the spatial relationship

To solve the above-mentioned problems in image segmentation techniques, this paper uses a 2D distribution histogram^92^ combining grayscale values and nonlocal mean values for thresholding to improve the accuracy and noise immunity of segmentation^93^. Additionally, entropy measures the disorder or randomness in a system. In an image, uniform regions correspond to minimal entropy, while non-uniform regions exhibit maximal entropy. Therefore, high entropy in a segmented image indicates better separation between the target and background regions. Based on this concept, Shannon entropy, Rényi entropy, Tsallis entropy, Cross entropy, and Kapur entropy have been proposed as popular entropy calculations for threshold image segmentation. Among them, the Kapur maximum entropy method^94^ maximizes the information entropy of the image by seeking the globally optimal segmentation threshold to make the segmentation result more informative. And the method does not need to make prior assumptions about the characteristics of the image or pre-set the number of segmentation thresholds. This makes it more universal and can be applied to various types of images without relying on specific a priori knowledge. However, the computation of the traditional Kapur entropy requires traversing all possible segmentation thresholds and selecting the set of thresholds that maximize the Kapur entropy as the final set of segmentation thresholds, whose complexity rises exponentially with the increase of the thresholds and the size of the image.

Numerous studies have demonstrated that optimization-based algorithms for selecting the optimal set of thresholds are highly effective. However, the performance of these algorithms, particularly their global search capability and ability to avoid local optima, significantly impacts the efficiency and outcomes of image segmentation. This paper introduces an improved WOA, called CAGWOA, designed to find the optimal threshold vector for Kapur’s entropy. The 2D Kapur’s entropy serves as the objective function for CAGWOA, providing an optimal threshold set for the image segmentation model.

## Theoretical backgrounds

In this section, a Kapur’s entropy MTIS model based on a 2D histogram is illustrated. In addition, the algorithmic structure and theoretical foundation of the whale optimization algorithm are briefly described.

### MTIS method

In this paper, we construct an image segmentation model that aims to solve the problem of noise sensitivity in MTIS. First, the original image is grayscale processed to generate a grayscale image. Then, a nonlocal mean filtering process is applied to the grayscale image to obtain the filtered image. In order to avoid segmentation errors due to inconspicuous grayscale differences and overlapping regions, the nonlocal mean filtered image and the grayscale image are combined to form a 2D distribution histogram. Then, the 2D distribution histogram is thresholded using Kapur's entropy to generate the set of entropy values. Finally, CAGWOA is used as the objective function to find the best threshold vector in Kapur’s entropy threshold set.

#### Nonlocal means and 2D histogram

As an effective method of removing noise from an image, nonlocal mean filtering is achieved by taking all the pixels in an image, in pixels or blocks of pixels, and applying a weighted average based on similarity. The nonlocal mean filtering technique treats the image with high definition and without loss of details.

If $I(q)$ and $I(p)$ are used to denote the grayscale values of two pixels $q$ and $p$ in image $I$ produced by image grayscale processing, Eq. (1) can be used to determine the pixel point $p$ 's nonlocal mean value. It can be indicated by the notation $O(p)$. The weight between two pixels, $p$ and $q$, is represented by $\omega (p,q)$ in Eq. (2), and $\sigma$ is the standard deviation. In Eq. (3), $L(p)$ denotes a block of pixels of size $m*m$ centered at pixel point $p$. $L(q)$ denotes a block of pixels centered at pixel point $q$ in Eq. (4). $\mu (p)$ and $\mu (q)$ indicate the local means of two pixel points $q$ and $p$.

1 $$O\left(p\right)=\frac{\sum_{q\in I} I\left(q\right)\omega \left(p,q\right)}{\sum_{q\in I} \omega \left(p,q\right)},$$

2 $$\omega (p,q)={\text{exp}}^{-\frac{|\mu (p)-\mu (q){|}^{2}}{{\sigma }^{2}},}$$

3 $$\mu \left(p\right)=\frac{1}{m\times m}\sum_{i\in L\left(p\right)} I\left(i\right),$$

4 $$\mu \left(q\right)=\frac{1}{m\times m}\sum_{i\in L\left(q\right)} I\left(i\right).$$

**Figure 1:**
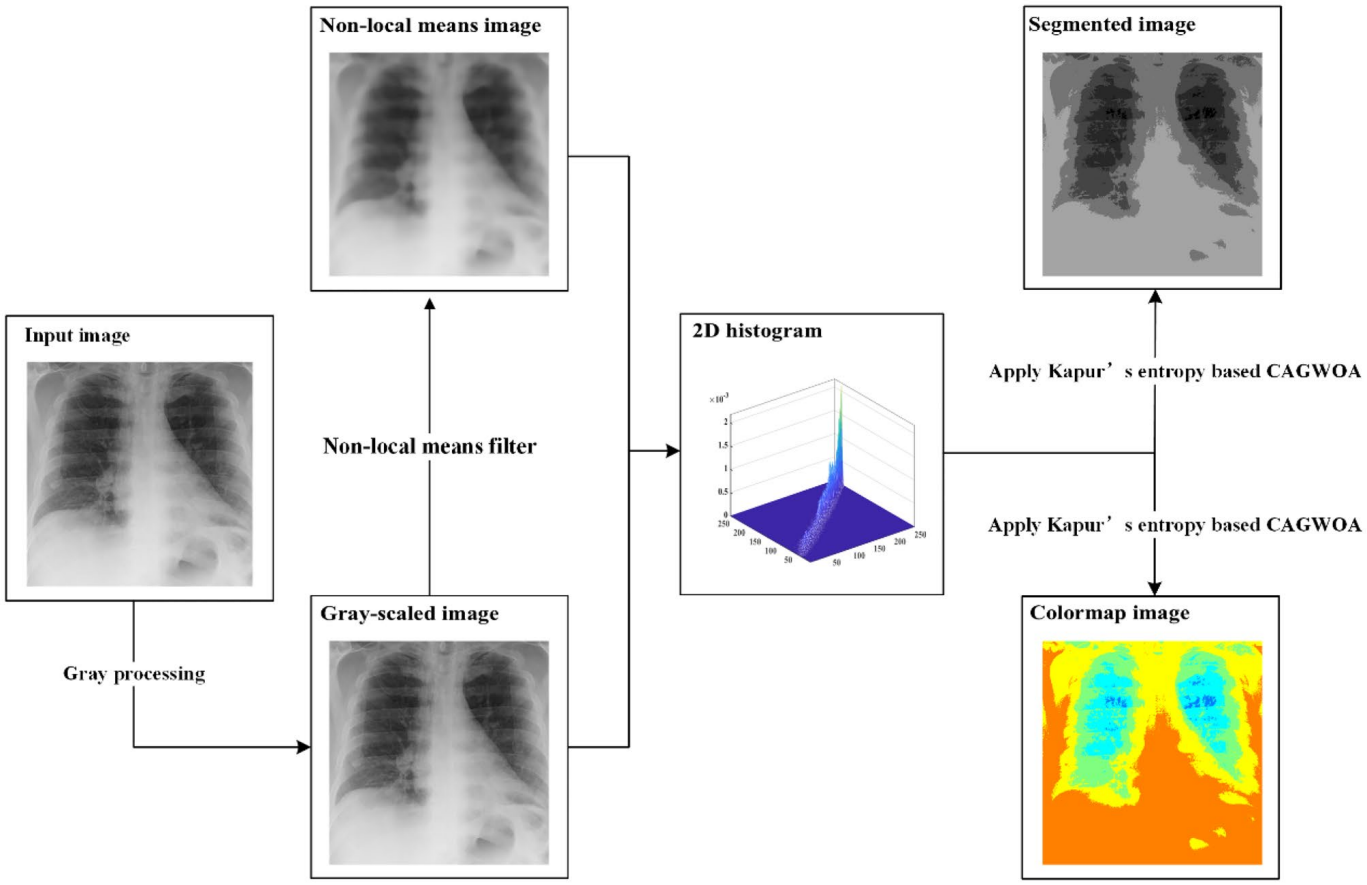
Image segmentation flow chart.

**Figure 2:**
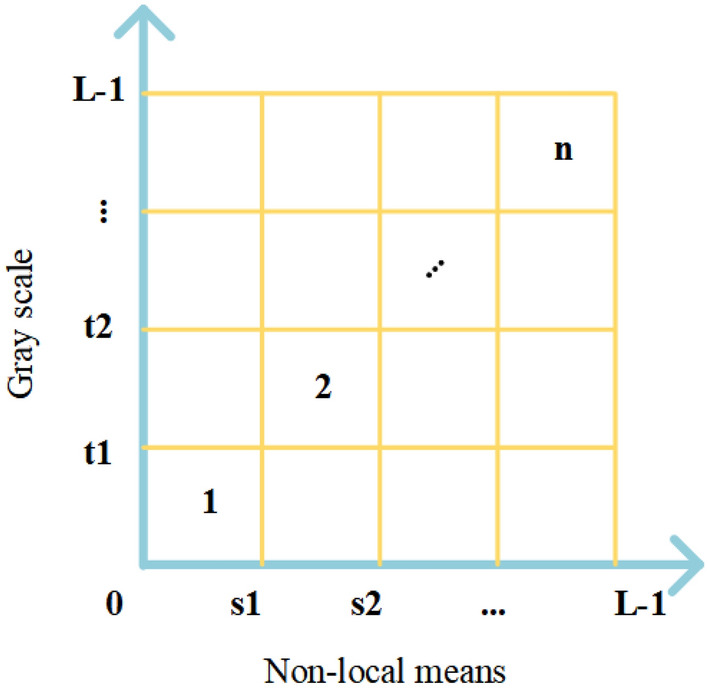
Plan view of the 2D distribution histogram.

The grayscale image $I(x,y)$ of size $M\times N$ with gray levels in the range $[0,L-1]$ is processed by nonlocal mean filtering, and the image $g(x,y)$ of the corresponding size and gray level range can be obtained. Using $i$ to denote the grayscale value of pixels in the grayscale image $I(x,y)$ and $j$ to denote the nonlocal mean of pixels in the nonlocal mean image $g(x,y)$, $i$ and $j$ can be integrated to form a 2D distribution histogram $h(i,j)$. The final 2D distribution histogram is obtained after normalizing $h(i,j)$ using Eq. (5). This image segmentation model, the flow, is shown in Fig. 1. A plan view of the normalized 2D distribution histogram is shown in Fig. 2.

5 $${P}_{ij}=\frac{h(i,j)}{M\times N}.$$

#### 2D Kapur’s entropy

According to Fig. 1, the 2D distribution histogram's primary diagonal includes the majority of the image's data. In order to streamline the computation, the Kapur’s entropy of MTIS is only computed for the $n$ areas on the major diagonal. In the 2D distribution histogram design shown in Fig. 2, the gray levels are denoted by $\left\{{t}_{1},{t}_{2},{t}_{3}\dots L-1\right\}$, and the nonlocal mean is calculated using $\left\{{S}_{1},{S}_{2},{S}_{3}\dots L-1\right\}$.

The 2D Kapur’s entropy corresponding to the 2D distribution histogram can be calculated by Eq. (6). Where, ${P}_{ij}$ denotes the corresponding point on the 2D distribution histogram. The gray level $\left\{{t}_{1},{t}_{2},{t}_{3}\dots L-1\right\}$ is taken as the objective function, and the 2D Kapur’s entropy $\varphi (s,t)$ is maximized as the optimal threshold by CAGWOA.

6 $$\varphi \left(s,t\right)=-\sum_{i=0}^{{s}_{1}} \sum_{j=0}^{{t}_{1}} \frac{{P}_{ij}}{{P}_{1}}\text{ln}\frac{{P}_{ij}}{{P}_{1}}-\sum_{i={t}_{1}+1}^{{s}_{2}} \sum_{j={t}_{1}+1}^{{t}_{2}} \frac{{P}_{ij}}{{P}_{2}}\text{ln}\frac{{P}_{ij}}{{P}_{2}}-\cdots -\sum_{i={s}_{L-2}+1}^{{s}_{L-1}} \sum_{j={t}_{L-2}+1}^{{t}_{L-1}} \frac{{P}_{ij}}{{P}_{L-1}}\text{ln}\frac{{P}_{ij}}{{P}_{L-1}}.$$

### The overview of WOA

Whale optimization algorithm (WOA) inspired by the behavior of whale populations has become a typical representative of swarm intelligence optimization algorithms with its simplicity and efficiency. After the individuals in the WOA population are randomly initialized, the positions of the individuals in the population are randomly updated through the following three phases in each iteration. These three phases are: searching for prey, surrounding prey, and attacking prey.

The equation for updating the position of the individual during the phase of searching for prey can be described as Eq. (7).

7 $${X}_{it+1}={X}_{rand}-A\times D,$$

8 $$D=\left|C\times {X}_{rand}-{X}_{it}\right|.$$

The position of the individual in the solution space can be represented as $X$ in Eq. (7), the current number of iterations is denoted as $it$. ${X}_{rand}$ denotes the random individuals in the population. $D$ denotes the distance between the current individual $X$ and the random individual ${X}_{rand}$. In Eq. (8), $C$ is the weight of the random individual ${X}_{rand}$. $C\in [\text{0,2}]$, which is used to control the distance between $X$ and ${X}_{rand}$. $A$ is a random value, and $A\in [-\text{2,2}]$. According to the value of $A$, individuals within the population randomly choose to search or surround the prey. When $A<-1$, or $A>1$. In the current iteration, the position of individual ${X}_{it+1}$ in the population is updated by searching for prey. In addition, if $-1<A<1$, the individual enters the prey encirclement phase.

In the surround prey phase, the position of the individuals is updated by Eq. (9).

9 $${X}_{it+1}={X}_{best}-A\times \left|C\times {X}_{best}-{X}_{it}\right|.$$

In Eq. (9), ${X}_{best}$ represents the best optimal individual in the current iteration. In the update phase of prey encirclement, individuals will randomly contract toward ${X}_{best}$. In addition to the two location update ways above. the individuals in the population randomly chooses to feed in a spiral contraction to get closer to the optimal individual ${X}_{best}$. The prey attack and spiral search phases can be described as Eq. (10).

10 $${X}_{it+1}={D}_{best}\times {e}^{bl}\times \text{cos}\left(2\pi l\right)+{X}_{it},$$

11 $${D}_{best}=\left|{X}_{best}-{X}_{it}\right|.$$

Equation (10) abstracts the behavior of a whale bubble net attack, where the whales produce bubbles that spiral upward to wrap around their prey. ${D}_{best}$ indicates the distance between the best optimal individual position ${X}_{best}$ in the current iteration and the current individual position ${X}_{it}$ as Eq. (11). The logarithmic spiral’s shape is determined by the constant $b$, and $b=1$. $l$ is a random number and $l\in [-\text{1,1}]$. During the prey attack phase, the individuals within the population gradually approach the optimal individual by contracting in spiral.

## The proposed CAGWOA

The CAGWOA mentioned in this paper is based on WOA and combines the COS sampling initialization strategy (COSI), adaptive global search strategy (GS), and all-dimensional neighborhood mechanism (ADN). The introduction of the COSI solves the problem that WOA tends to fall into the local optimum when dealing with multimodal and complex mixed functions to enhance the stability of the image segmentation model. In addition, the GS expands the search range of individuals and enhances the exploration capability of the algorithm to improve the segmentation efficiency. The ADN further explores the optimal individual adjacency region. It enhances the algorithm's capacity for local exploration and helps increase the accuracy of the result to enhance the accuracy of the image segmentation model.

### COS sampling initialization strategy

**Figure 3:**
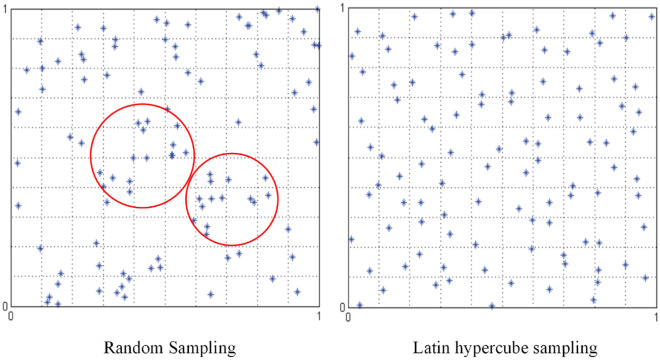
Comparison chart of sampling results.

Traditional initialization methods are created based on random numbers and are memoryless. The state of previous individuals is not taken into account when generating new individuals, and the distribution is highly uncertain. It is easy to cause the aggregation of the population in the initial state, which leads the algorithm to fall into local optimum easily in solving multimode functions and complex optimization problems. In Fig. 3, Figure a show the distribution results of creating 100 individuals by the traditional random number-based population initialization method in the range of $[\text{0,1}]$ under the two-dimensional space. The individuals do not cover the entire solution space uniformly, producing the aggregation phenomenon in the red circle.

The main idea of LHS is the stratification of probability distributions. The solution space is divided into equal subspaces by stratification. Subsequently, a random sample is selected in each stratification using a probability density function. Generalizing this concept to arbitrary dimensions ensures that each sample is unique in the axial hyperplane containing him. In swarm intelligent optimizations algorithms, the LHS-based initialization method can effectively avoid the problems of the initialization and iteration processes that are prone to local optimums and deception of the objective function^44^. In Fig. 3, Figure b shows the LHS-based initialization results. Compared with the traditional random number-based initialization method, the individuals in the sampling space achieve a more uniform distribution and reduce aggregation.

However, the LHS-based initialization method is limited by the number of samples and the range of the solution space. When the number of individuals in the population is limited or the range of the solution space is large, its performance is greatly affected, at which time the initialization method based on random numbers is more advantageous^95,96^. Therefore, it is necessary to introduce a certain degree of randomness based on LHS. In this paper, COSI is designed. It can be described as Eq. (12).

12 $${X}_{i}=\text{cos}\left(\pi \times \left(\frac{1}{2}-{p}_{i}\right)\times b\right)\times \left(ub-lb\right)+lb.$$

${X}_{i}$ is the position of the initialized $ith$ individual in the population. The dimension $D$ of the problem is taken as the number of strata in the LHS, and the population size $N$ is taken as the number of samples in the LHS. ${p}_{i}$ is the sequence of LHS corresponding to the individual ${X}_{i}$ generated by the LHS, ${p}_{i}=[{p}_{i,1},{p}_{i,2},{p}_{i,3}\dots {p}_{i,D}]$. Where any dimension component ${p}_{i,j}$ is not only unique in ${p}_{i}$, but still unique on the corresponding jth dimension component ${p}_{:,j}=[{p}_{1,j},{p}_{2,j},{p}_{3,j}\dots {p}_{N,D}]$ of the LHS sequence. $b$ is a random number to control the randomness of the initialization. $b\in [\text{0,1}]$, generated independently on each dimension component. $ub$ and $lb$ are the upper and lower bounds of the search space.

In this paper, we propose COSI, which improves the metaheuristic algorithm's random number-based initialization method by combining the advantages of LHS-based and random number-based initialization methods. To some extent, the algorithm achieves better solution space coverage with the same number of individuals. It helps to reduce the aggregation phenomenon of the initialization state of the population, improve the stability of the algorithm on multimode functions and complex optimization problems, and avoid the algorithm from falling into the local optimum easily.

### Adaptive global search strategy

The GS is used to enhance the global search capability of WOA. According to Eq. (13), ${X}_{i}^{j}$ is the position of $ith$ individual ${X}_{i}$ in $jth$ dimension. The position of ${X}_{i}^{j}$ is updated by the best individual position ${X}_{best}^{j}$ in the same dimension and two different random individual positions ${X}_{rand1}^{j}$ and ${X}_{rand2}^{j}$ in the population.

13 $${X}_{i}^{j}={X}_{best}^{j}+2\times C\times ({X}_{rand1}^{j}-{X}_{rand2}^{j})$$

The parameter $C$ is used to control the weights of random individuals, $C\in [\text{0,2}e]$. $C$ gradually increases as the number of assessments increases. To prevent individuals from stagnating in the local optimum, the exploration step is increased in the later assessment stages. $b$ is a random number, $b\in [\text{0,1}]$.

14 $$C=2\times b\times {e}^\frac{FEs}{MaxFEs}$$

In the early stage of algorithm evaluation, the optimal individual has more influence on the result; however, as the number of evaluations increases, the influence of random individuals gradually increases, which helps avoid the algorithm from falling into local optimum.

### All-dimensional neighborhood mechanism

To further improve the exploration ability of individuals in WOA. We try to introduce the ADN. The first proposal of the ADN was applied to PSO to enhance the local search ability of PSO individuals for adjacent spaces^97^. Inspired by this, we introduce the ADN into WOA.

ADN creates a agent of $2\times dim$ individuals to explore the space adjacent to the $dth$ dimension of the optimal individual ${X}_{best}^{d}$. When $k=d$, the individual's ordinal number $k$ is equal to the current dimension $d$. The agent individuals update by Eq. (15) and Eq. (16). $L$ is the step size of the individual's exploration of the adjacent space. individuals in the ADN agent population enhance the local exploration around the optimal individual by adding a random step size to the positions adjacent to the optimal individual.

15 $${NP}_{2\times k-1}^{d}= {X}_{best}^{d}+L,$$

16 $${NP}_{2\times k}^{d}= {X}_{best}^{d}-L.$$

When $k\ne d$, the individuals in the ADN population are updated by Eqs. (17) and (18). The individuals in the ADN backup population retain the position information of the optimal individual corresponding to the dimension.

17 $${NP}_{2\times k-1}^{d}={X}_{best}^{d},$$

18 $${NP}_{2\times k}^{d}={X}_{best}^{d}.$$

The exploration step $L$ has three types of updates: contraction, expansion, and maintenance of the current state. $s$ is the contraction factor of the exploration step $L$. In the contracted state of step $L$, the individuals in the ADN population gradually approach the optimal individuals. This further increases the convergence speed of the algorithm.

19 $$L=L\times s.$$

If $L<{L}_{min}$, $L$ expands outward according to Eq. (14). ${L}_{0}$ is the value of the initial state of $L$. $r$ is a random number, $r\in [\text{0,1}]$. When the step size $L$ shrinks to a certain degree, $L$ will expand outward and reinitialize a random step size. The problem of local optimum caused by too close to the optimal individual is avoided.

20 $$L={L}_{0}\times r.$$

The design of step size $L$ effectively improves the local exploitation ability of the population around the optimal individuals. Then the better fitness values and positions of individuals within the original and ADN agent populations are retained by greedy selection.

### Overall algorithm

**Figure 4:**
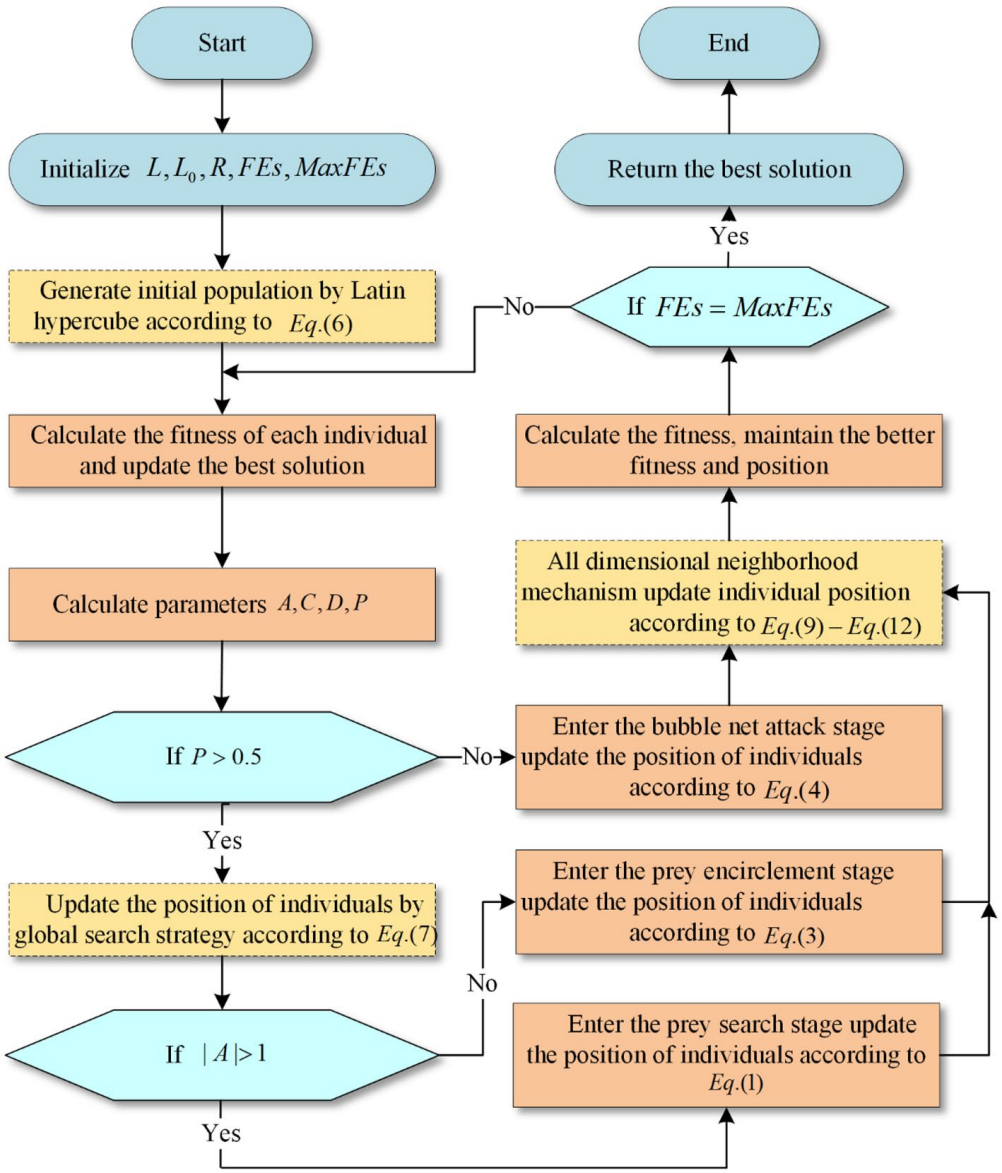
Flow chart of proposed CAGWOA.

In this section, the improved CAGWOA based on WOA is introduced in detail. CAGWOA introduces COS sampling initialization, an adaptive global search strategy, and an ADN based on the standard WOA to improve the phenomenon of population aggregation in the initial state of WOA and the defects of easy fall into local optimum and poor exploitation ability during iteration, to cope with the problems of low segmentation accuracy, slow efficiency and poor stability exhibited by WOA in image segmentation modeling. The three mechanisms introduced in CAGWOA are shown in Fig. 4. The three mechanisms introduced in CAGWOA are shown in the flowchart of the algorithm by highlighting them in yellow. The details of the algorithm are shown in the pseudocode of Algorithm 1 CAGWOA.

First, CAGWOA uses the COSI proposed in Sect. "COS sampling initialization strategy" to replace the random number-based initialization method in WOA. COS sampling initialization ensures uniform coverage of the search space by the CAGWOA population to enhance the quality of the initial population solution and avoid the problems of not covering the global search space and falling into local optimality caused by population initialization aggregation in high-dimensional complex problems. In multi-threshold image segmentation, the initialization state of the algorithm has a great influence on the subsequent convergence process and affects the stability of the segmentation results.

Second, CAGWOA introduces the GS introduced in Sect. "Adaptive global search strategy" before the update phase of WOA, searching for prey and enclosing prey. The GS expands the local scope of individuals and ensures the algorithm's ability to explore the solution space. And as the iterative process advances, the search weights are subsequently reduced, which lays the foundation for the subsequent algorithm development. The increased convergence speed of the algorithm helps to improve the segmentation efficiency of multi-threshold image segmentation.

Finally, CAGWOA introduces the ADN introduced in Sect. "All-dimensional neighborhood mechanism" after the three update phases of the original WOA are completed. The ADN is adapted to develop around the space of near-optimal solutions around the optimal individuals in the population to obtain higher-quality solutions. The quality of the solution determines the segmentation accuracy of multi-threshold image segmentation.

**Algorithm 1:**
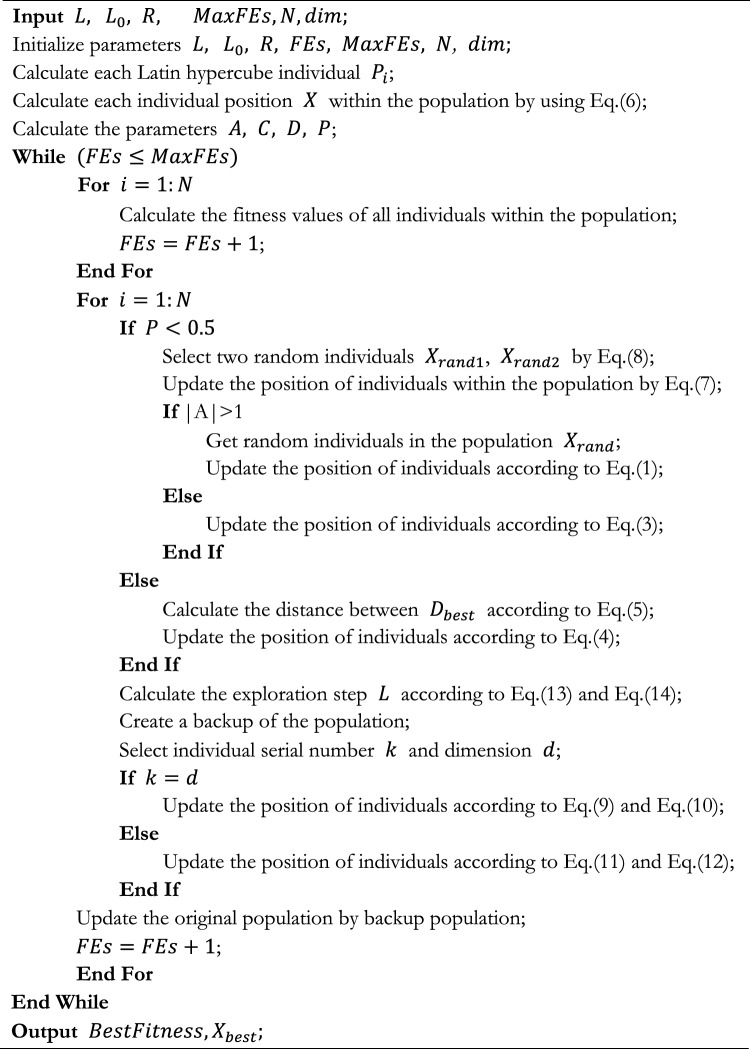
Pseudocode of CAGWOA.

CAGWOA’s time complexity is mainly determined by the maximum number of iterations $(E)$, the population size $(N)$, the dimension size $(d)$ and the calculation of objective function value $(F)$. By analysis, the overall algorithmic time complexity of CAGWOA is O (CAGWOA) = O (COSI) + O (Initialize the objective function value of the population) + *E* × (O (GS) + O (Update the position of the population) + O (Calculate the objective function value of the population) + O (Update the objective function value of the population) + O (ADN) + O (Make a greedy choice between ADN's population and original population)).

The time complexity of COSI is $O(N\times d)$. The time complexity required to initialize the objective function value of the population is $O(N\times F)$. The time complexity of GS is: $O(N)$. The time complexity required to update the positions of the population is: $O(N\times d)$. The time complexity of calculate the objective function value of the population is: $O(N\times F)$. The time complexity required to update the objective function value is: $O(N)$. The time complexity required for the ADN is $O\left({(2\times d)}^{2}\right)$. The time complexity required to make a greedy choice between ADN's population and original population is: $O(2\times d)$. Therefore, the overall time complexity of CAGWOA is: $O\left(C\text{AGWOA}\right)=O\left(N\times \left(d+F\right)+E\times (N\times \left(2+d+F\right)+{\left(2\times d\right)}^{2}+2\times d)\right)$.

## Benchmark experimental results and discussion

In this section, to validate the performance of the proposed CAGWOA, CAGWOA was subjected to a range of experiments, including ablation experiment, comparison with traditional algorithms, comparison with some improved algorithms, and comparison with well-known WOA variants.

### Experimental settings

The benchmark function experiments were conducted using the IEEE CEC 2014^45^, IEEE CEC 2019^46^, and IEEE CEC 2022^47^ test sets. IEEE CEC 2014, a classical test set, includes 30 single-objective functions. IEEE CEC 2022 offers 12 more complex functions. These functions fall into four categories: single-modal, simple multimodal, hybrid, and composite. Single-modal functions contain only one minimum value, used to test the algorithm's exploitation capability. Simple multimodal functions contain multiple local minima and one global minimum, used to test the algorithm's exploration and local optima avoidance. Hybrid functions and composite functions are concatenated and combined based on single-modal functions and simple multimodal functions, used to test the algorithm's ability to handle depth and complexity. The relevant details of the functions are shown in Table A.1 in the Appendix.

The experiments in this paper were conducted under the limit of the maximum number of evaluations ($MaxFEs$), and to ensure the fairness of the experiments, all the algorithms involved in the comparison were evaluated by adding one to the number of $FEs$ after one separate fitness value calculation. All experiments were conducted in the same environment and settings. The algorithms involved in the comparison in the experiments all used the best parameters set in their original papers.

In this paper, the maximum number of evaluations $MaxFEs$ is set to 300,000 and the size of the population is set to 30. To reduce the effect of randomness in the experimental results, all algorithms are tested on the benchmark function set 30 times. In addition, in order to evaluate the experimental results comprehensively and further validate the performance of the CAGWOA, the experimental results were further analyzed using average value (AVG), standard deviation (STD), Wilcoxon signed-rank (WSRT)^98^ and Friedman test (FT)^99^.

In addition, to ensure the same experimental environment for all experiments, the experiments were conducted on Windows Server Windows 11. The processors were coded using an Intel i5-12500H with a 12-core processor (2.50 GHz) and 16 GB of RAM, using Matlab2021b.

### Sensitivity analysis of parameter ${\varvec{\rho}}$

In this section's experiments, a parameter sensitivity analysis of the execution probability $\rho$ of AND was conducted within the range $[\text{0.1,1.0}]$ with an interval of 0.1. Appendix Table A2 illustrates the AVG and STD values of convergence results on benchmark functions for different settings of $\rho$ values in various versions of CAGWOA. Bold data highlight the $\rho$ settings with the highest precision and stability in specific test functions. By assessing the significance of performance differences among all CAGWOA versions on different benchmark functions, FT ranks all CAGWOA versions for each benchmark function. The average ranking and final ranking of algorithm performance across all benchmark functions are calculated to present FT's statistical results in Table 2.

**Table 2:** The results of the FT analysis of the parameter $\rho$.

$\rho$	0.1	0.2	0.3	0.4	0.5	0.6	0.7	0.8	0.9	1
Avg	5.267	4.667	4.933	4.967	5.000	4.900	4.700	4.633	4.567	4.167
Rank	10	4	7	8	9	6	5	3	2	1

Specifically, Avg displays the average ranking of CAGWOA versions with different settings of $\rho$ values across all benchmark functions. The similar average rankings of all CAGWOA versions indicate that CAGWOA is not sensitive to the setting of the $\rho$ parameter. The final ranking results reveal that the algorithm performs best when $\rho$ is set to 1.0. Therefore, in the experimental process, the $\rho$ value is set to 1.0.

### Ablation experiment

The experiments in this section are used to compare the effect of different introduced strategies on the performance of the algorithm and further analyze the role of different strategies. This is due to the fact that the combination of different strategies is not a simple superposition of effects. The combination of core formulas between different strategies may have either a negative or positive impact on the performance of the algorithm. To evaluate the effect of the combination between different strategies on WOA, this experiment was designed. And the COS sampling initialization strategy, the adaptive global search strategy, and the all-dimensional neighborhood mechanism are named ‘COSI’, ‘GS’ and ‘AND’, respectively. Eight different versions of these three strategies combined with WOA are shown in Table 3. Where, ‘0’ means that the strategy is not introduced in WOA, and ‘1’ means that the strategy is introduced. For example, the column ‘COSI’ in AGWOA is ‘0’, while the columns ‘AND’ and ‘GS’ are ‘1’ means that the all-dimensional neighborhood mechanism and adaptive global search strategy are introduced in AGWOA, but the COS sampling initialization strategy is not introduced.

**Table 3:** Versions of various CAGWOAs.

	CAGWOA	AGWOA	CGWOA	CAWOA	GWOA	AWOA	CWOA	WOA
COSI	1	0	1	1	0	0	1	0
GS	1	1	1	0	1	0	0	0
ADN	1	1	0	1	0	1	0	0

Table A.3 in the Appendix presents the experimental results of eight variants of the WOA algorithm combined with three strategies under the 30 benchmark functions of IEEE CEC 2014. The AVG and STD for each test functions reflect the stability and accuracy of the algorithm's search. The algorithm with smaller STD values exhibited more stable search performance, while the algorithm with smaller AVG values showed higher precision. The result shows that CAGWOA has better stability and better accuracy than other variants of WOA on most of the 30 benchmark functions.

In Table 4, ‘ + /–/ = ’ is the test result of WSRT in the experiment, Mean is the analysis result of FT, which indicates the average rank of the algorithm's result among the 30 benchmark functions, and Rank indicates the rank of the algorithm among all the participating comparison algorithms. In the table, ‘ + ’ indicates that CAGWOA outperforms the compared algorithms, and ‘-’ indicates that CAGWOA underperforms the compared algorithms. ‘ = ’ indicates that the performance of CAGWOA is comparable to the compared algorithms. In the test results of FT, CAGWOA ranked first. Among the 30 benchmark functions, CAGWOA outperforms CGWOA in 6 benchmark functions, is inferior to CGWOA in 3 benchmark functions, and has comparable performance with CGWOA in 18 benchmark functions. The results show that CAGWOA has the best performance by introducing COSI, GS and ADN in WOA.

**Table 4:** Ablation experiment results of WSRT and FT.

	CAGWOA	WOA	CWOA	AWOA	GWOA	CAWOA	CGWOA	AGWOA
+ /–/ =	~	27/1/2	18/6/6	23/1/6	18/1/11	13/7/10	6/3/21	17/0/13
Mean	2.3333	6.9667	4.4000	5.7333	4.9000	3.7000	2.7667	4.8667
Rank	1	8	4	7	6	3	2	5

**Figure 5:**
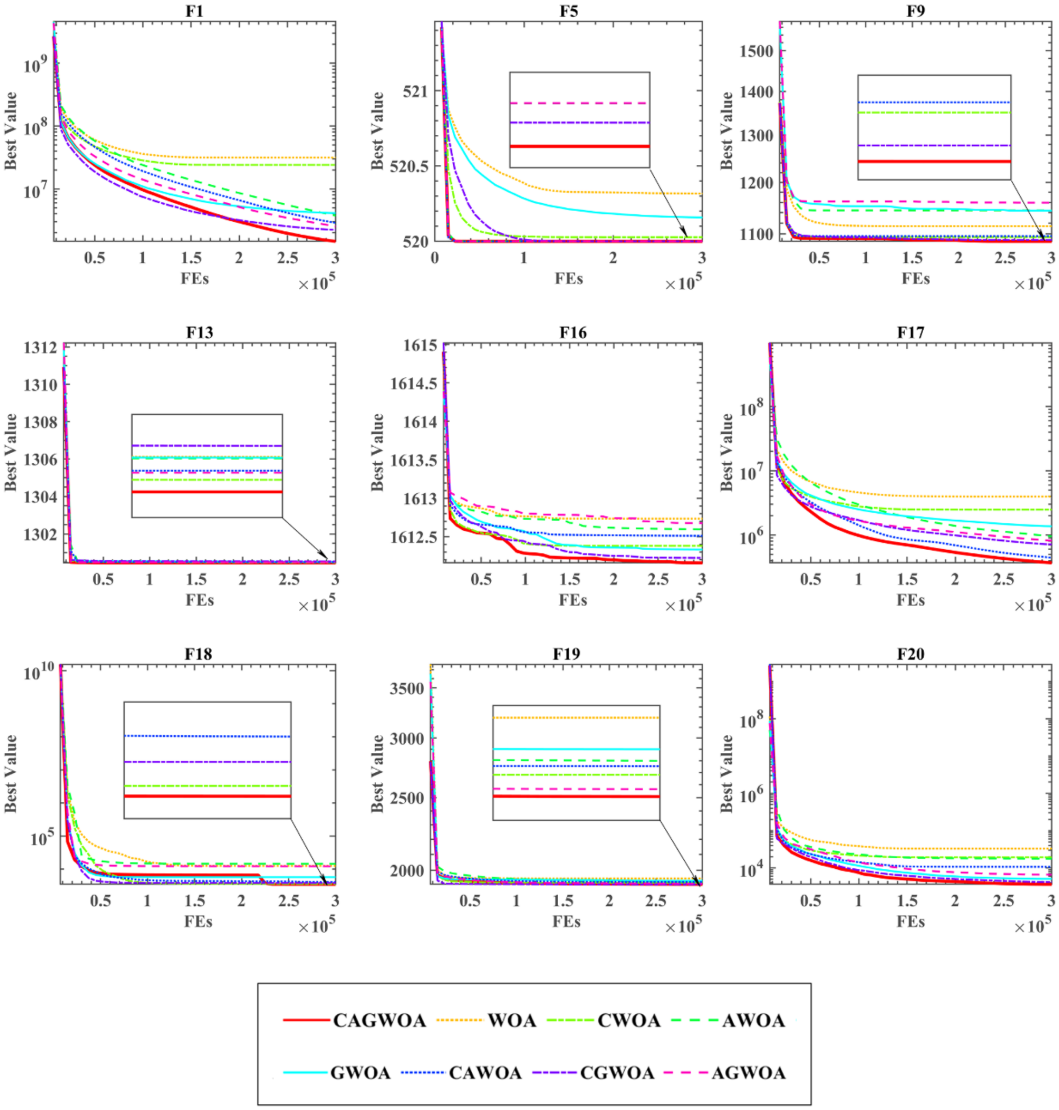
Convergence image of the partial function of the ablation experiment.

Figure 5 shows the iterative images of WOA and WOA combined with 7 variants of 3 strategies on 9 functions, which are F1, F5, F9, F13, F16, F17, F18, F19, and F20. CAGWOA has the fastest convergence speed and excellent convergence results on F1, F5, F16, F17, and F20 and is able to obtain more excellent convergence results. From the convergence curve of F16, when the number of evaluations is between 5,000 and 15,000, the other algorithms have a slower convergence rate and poorer exploration ability, but CAGWOA still maintains a faster convergence rate and jumps out of the local optimum. The convergence result of CGWOA is second only to CAGWOA, which shows that the introduction of the ADN effectively enhances the exploitation ability of the WOA. On the iteration curves of F9, F13, F18, and F19 functions, the convergence speed of some WOA variants is similar to that of CAGWOA, but CAGWOA always gains more accurate convergence results and higher quality solutions.

In summary, CAGWOA has better results in terms of convergence speed and convergence accuracy compared to other WOA combinatorial variants.

### Comparison with some excellent peers

In order to compare the differences between CAGWOA and other advanced optimization algorithms in dealing with different functions. In this section, we compare the enhanced WOA with seven other well-known metaheuristics and the original WOA. These seven algorithms are: (PSO)^23^, Sine Cosine algorithm (SCA)^18^, bat optimization algorithm (BA)^20^, gray wolf optimization algorithm (GWO)^25^, firefly optimization algorithm (FA)^24^, wind driven optimization algorithm (WDO)^16^, and moth-flame optimization algorithm (MFO)^17^. Table A.4 in the appendix shows the AVG and STD of CAGWOA and the other eight methods. The data in the table show that CAGWOA has stable search performance on most unimodal functions, better stability on multimodal functions, and the ability to obtain better quality solutions.

According to Table 5's average ranking, CAGWOA performs noticeably better than the other algorithms. Based on the result of ‘ + /–/ = ’, it can be concluded that CAGWOA outperforms other algorithms on most functions.

**Table 5:** The analysis results of WSRT and FT.

	CAGWOA	WOA	PSO	SCA	BA	GWO	FA	WDO	MFO
+ /-/ =	~	28/1/1	19/9/2	29/0/1	20/6/4	20/8/2	30/0/0	19/6/5	26/2/2
Mean	2.5733	5.2833	4.0478	7.1911	4.3300	4.0289	7.5589	4.5467	5.4400
Rank	1	6	3	8	4	2	9	5	7

**Figure 6:**
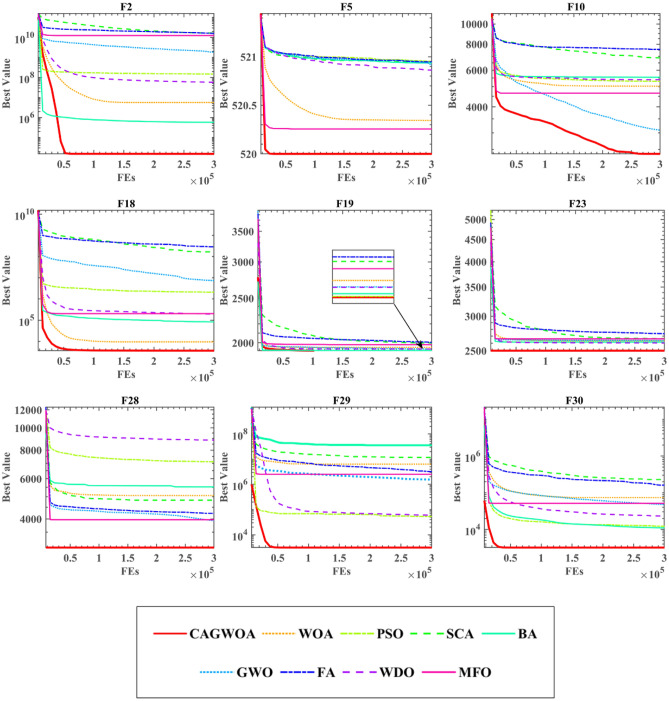
Convergence curve of CAGWOA with excellent peers.

Figure 6 shows the convergence curves of CAGWOA with other algorithms for the 9 functions F2, F5, F10, F18, F19, F23, F28, F29, and F30 out of the 30 benchmark functions of IEEE CEC 2014. Observing the graphs, it can be concluded that the CAGWOA can find the optimal solution quickly throughout the operation, showing an efficient search capability. The algorithm finds the optimal solution in the initial state when processing F23 and F28. With F10, we can see that CAGWOA still maintains a strong exploration capability when other algorithms fall into local optimum and no longer converge.

To sum up, CAGWOA compares with other algorithms. It has stronger exploration and exploitation ability, and can effectively avoid falling into local optimum.

### Comparison with other improved algorithms

To measure the performance of CAGWOA with other improved algorithms in dealing with different functions. In this section, in order to completely validate the performance difference of CAGWOA compared to other improved algorithms, in addition to the 30 classic test function functions of IEEE CEC 2014, this section is supplemented with comparative experiments on a total of 22 more complex and challenging test functions of IEEE CEC 2019 and IEEE CEC 2022. The experiments are compared with eight other excellent improved algorithms. These eight algorithms are fusion optimization algorithm for SCA and PSO (ASCA_PSO)^100^, chaotic bat algorithm (CBA)^101^, Cauchy and Gaussian improved sine cosine optimization algorithm (CGSCA)^102^, hybrid grey wolf optimizer (HGWO^103^, adaptive mutation improved fruit fly optimization algorithm (AMFOA)^104^, the opposition-based learning improved positive cosine optimization algorithm (OBSCA)^105^, the chaotic fruit fly optimization algorithm (CIFOA)^106^, and the optimization algorithm for fusion of DE and MFO (DSMFO)^107^. Table A.5 presents the results of CAGWOA and eight other algorithms on 30 functions of IEEE CEC 2014, including STD and AVG values. The analysis shows that CAGWOA has the smallest variance and mean value among most of the benchmark functions. This indicates that CAGWOA has a more stable optimization efficiency and can obtain more accurate optimization results compared to the other eight algorithms.

Table 6 shows the WSRT and FT analysis results of CAGWOA and the other 8 algorithms. From the results of FT, it can be seen that CAGWOA ranks first. Among the results of WSRT, CAGWOA has better optimization results than AMFOA on 29 of the 30 benchmark functions of IEEE CEC 2014 compared to AMFOA.

**Table 6:** The analysis results of WSRT and FT.

	CAGWOA	ASCA_PSO	CBA	CGSCA	HGWO	AMFOA	OBSCA	CIFOA
+ /–/ =	~	25/1/4	21/3/6	24/6/0	25/3/2	29/1/0	28/1/1	23/7/0
Mean	2.4206	3.8844	3.9489	4.3761	4.1844	7.9756	5.7867	6.7550
Rank	1	2	3	5	4	9	7	8

**Figure 7:**
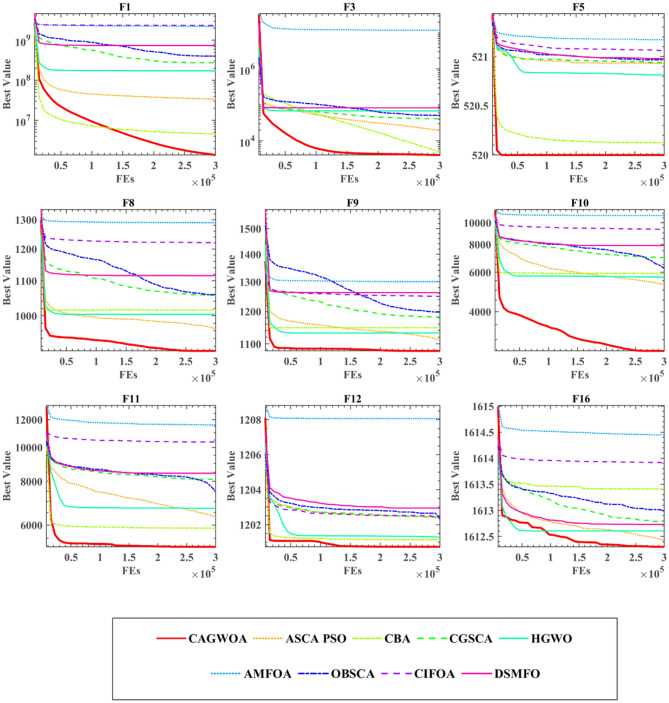
Convergence curve of comparison improved algorithms at IEEE CEC 2014.

Figure 7 shows the iteration curves of CAGWOA with the other eight improved algorithms on the nine benchmark functions of IEEE CEC 2014. From the results in the figure, CAGWOA has a stronger ability to search and obtain better solutions compared to the other algorithms. From the convergence curves of F5, F8, F9, F10, F11, and F12, compared with other improved algorithms, CAGWOA relies on its strong exploration ability to obtain relatively optimal solutions in the early stage of convergence, and maintains its exploitation ability when other algorithms gradually fall into local optimality in the later stage. In F1 and F16, although the CAGWOA did not find better solutions in the early stage, and relied on its strong exploitation ability, the accuracy and quality of the solutions obtained by CAGWOA were gradually higher than those of other algorithms. And this strong exploitation capability is maintained throughout the evaluation process.

In summary, CAGWOA compares with other improved algorithms. It has stronger global exploration ability and relies on the stable exploration ability to obtain higher quality solutions.

Table A.6 presents the AVG and STD values derived from the comparative analysis between CAGWOA and other enhanced algorithms at IEEE CEC 2019&2022. Notably, despite the heightened complexity of the optimization problems, CAGWOA consistently demonstrates smaller mean and variance values across a majority of the function problems. This underscores the robustness and superiority of CAGWOA, sustaining its competitive edge over the other eight optimization algorithms even when confronted with increasingly intricate and demanding optimization challenges.

In Table 7, the WSRT and FT results from the comparative experiments at IEEE CEC 2019&2022 are presented. Even as the complexity of the optimization objectives escalates, CAGWOA's FT performance consistently secures the top position. When juxtaposed with the WSRT experimental outcomes involving the comparison algorithms at IEEE CEC 2014, CAGWOA continues to maintain an advantageous position across most function problems. While the margin over ASCA_PSO, CBA, CGSCA, and HGWO has narrowed, this still substantiates the adaptability of CAGWOA across optimization challenges of varying complexity levels.

**Table 7:** The analysis results of WSRT and FT on IEEE CEC 2019&2022.

	CAGWOA	ASCA_PSO	CBA	CGSCA	HGWO	AMFOA	OBSCA	CIFOA	DSMFO
+ /–/ =	~	12/7/3	18/2/2	15/2/5	13/6/3	21/0/1	19/1/2	20/0/2	20/0/2
Mean	2.0909	3.5455	5.5455	4.0000	3.0000	8.5000	4.7727	7.1364	6.0909
Rank	1	3	6	4	2	9	5	8	7

**Figure 8:**
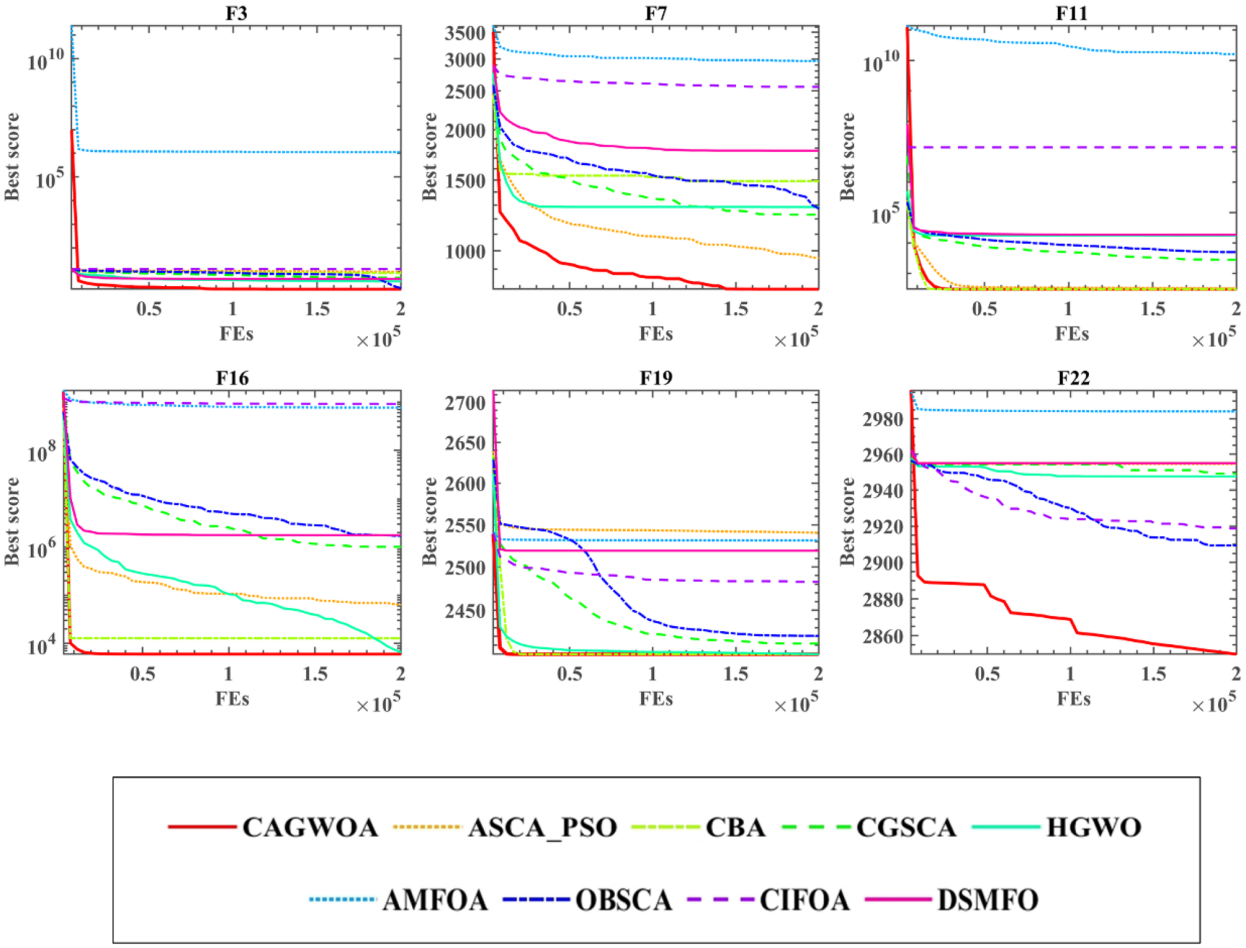
Convergence curve of comparison improved algorithms at IEEE CEC 2019&2022.

Figure 8 illustrates the convergence curves of CAGWOA juxtaposed with other algorithms across a selection of the 22 test functions from IEEE CEC 2019&2022. Notably, in comparison to the reference algorithms, CAGWOA exhibits pronounced exploratory capabilities on functions F3, F11, F16, and F19. It manifests a propensity for precise solution exploration early in the optimization process. Conversely, on F7 and F22, where many algorithms tend to converge prematurely towards local optima, CAGWOA distinguishes itself with its robust exploratory prowess. Leveraging this strength, CAGWOA demonstrates a consistent developmental trajectory, with its convergence accuracy progressively enhancing as the iterative process unfolds.

In summary, CAGWOA compares to other improved algorithms. Both on the classic IEEE CEC 2014 function test set and on the more complex and challenging IEEE CEC 2019 and IEEE CEC 2022 show some advantages. During the convergence process, CAGWOA demonstrates enhanced global exploration capabilities, complemented by its stable exploitation prowess, culminating in the acquisition of superior-quality solutions.

### Comparison with other WOA variants

To estimate the differences between CAGWOA and other WOA variants, in this section compared CAGWOA with seven other WOA variants and the original WOA. Include: the improved WOA with backward learning (OBWOA)^108^, the improved WOA fusing Lévy flight strategy and quadratic interpolation method (MWOA)^109^, the improved WOA with Lévy flight (LWOA)^110^, the WOA with artificial swarm hybrid (ACWOA)^111^, improved WOA with chaotic local search (BWOA)^112^, improved WOA based on chaotic initialization strategy, Gaussian variation and chaotic local search strategy (CCMWOA)^113^and improved WOA based on learning (BMWOA)^114^.

Table A.7 in the Appendix shows the AVG and STD values of the WOA variants on the 30 benchmark functions of IEEE CEC 2014. CAGWOA achieves the smallest mean and variance on most of the benchmark functions. This indicates that CAGWOA has higher stability compared to other WOA variants. Table 8 demonstrates the comparison results of WSRT and FT. The ‘ + /–/ = ’ from Table 8 shows the significant advantage of CAGWOA. For example, when comparing MWOA, the result is ‘30/0/0’, which indicates that CAGWOA outperforms MWOA for each of the 30 benchmark functions of IEEE CEC 2014. in terms of the average ranking of the results, CAGWOA ranks first, which indicates that CAGWOA compared to the other WOA variants is the best choice.

**Table 8:** WOA variants comparison results of WSRT and FT.

	CAGWOA	OBWOA	MWOA	LWOA	ACWOA	BWOA	CCMWOA	BMWOA	WOA
+ /–/ =	~	21/2/7	30/0/0	15/6/9	26/2/2	21/5/4	23/7/0	28/0/2	27/0/3
Mean	2.5594	4.1933	8.9211	3.3833	5.7294	4.1822	6.0467	5.2511	4.7333
Rank	1	4	9	2	7	3	8	6	5

**Figure 9:**
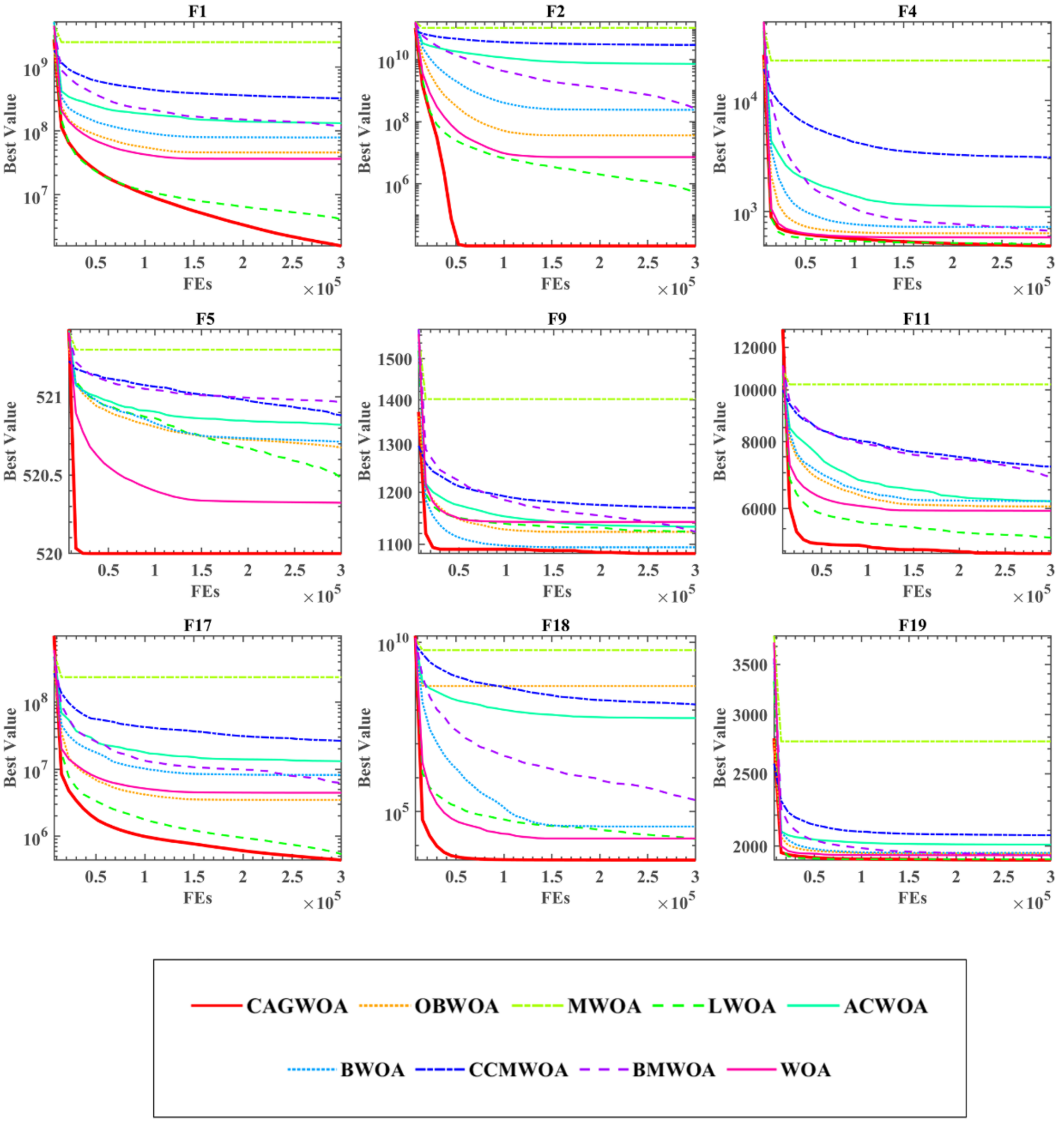
Convergence curve of CAGWOA compared with WOA variants.

From the convergence curves in Fig. 9, CAGWOA has the fastest convergence speed and the best convergence results on F1, F2, F4, F5, F9, F11, F17, F18, and F19 of IEEE CEC 2014. It can be seen from F2, F4, F5, F9, F11, F18, and F19 that all other algorithms fall into local optimum at the late stage of iteration, while CAGWOA relies on its strong exploration ability to obtain the optimal solution at the initial stage of iteration. The convergence curves of F1 and F17 show that CAGWOA maintains a stable exploitation ability at the late iteration when other algorithms gradually fall into local optimum. The convergence result of LWOA is second only to CAGWOA, but CAGWOA outperforms LWOA in terms of convergence speed and results obtained. in summary, CAGWOA outperforms other WOA improvement algorithms in terms of both the exploration ability in the early stage of evaluation and the exploration ability in the late stage.

## Segmentation experiments for COVID-19 X-ray image

To verify the segmentation effectiveness of CAGWOA, and the adaptability under different thresholds. In this section, CAGWOA was compared with six other segmentation algorithms, namely WOA, HHO, IWOA^115^, BLPSO^116^, CLPSO^117^, and SCADE^118^, in segmentation experiments using six X-ray images based on patients with COVID-19. The experiments were performed not only at levels 4, 6, and 8, which represent low threshold levels, but also at entropy levels 10, 12, and 14, which represent high threshold levels.

### Experimental setup and data set

**Figure 10:**
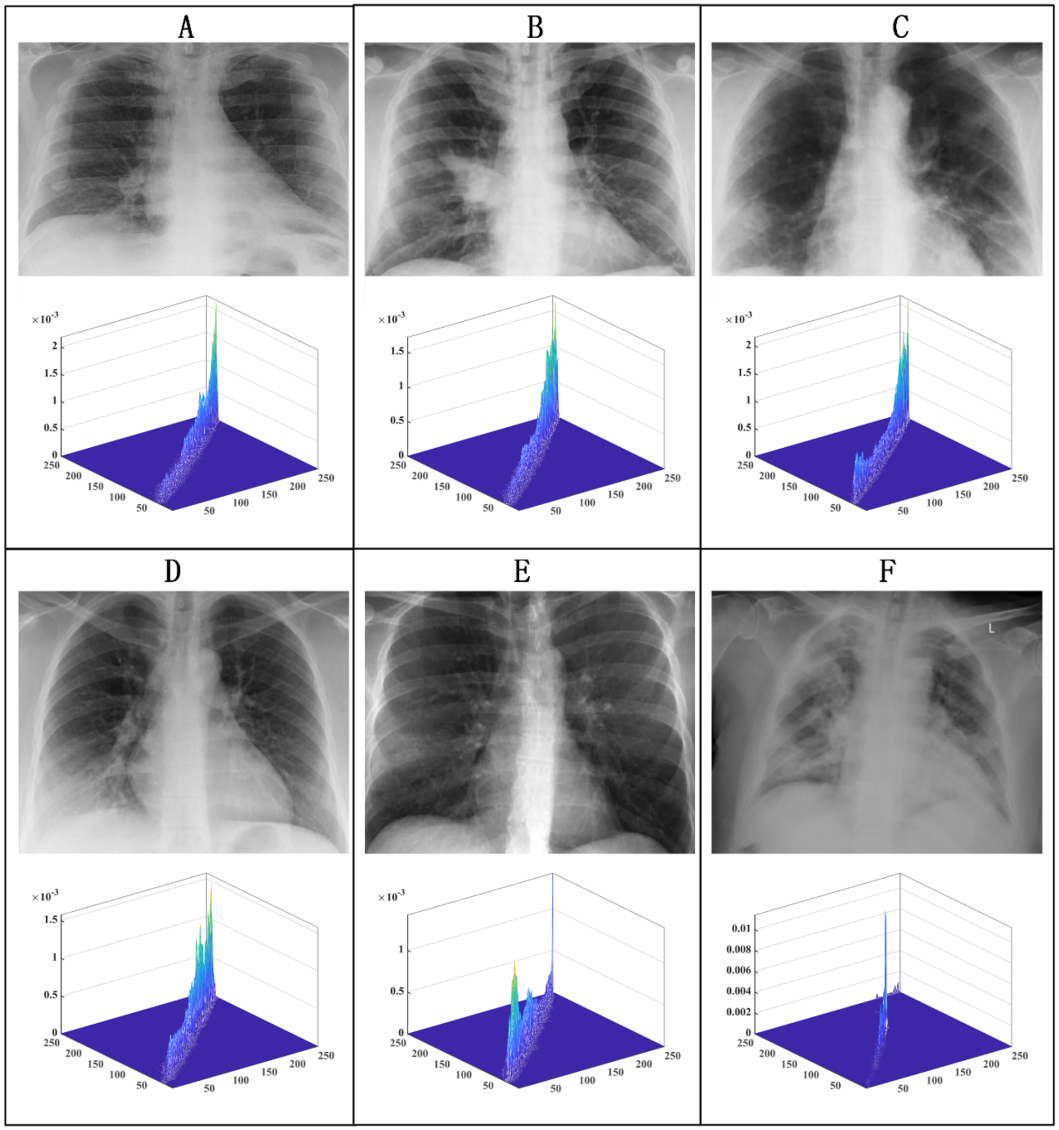
Original image and 2D distribution histogram.

The data used in the experiments were obtained from six lung X-ray images of COVID-19 patients from the public dataset collected by J.P. Cohen et al.^119^. The corresponding raw images and 2D distribution histograms for A, B, C, D, E, and F are shown in Fig. 10.

During the experiments conducted, all experiments were performed for 100 iterations to ensure the fairness and reliability of the experimental results, and the size of the segmented images was set to $512\times 512$. In the process of selecting the optimal segmentation threshold set by maximizing Kapur's entropy based on optimization algorithms. The algorithm’s population size was set to 20. To eliminate the randomness of the experiments, all experiments were run independently 30 times.

The selection of an appropriate threshold number holds paramount importance in image segmentation, directly influencing both result accuracy and computational efficiency. A threshold set too low may culminate in under-segmentation, whereas an overly high threshold may precipitate over-segmentation, leading to resource wastage. In the realm of medical image processing, meticulous experimentation and validation are typically undertaken to ascertain the optimal threshold number. In this study, the threshold numbers were judiciously selected based on general guidelines as referenced^78,120^. Specifically, lung X-ray images of COVID-19 patients were segmented using thresholds of 4, 6, and 8, denoting low threshold levels, as well as thresholds of 10, 12, and 14, representing high threshold levels. This approach provides a comprehensive exploration across varying threshold ranges, facilitating a nuanced understanding of segmentation outcomes.

### Performance evaluation parameters

In this paper, to comprehensively analyze the results of lung X-ray image segmentation of novel coronavirus patients by BDFXHGS, the experimental results were evaluated on three criteria: peak signal-to-noise ratio (PSNR)^121^, structural similarity index (SSIM)^122^, and feature similarity index (FSIM)^123^. Among them, PSNR is an image quality index that combines noise and accuracy, and a higher value indicates a higher quality for segmentation. SSIM describes the similarity before and after segmentation. The higher value of the segmentation result indicates that the difference after segmentation is smaller and the segmentation result is closer. FSIM evaluates the feature similarity between the segmented image and the original image, and a higher value indicates that the segmentation result is better at preserving the features of the original image. Table 9, provides more details on these three criteria.

**Table 9:** Introduction of image segmentation performance evaluation parameters.

Indicators	Formulation	Remark
PSNR	$PSNR=20\cdot {\text{log}}_{10}\left[\frac{255}{\sqrt{\frac{\sum_{i=0}^{M-1} \sum_{j=0}^{N-1} {\left({I}_{ij}-Se{g}_{ij}\right)}^{2}}{M\times N}}}\right]$	Compare the split image to the original image and evaluate the differences
SSIM	$\text{SSIM}=\frac{\left(2{\mu }_{I}{\mu }_{\text{Seg }}+{c}_{1}\right)\left(2{\sigma }_{I,\text{ Seg }}+{c}_{2}\right)}{\left({\mu }_{I}^{2}+{\mu }_{Seg}^{2}+{c}_{1}\right)\left({\sigma }_{I}^{2}+{\sigma }_{Seg}^{2}+{c}_{2}\right)}$	A comparison with the segmented, non-distorted, uncompressed image is performed to determine differences and similarities
FSIM	$FSIM=\frac{\sum_{I\in\Omega } {S}_{L}(X)P{C}_{m}(X)}{\sum_{I\in\Omega } P{C}_{m}(X)}$	Outlining the quality score which demonstrates the importance of a local structure

### Experimental analysis at low threshold levels

In this section, we focus on evaluating the image segmentation effect of CAGWOA at low threshold levels. Based on the image segmentation algorithms in Sect. "Theoretical backgrounds", a total of seven algorithms, CAGWOA and WOA, HHO, IWOA, BLPSO, CLPSO, and SCADE, using thresholds 4, 6, and 8, respectively, were used to perform segmentation experiments on the six images in Fig. 6.

**Figure 11:**
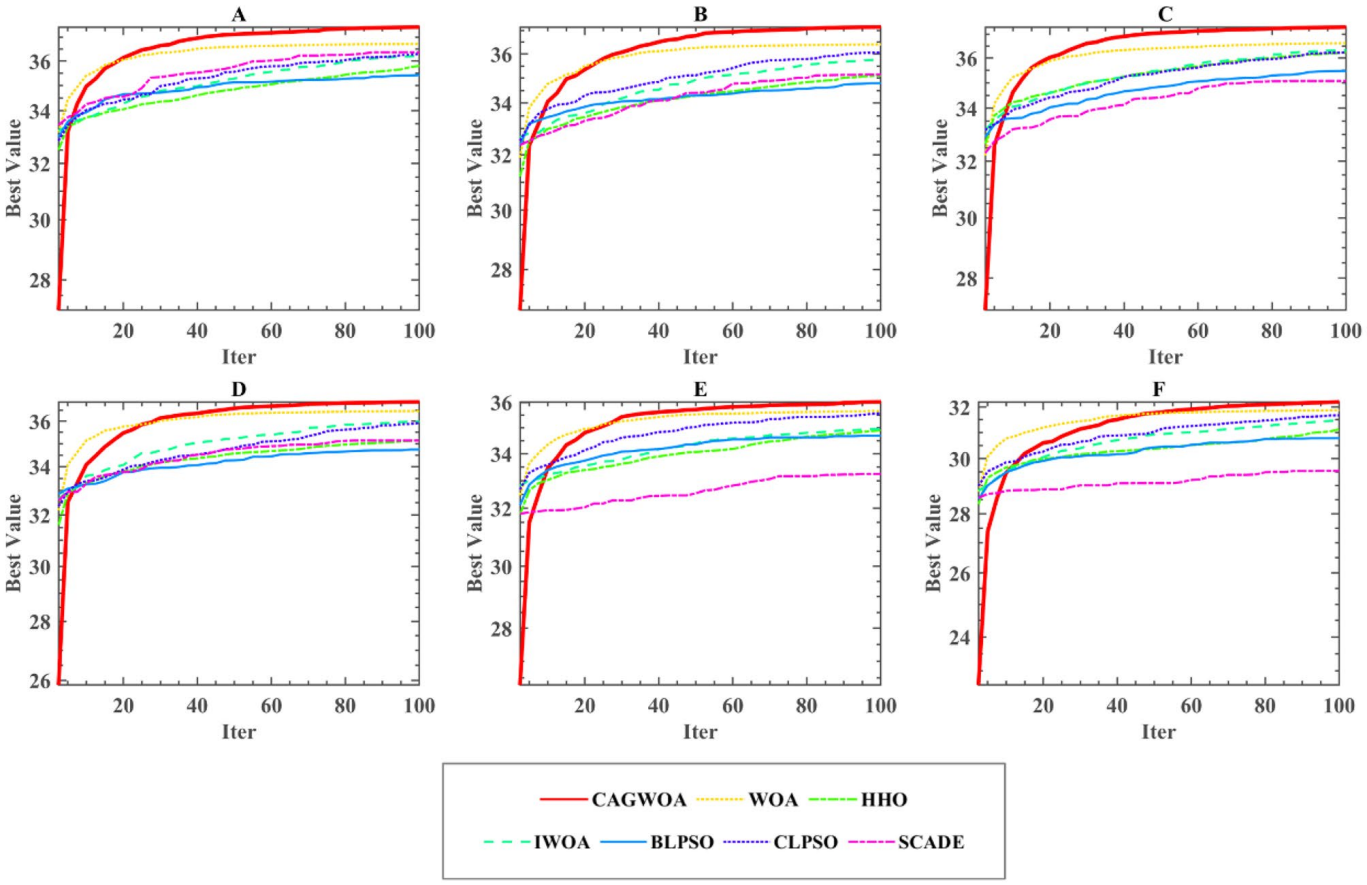
Convergence curve of segmentation of image A under threshold 4.

To further evaluate the experimental results and comprehensively assess the algorithm performance, the segmentation results were evaluated using PSNR, SSIM, and FSIM, and the results were further examined using the mean and variance and Wilcoxon signed rank tests. The evaluation results of PSNR, SSIM, and FSIM for six images at low threshold levels of 4–6 are given in Table 10 and Tables A.8, A.9 in the Appendix. From the experimental results, CAGWOA ranks first in both PSNR, SSIM and FSIM. This indicates that CAGWOA outperforms the other algorithms in terms of comprehensive performance of image segmentation at low threshold levels on 4, 6, and 8 thresholds. In addition, Fig. 11 shows the convergence curves of the segmentation experiments of CAGWOA and other compared algorithms for image A at a threshold value of 4. From the curves, CAGWOA converges the fastest and is less likely to fall into local optima during the convergence process, resulting in a greater 2D Kapur’s entropy.

**Table 10:** Results of PSNR analysis at low threshold levels.

Threshold		CAGWOA	WOA	HHO	IWOA	BLPSO	CLPSO	SCADE
4	+ /–/ =	~	2/0/4	2/0/4	1/0/5	4/0/2	1/0/5	5/0/1
	Mean	1.5000	4.3333	4.0000	2.5000	5.5000	4.0000	6.1667
	Rank	1	5	3	2	6	3	7
6	+ /–/ =	~	1/0/5	3/0/3	2/0/4	3/0/3	3/0/3	6/0/0
	Mean	1.3333	2.3333	4.8333	4.3333	5.1667	3.1667	6.8333
	Rank	1	2	5	4	6	3	7
8	+ /–/ =	~	2/0/4	5/0/1	4/0/2	2/0/4	4/0/2	6/0/0
	Mean	1.6667	2.1667	5.5000	4.0000	3.8333	4.0000	6.8333
	Rank	1	2	6	4	3	4	7

**Figure 12:**
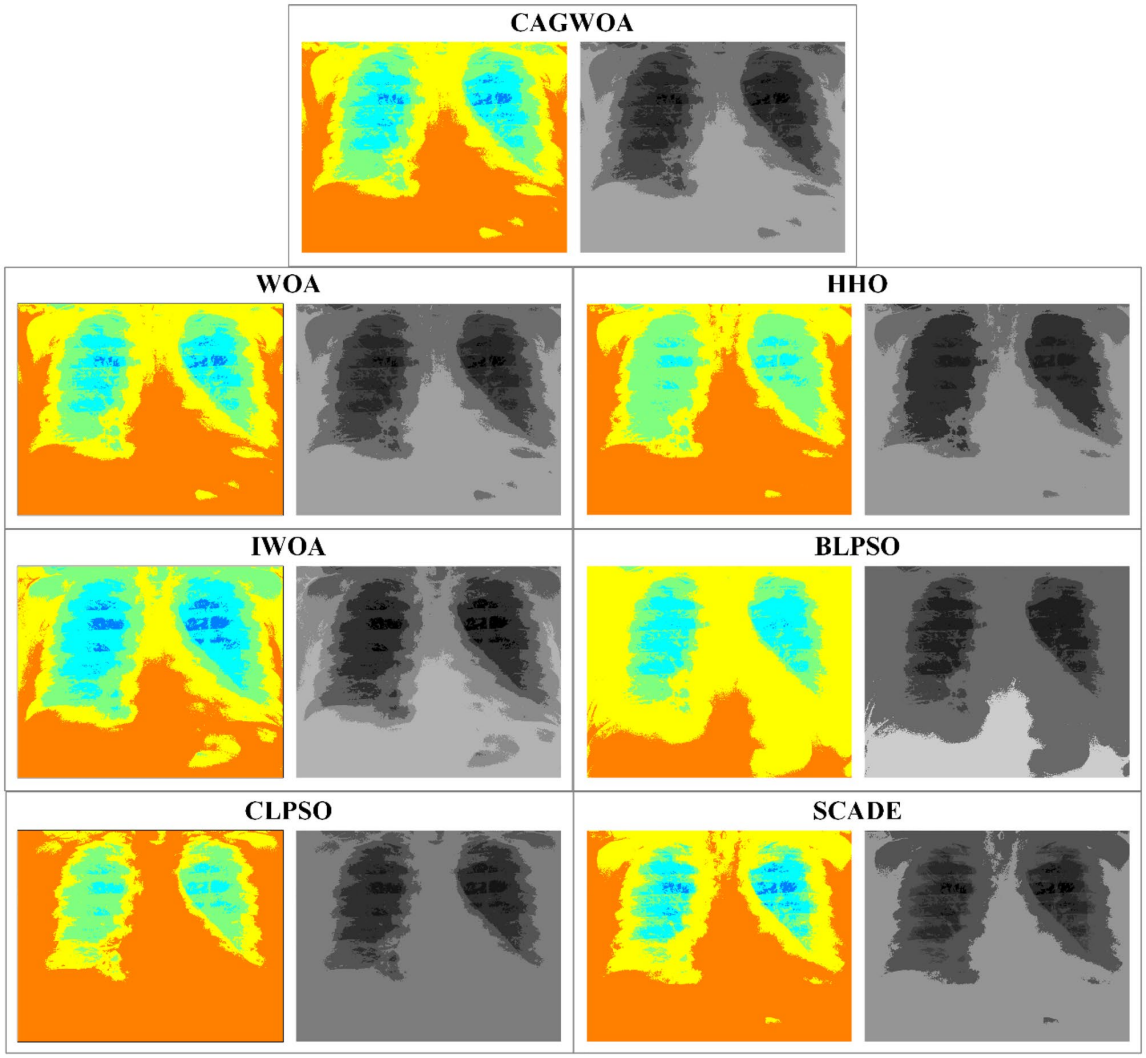
Comparison of segmentation results of image A under threshold 4.

Figure 12 shows the segmentation results and color mapping results of all segmentation algorithms for COVID-19 patient chest radiograph A under threshold 4. From the results, it can be seen that CAGWOA has relatively clear boundaries and better detail retention compared to the segmentation results of other comparison algorithms. According to the comparative analysis of the above experimental results, the CAGWOA outperforms other algorithms at low threshold levels.

### Experimental analysis at high threshold levels

To further verify the performance of CAGWOA in the field of image segmentation, six images, A, B, C, D, E, and F, were subjected to experiments at threshold levels 10, 12, and 14 representing high thresholding. Table 11 and Tables A.10 and A.11 in the Appendix give the results of the WSRT of the PSNR, SSIM, and FSIM means for the experimental results of CAGWOA and other algorithms at the high threshold level, respectively. From the experimental results, CAGWOA ranked first in all thresholds. And the segmentation results for the six images A, B, C, D, E and F are better or equal to other algorithms. This indicates that CAGWOA has stronger performance compared to other algorithms at high threshold levels.

**Table 11:** Results of PSNR analysis at high threshold levels.

Threshold		CAGWOA	WOA	HHO	IWOA	BLPSO	CLPSO	SCADE
10	+ /–/ =	~	2/0/4	5/0/1	5/0/1	3/0/3	5/0/1	6/0/0
	Mean	1.3333	2.0000	4.8333	5.5000	3.1667	4.1667	7.0000
	Rank	1	2	5	6	3	4	7
12	+ /–/ =	~	1/0/5	5/0/1	6/0/0	5/0/1	3/0/3	6/0/0
	Mean	1.3333	1.6667	4.1667	6.0000	4.3333	3.5000	7.0000
	Rank	1	2	4	6	5	3	7
14	+ /–/ =	~	0/0/6	5/0/1	6/0/0	4/0/2	5/0/1	6/0/0
	Mean	1.3333	1.6667	4.5000	5.1667	4.0000	4.5000	6.8333
	Rank	1	2	4	6	3	4	7

**Figure 13:**
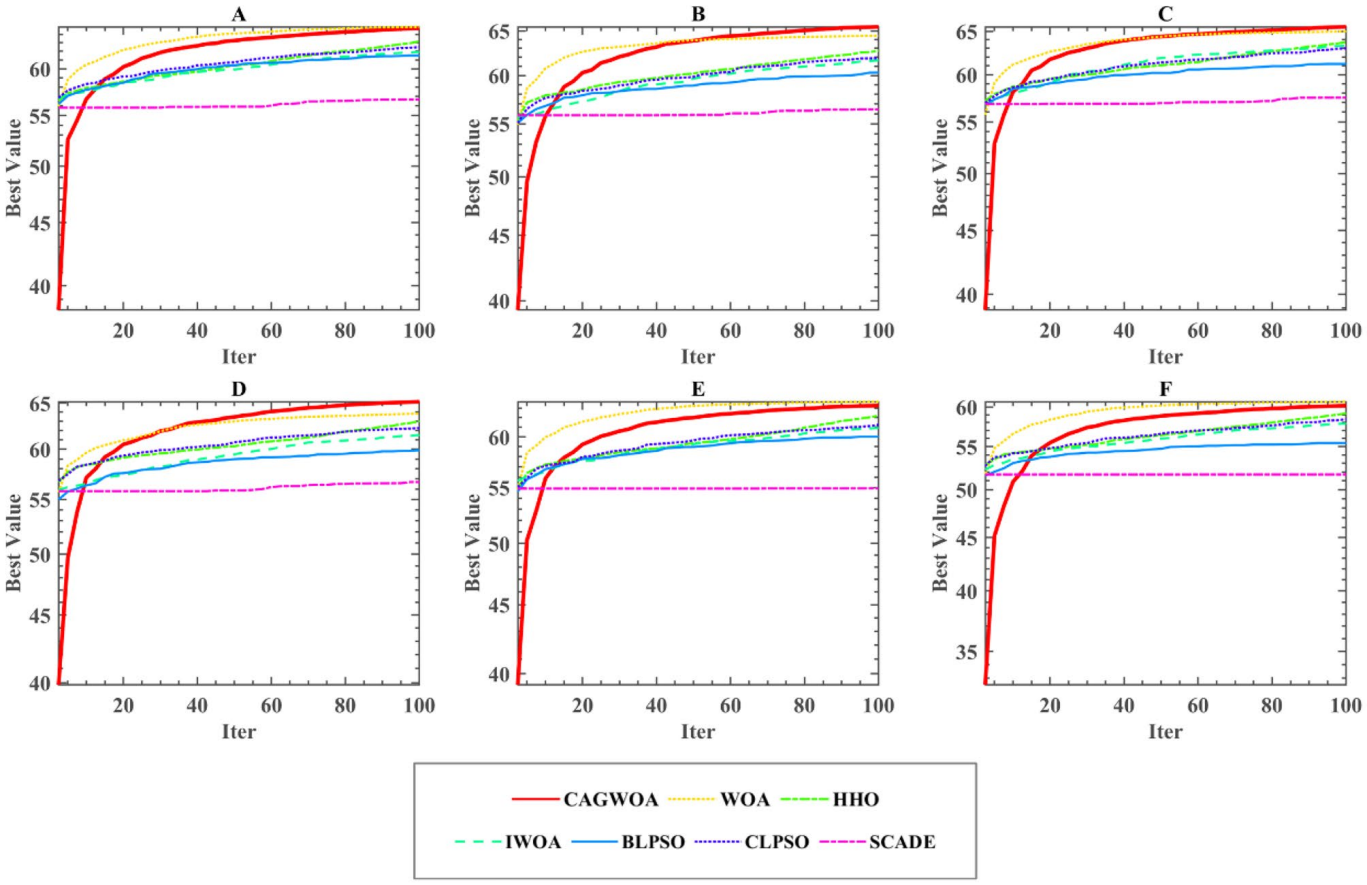
Convergence curve of segmentation of image A under threshold 14.

Figure 13 shows the convergence curves of the segmentation experiments of CAGWOA and the other compared algorithms for image A at a threshold value of 14. From the curves, CAGWOA has the ability to converge quickly at the beginning of the convergence and maintains a stable exploitation ability during the convergence process, thus obtaining a larger 2D Kapur’s entropy and better experimental results.

**Figure 14:**
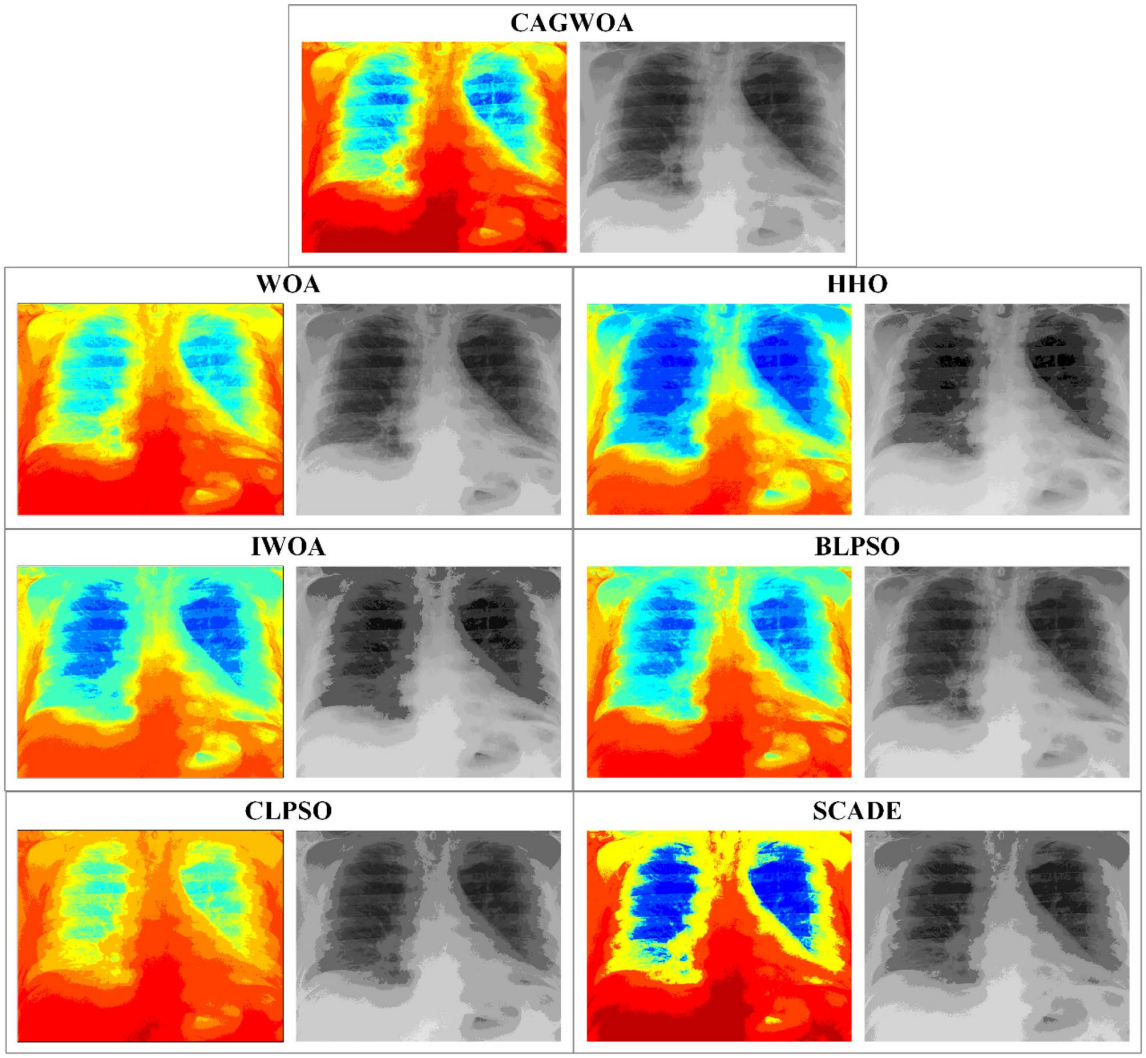
Comparison of segmentation results of image A under threshold 14.

From the segmentation results for image A under threshold 14 shown in Fig. 14, CAGWOA is better than other algorithms in preserving local features of the image, retains more image detail and has higher accuracy. According to the analysis of the above experimental results, CAGWOA shows good adaptability to image segmentation with high thresholds, and the segmentation performance is better than other comparative algorithms.

### Analysis of experimental results of image segmentation

**Figure 15:**
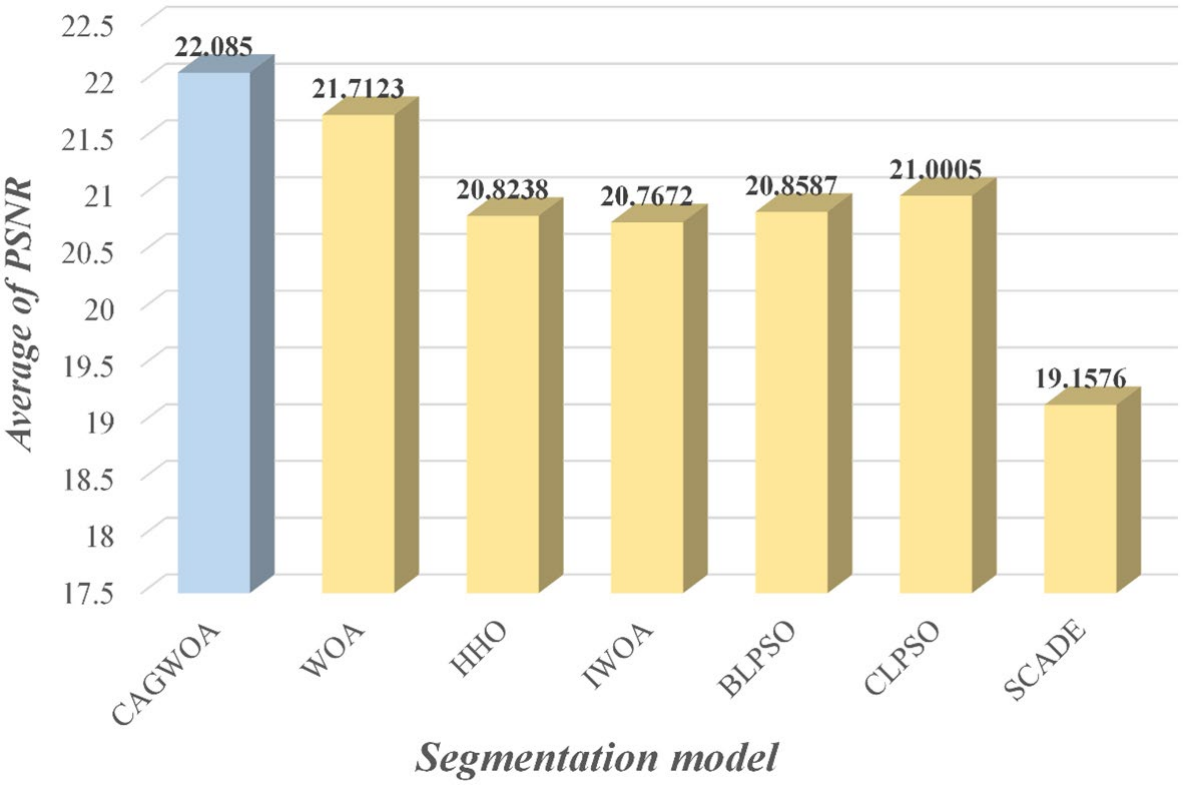
Average PSNR assessment results at all threshold levels.

**Figure 16:**
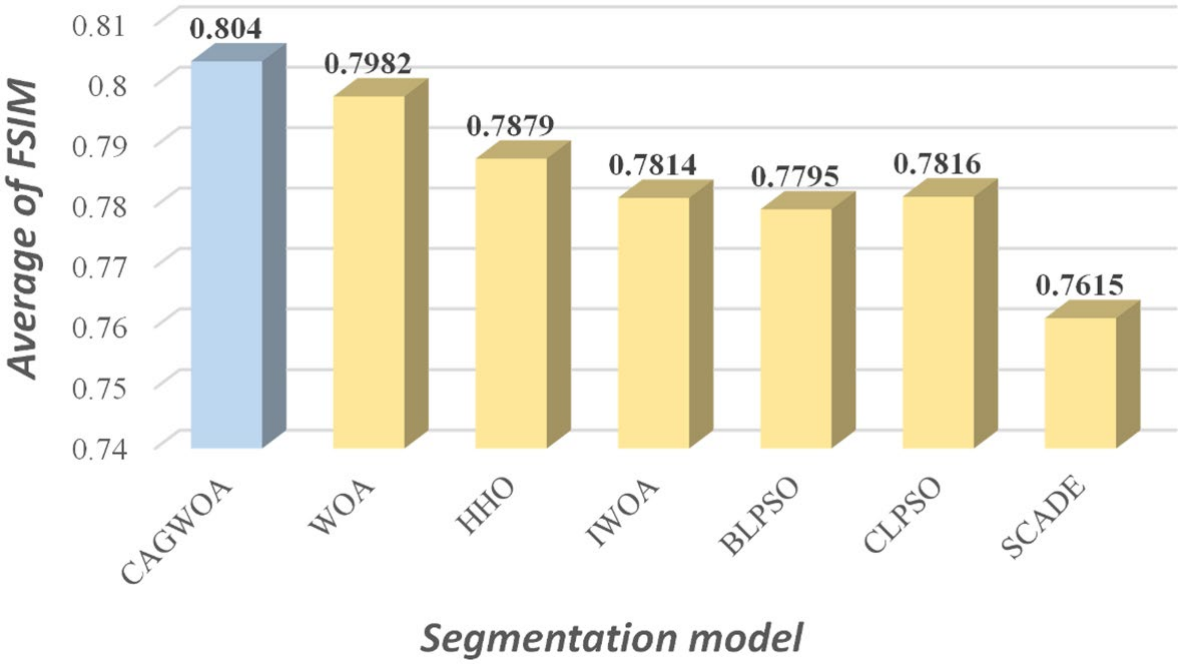
Average FSIM assessment results at all threshold levels.

**Figure 17:**
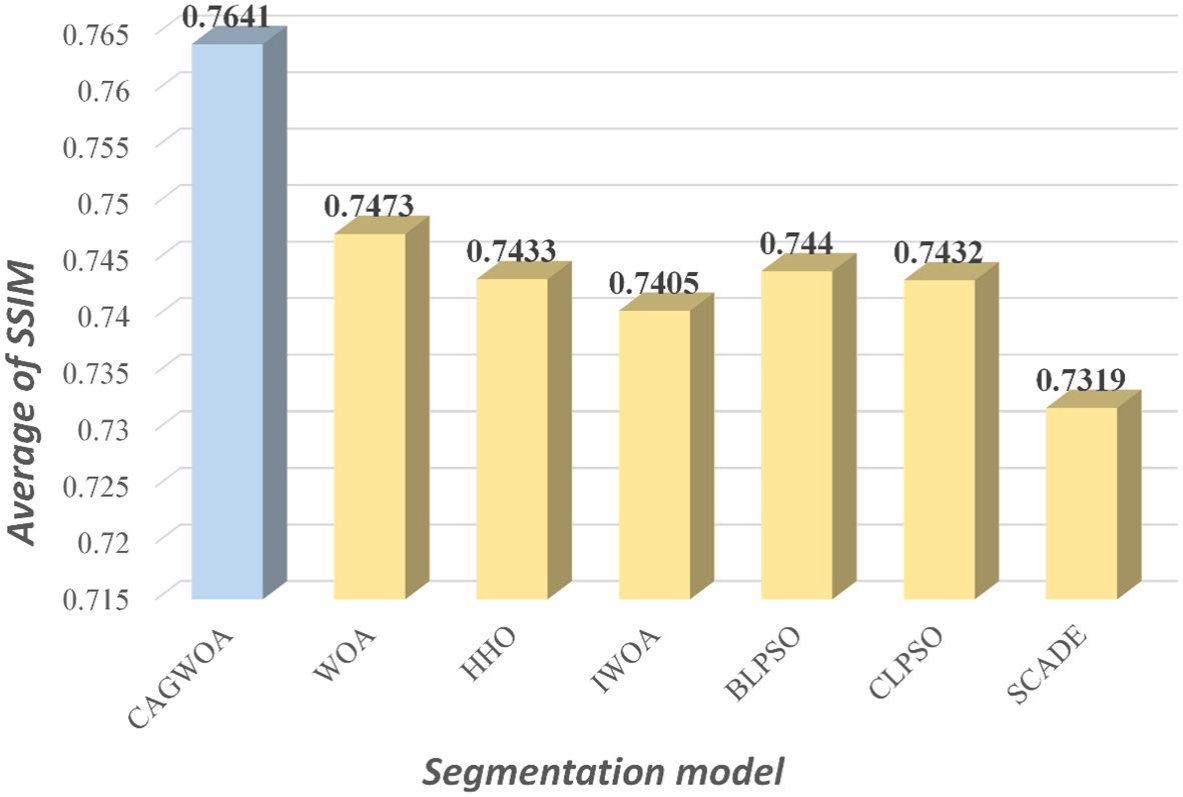
Average SSIM assessment results at all threshold levels.

Figures 15, 16 and 17 show the average values of the CAGWOA and other comparison algorithms for the three evaluation criteria of 4, 6, 8, 10, 12, and 14 threshold levels on pictures A, B, C, D, E, and F. The model achieves optimality for all three criteria. Therefore, the CAGWOA has stable results with different threshold levels and has excellent performance.

The analysis of the experimental results concludes that CAGWOA shows better thresholding adaptability and stability in image segmentation, finding better thresholds at both low and high threshold levels. Combining the evaluation results of PSNR, SSIM and FSIM, when CAGWOA is applied to image segmentation experiments, it can clearly delineate the boundaries of features while retaining more local details, making the segmentation results closer to the original image.

## Conclusion and future works

To improve the efficacy of lung segmentation on X-ray images of novel coronavirus patients, in this paper, an image segmentation model centered on the improved whale optimization algorithm (CAGWOA) is introduced. The proposed algorithm is based on a COS sampling initialization strategy, an adaptive global search strategy, and an all-dimensional neighborhood mechanism. Among them, the COS sampling initialization strategy is presented to take the role of the original method of random initialization, which enhances the performance of WOA on complex multimodal and mixed functions. The all-dimensional neighborhood mechanism enhances the ability of WOA to exploit optimal individuals. And in order to prevent WOA from falling into local optimum, the global search method is utilized to improve its ability to search globally. Benchmark function experimental results show that CAGWOA has faster convergence speed, higher convergence accuracy, and a stronger ability to avoid jumping out of the local optimum. CAGWOA and a series of comparison algorithms are applied to an image segmentation model based on 2D Kapur’s entropy and non-local mean two-dimensional distribution histograms. COVID-19 multi-threshold image segmentation experiments show that the multi-threshold image segmentation model based on CAGWOA exhibits some adaptability with better quality segmentation results under different threshold levels.

This paper aims to improve medical diagnosis by improving the efficiency and effectiveness of lung X-ray image segmentation in patients with novel coronavirus pneumonia. Since image segmentation is only a basic task for medical diagnosis, we will continue our efforts in feature extraction, selection, and classification of X-ray lung images of patients with novel coronavirus infections to further improve the accuracy and efficiency of medical diagnosis. These beneficial efforts will help to better understand and respond to diseases such as novel coronavirus pneumonia and provide more comprehensive information to improve treatment options for patients. In addition, the image segmentation model delineated in this study, anchored by CAGWOA, is poised to be extended to medical image segmentation across a broader spectrum of diseases. Such an advancement holds promise for making substantial strides in the medical arena. Concurrently, the optimization prowess inherent to CAGWOA, characterized by its efficiency as an optimization algorithm, will be harnessed in diverse applications. Anticipated applications encompass challenges posed by expansive datasets and real-time applications. Moreover, CAGWOA is anticipated to play an instrumental role in diverse domains, including scheduling quandaries, feature selection, and engineering optimization challenges, further amplifying its potential impact across various scientific and technological disciplines.

### Supplementary Information

Supplementary Tables.

## Data Availability

The data involved in this study are all public data, which can be downloaded through public channels: https://github.com/ZhenWangjyqj/Data-availability-statement-of-CAGWOA.

## References

[1] Oliva A, Torralba A (2006). Building the gist of a scene: The role of global image features in recognition. Prog. Brain Res..

[2] Aggarwal P (2011). Role of segmentation in medical imaging: A comparative study. Int. J. Comput. Appl..

[3] Cheng H-D (2001). Color image segmentation: Advances and prospects. Pattern Recognit..

[4] Elizabeth DS (2012). A novel segmentation approach for improving diagnostic accuracy of CAD systems for detecting lung cancer from chest computed tomography images. J. Data Inf. Qual. (JDIQ).

[5] Li X (2018). H-DenseUNet: hybrid densely connected UNet for liver and tumor segmentation from CT volumes. IEEE Trans. Med. Imaging.

[6] Yan, Q., et al. *COVID-19 chest CT image segmentation--a deep convolutional neural network solution.* Preprint at https://arXiv.org/arXiv:2004.10987 (2020).

[7] Zhu, L., et al. *An effective interactive medical image segmentation method using fast growcut*. in *MICCAI workshop on interactive medical image computing*. 2014.

[8] Shah FM (2021). A comprehensive survey of covid-19 detection using medical images. SN Comput. Sci..

[9] Abumalloh RA (2022). Medical image processing and COVID-19: A literature review and bibliometric analysis. J. Infect. Public Health.

[10] Kumar A, Gupta PK, Srivastava A (2020). A review of modern technologies for tackling COVID-19 pandemic. Diabetes Metab. Syndr. Clin. Res. Rev..

[11] Zhang X, Dahu W (2019). Application of artificial intelligence algorithms in image processing. J. Visual Commun. Image Represent..

[12] Robertson S (2018). Digital image analysis in breast pathology—From image processing techniques to artificial intelligence. Transl. Res..

[13] Ye Z (2012). Image segmentation using thresholding and swarm intelligence. J. Softw..

[14] Storn R, Price K (1997). Differential evolution-a simple and efficient heuristic for global optimization over continuous spaces. J. Glob. Optim..

[15] Dorigo M, Birattari M, Stutzle T (2006). Ant colony optimization. IEEE Comput. Intell. Mag..

[16] Bayraktar, Z., M. Komurcu, and D.H. Werner. *Wind Driven Optimization (WDO): A novel nature-inspired optimization algorithm and its application to electromagnetics*. In *2010 IEEE antennas and propagation society international symposium* (IEEE, 2010).

[17] Mirjalili S (2015). Moth-flame optimization algorithm: A novel nature-inspired heuristic paradigm. Knowl.-Based Syst..

[18] Mirjalili S (2016). SCA: A sine cosine algorithm for solving optimization problems. Knowl.-Based Syst..

[19] Tu J (2021). The colony predation algorithm. J. Bionic Eng..

[20] Yang, X.-S., *A new metaheuristic bat-inspired algorithm.* Nature inspired cooperative strategies for optimization (NICSO 2010), p. 65–74 (2010).

[21] Yang Y (2021). Hunger games search: Visions, conception, implementation, deep analysis, perspectives, and towards performance shifts. Expert Syst. Appl..

[22] Heidari AA (2019). Harris hawks optimization: Algorithm and applications. Future Gen. Comput. Syst..

[23] Marini F, Walczak B (2015). Particle swarm optimization (PSO). A tutorial. Chemomet. Intell. Lab. Syst..

[24] Yang X-S, He X (2013). Firefly algorithm: Recent advances and applications. Int. J. Swarm Intell..

[25] Mirjalili S, Mirjalili SM, Lewis A (2014). Grey wolf optimizer. Adv. Eng. Softw..

[26] Ahmadianfar I (2021). RUN beyond the metaphor: An efficient optimization algorithm based on Runge Kutta method. Expert Syst. Appl..

[27] Mirjalili S, Lewis A (2016). The whale optimization algorithm. Adv. Eng. Softw..

[28] Jiang R (2020). An improved whale optimization algorithm with armed force program and strategic adjustment. Appl. Math. Model..

[29] Chakraborty S (2021). A novel enhanced whale optimization algorithm for global optimization. Comput. Ind. Eng..

[30] Zhang J, Hong L, Liu Q (2020). An improved whale optimization algorithm for the traveling salesman problem. Symmetry.

[31] Huang M, Cheng X, Lei Y (2021). Structural damage identification based on substructure method and improved whale optimization algorithm. J. Civil Struct. Health Monit..

[32] Qiao W (2020). Short-term natural gas consumption prediction based on Volterra adaptive filter and improved whale optimization algorithm. Eng. Appl. Artif. Intell..

[33] Pandey AC, Tikkiwal VA (2021). Stance detection using improved whale optimization algorithm. Complex Intell. Syst..

[34] Chen H (2020). An efficient double adaptive random spare reinforced whale optimization algorithm. Expert Syst. Appl..

[35] Jia L, Li K, Shi X (2021). Cloud computing task scheduling model based on improved whale optimization algorithm. Wirel. Commun. Mobile Comput..

[36] Wolpert DH, Macready WG (1997). No free lunch theorems for optimization. IEEE Trans. Evolut. Comput..

[37] Imran M, Hashim R, Abd Khalid NE (2013). An overview of particle swarm optimization variants. Procedia Eng..

[38] Beheshti Z, Shamsuddin SMH (2013). A review of population-based meta-heuristic algorithms. Int. J. Adv. Soft Comput. Appl.

[39] Li Q, Liu S-Y, Yang X-S (2020). Influence of initialization on the performance of metaheuristic optimizers. Appl. Soft Comput..

[40] Hassanzadeh MR, Keynia F (2021). An overview of the concepts, classifications, and methods of population initialization in metaheuristic algorithms. J. Adv. Comput. Eng. Technol..

[41] Sarhani M, Voß S, Jovanovic R (2023). Initialization of metaheuristics: Comprehensive review, critical analysis, and research directions. Int. Trans. Oper. Res..

[42] Shields MD, Zhang J (2016). The generalization of Latin hypercube sampling. Reliab. Eng. Syst. Saf..

[43] Deutsch JL, Deutsch CV (2012). Latin hypercube sampling with multidimensional uniformity. J. Stat. Plan. Inference.

[44] Mousavirad, S.J., et al. *Tackling deceptive optimization problems using opposition-based DE with center-based latin hypercube initialization*. In *14th International Conference on Computer Science and Education (ICCSE)* (Ontario Tech Univ, 2019).

[45] Liang JJ, Qu BY, Suganthan PN (2013). Problem definitions and evaluation criteria for the CEC 2014 special session and competition on single objective real-parameter numerical optimization. Comput. Intell. Lab..

[46] Price, K., et al. *Problem definitions and evaluation criteria for the 100-digit challenge special session and competition on single objective numerical optimization*, In *Technical report*, Nanyang Technological University Singapore (2018).

[47] Kumar, A. et al. *Problem definitions and evaluation criteria for the CEC 2022 special session and competition on single objective bound constrained numerical optimization* (2022).

[48] Guo Z (2019). Deep learning-based image segmentation on multimodal medical imaging. IEEE Trans. Radiat. Plasma Med. Sci..

[49] Dhanachandra N, Chanu YJ (2017). A survey on image segmentation methods using clustering techniques. Eur. J. Eng. Technol. Res..

[50] Raju PDR, Neelima G (2012). Image segmentation by using histogram thresholding. Int. J. Comput. Sci. Eng. Technol..

[51] Sun R (2022). Survey of image edge detection. Front. Signal Process..

[52] Kohler R (1981). A segmentation system based on thresholding. Comput. Graph. Image Process..

[53] Khishe M (2023). An automatic COVID-19 diagnosis from chest X-ray images using a deep trigonometric convolutional neural network. Imaging Sci. J..

[54] Wang X (2022). Pulmonary diffuse airspace opacities diagnosis from chest X-ray images using deep convolutional neural networks fine-tuned by whale optimizer. Wirel. Pers. Commun..

[55] Liu H (2023). A few-shot learning approach for covid-19 diagnosis using Quasi-configured topological spaces. J. Artif. Intell. Soft Comput. Res..

[56] Debelee TG (2019). Evaluation of modified adaptive k-means segmentation algorithm. Comput. Visual Media.

[57] Wang G (2018). Interactive medical image segmentation using deep learning with image-specific fine tuning. IEEE Trans. Med. Imaging.

[58] Işın A, Direkoğlu C, Şah M (2016). Review of MRI-based brain tumor image segmentation using deep learning methods. Procedia Comput. Sci..

[59] Isensee F (2021). nnU-Net: A self-configuring method for deep learning-based biomedical image segmentation. Nat. Methods.

[60] Haque IRI, Neubert J (2020). Deep learning approaches to biomedical image segmentation. Inform. Med. Unlocked.

[61] Xu B (2022). COVID-19 diagnosis using chest CT scans and deep convolutional neural networks evolved by IP-based sine-cosine algorithm. Med. Biol. Eng. Comput..

[62] Cai C (2023). Improved deep convolutional neural networks using chimp optimization algorithm for Covid19 diagnosis from the X-ray images. Expert Syst. Appl..

[63] Saffari A (2022). DCNN-fuzzyWOA: Artificial intelligence solution for automatic detection of covid-19 using X-ray images. Comput. Intell. Neurosci..

[64] Hu T (2021). Real-time COVID-19 diagnosis from X-Ray images using deep CNN and extreme learning machines stabilized by chimp optimization algorithm. Biomed. Signal Process. Control.

[65] Wei X (2019). Defect detection of pantograph slide based on deep learning and image processing technology. IEEE Trans. Intell. Transport. Syst..

[66] Liu X (2021). A review of deep-learning-based medical image segmentation methods. Sustainability.

[67] Lai C-C, Chang C-Y (2009). A hierarchical evolutionary algorithm for automatic medical image segmentation. Expert Syst. Appl..

[68] Abdul-Nasir AS, Mashor MY, Mohamed Z (2013). Colour image segmentation approach for detection of malaria parasites using various colour models and k-means clustering. WSEAS Trans. Biol. Biomed.

[69] Mignotte M (2008). Segmentation by fusion of histogram-based $ k $-means clusters in different color spaces. IEEE Trans. Image Process..

[70] Juang L-H, Wu M-N (2010). MRI brain lesion image detection based on color-converted K-means clustering segmentation. Measurement.

[71] Kaur D, Kaur Y (2014). Various image segmentation techniques: A review. Int. J. Comput. Sci. Mobile Comput..

[72] Shan P (2018). Image segmentation method based on K-mean algorithm. EURASIP J. Image Video Process..

[73] Agarwal M, Mahajan R (2018). Medical image contrast enhancement using range limited weighted histogram equalization. Procedia Comput. Sci..

[74] Zhang X (2012). Medical image segmentation using improved FCM. Sci. China Inf. Sci..

[75] Bonnet N, Cutrona J, Herbin M (2002). A ‘no-threshold’histogram-based image segmentation method. Pattern Recognit..

[76] Sezan MI (1990). A peak detection algorithm and its application to histogram-based image data reduction. Comput. Vis. Graph. Image process..

[77] Ni K (2009). Local histogram based segmentation using the Wasserstein distance. Int. J. Comput. Vis..

[78] Bhargavi K, Jyothi S (2014). A survey on threshold based segmentation technique in image processing. Int. J. Innov. Res. Dev..

[79] Lalitha M, Kiruthiga M, Loganathan C (2013). A survey on image segmentation through clustering algorithm. Int. J. Sci. Res..

[80] Boskovitz V, Guterman H (2002). An adaptive neuro-fuzzy system for automatic image segmentation and edge detection. IEEE Trans. Fuzzy Syst..

[81] Savant S (2014). A review on edge detection techniques for image segmentation. Int. J. Comput. Sci. Inf. Technol.

[82] Bellon OR, Silva L (2002). New improvements to range image segmentation by edge detection. IEEE Signal Process. Lett..

[83] Meftah B, Lezoray O, Benyettou A (2010). Segmentation and edge detection based on spiking neural network model. Neural Process. Lett..

[84] Singleton HR, Pohost GM (1997). Automatic cardiac MR image segmentation using edge detection by tissue classification in pixel neighborhoods. Magn. Reson. Med..

[85] Muthukrishnan R, Radha M (2011). Edge detection techniques for image segmentation. Int. J. Comput. Sci. Inf. Technol..

[86] Kushwah A (2017). A review: Comparative study of edge detection techniques. Int. J. Adv. Res. Comput. Sci..

[87] Chen YB, Chen OT (2009). Image segmentation method using thresholds automatically determined from picture contents. Eurasip J. Image Video Process..

[88] Waarsing JH, Day JS, Weinans H (2004). An improved segmentation method for in vivo μCT imaging. J. Bone Miner. Res..

[89] Zhao D (2021). Chaotic random spare ant colony optimization for multi-threshold image segmentation of 2D Kapur entropy. Knowl.-Based Syst..

[90] Al-Amri, S.S. and Kalyankar, N.V. *Image segmentation by using threshold techniques.* Preprint at https://arXiv.org//arXiv:1005.4020 (2010).

[91] Abdel-Basset M, Chang V, Mohamed R (2021). A novel equilibrium optimization algorithm for multi-thresholding image segmentation problems. Neural Comput. Appl..

[92] Buades, A., B. Coll, and J. Morel. *A non-local algorithm for image denoising*. In *2005 IEEE Computer Society Conference on Computer Vision and Pattern Recognition (CVPR'05)* (2005).

[93] Kollem S, Reddy KRL, Rao DS (2019). A review of image denoising and segmentation methods based on medical images. Int. J. Mach. Learn. Comput..

[94] Kapur JN (1985). A new method for gray-level picture thresholding using the entropy of the histogram. Comput. Vis. Graph. Image Process..

[95] Stein M (1987). Large sample properties of simulations using Latin hypercube sampling. Technometrics.

[96] Iman RL (2008). Latin Hypercube Sampling.

[97] Sun W (2017). All-dimension neighborhood based particle swarm optimization with randomly selected neighbors. Inf. Sci..

[98] García S (2010). Advanced nonparametric tests for multiple comparisons in the design of experiments in computational intelligence and data mining: Experimental analysis of power. Inf. Sci..

[99] Derrac J (2011). A practical tutorial on the use of nonparametric statistical tests as a methodology for comparing evolutionary and swarm intelligence algorithms. Swarm Evolut. Comput..

[100] Issa M (2018). ASCA-PSO: Adaptive sine cosine optimization algorithm integrated with particle swarm for pairwise local sequence alignment. Expert Syst. Appl..

[101] Zhang, H. and Hui, Q. *Cooperative bat searching algorithm: A combined perspective from multiagent coordination and swarm intelligence*. In *2017 13th IEEE Conference on Automation Science and Engineering (CASE)* (IEEE, 2017).

[102] Kumar N (2017). Single sensor-based MPPT of partially shaded PV system for battery charging by using cauchy and Gaussian sine cosine optimization. IEEE Trans. Energy Convers..

[103] Gai J (2020). An integrated method based on hybrid grey wolf optimizer improved variational mode decomposition and deep neural network for fault diagnosis of rolling bearing. Measurement.

[104] Sun K (2021). Scheduling model of power system based on forecasting error of wind power plant output. IEEJ Trans. Electr. Electron. Eng..

[105] Hamad, Q.S., et al. *A Comparative Study of Sine Cosine Optimizer and Its Variants for Engineering Design Problems*. In *Proc. of the 11th International Conference on Robotics, Vision, Signal Processing and Power Applications: Enhancing Research and Innovation through the Fourth Industrial Revolution* (Springer, 2022).

[106] Ye F, Lou XY, Sun LF (2017). An improved chaotic fruit fly optimization based on a mutation strategy for simultaneous feature selection and parameter optimization for SVM and its applications. PLoS One.

[107] Wu Y (2020). Hybrid symbiotic differential evolution moth-flame optimization algorithm for estimating parameters of photovoltaic models. IEEE Access.

[108] Abd Elaziz M, Oliva D (2018). Parameter estimation of solar cells diode models by an improved opposition-based whale optimization algorithm. Energy Convers. Manag..

[109] Sun Y (2018). A modified whale optimization algorithm for large-scale global optimization problems. Expert Syst. Appl..

[110] Ling Y, Zhou Y, Luo Q (2017). Lévy flight trajectory-based whale optimization algorithm for global optimization. IEEE Access.

[111] Tang C (2022). A hybrid whale optimization algorithm with artificial bee colony. Soft Comput..

[112] Chen H (2019). A balanced whale optimization algorithm for constrained engineering design problems. Appl. Math. Model..

[113] Luo J (2019). Multi-strategy boosted mutative whale-inspired optimization approaches. Appl. Math. Model..

[114] Heidari AA (2020). An enhanced associative learning-based exploratory whale optimizer for global optimization. Neural Comput. Appl..

[115] Tubishat M (2019). Improved whale optimization algorithm for feature selection in Arabic sentiment analysis. Appl. Intell..

[116] Chen X (2017). Biogeography-based learning particle swarm optimization. Soft Comput..

[117] Liang JJ (2006). Comprehensive learning particle swarm optimizer for global optimization of multimodal functions. IEEE transactions on evolutionary computation.

[118] Nenavath H, Jatoth RK (2018). Hybridizing sine cosine algorithm with differential evolution for global optimization and object tracking. Appl. Soft Comput..

[119] Cohen, J.P., et al. *Covid-19 image data collection: Prospective predictions are the future.* https://github.com/ieee8023/covid-chestxray-dataset (2020).

[120] He H-J, Zheng C, Sun D-W (2016). Image segmentation techniques. Computer Vision Technology for Food Quality Evaluation.

[121] Huynh-Thu Q, Ghanbari M (2008). Scope of validity of PSNR in image/video quality assessment. Electron. Lett..

[122] Zhou W (2004). Image quality assessment: From error visibility to structural similarity. IEEE Trans. Image Process..

[123] Zhang L (2011). FSIM: A feature similarity index for image quality assessment. IEEE Trans. Image Process..

