# Supplementary material for: Improved Latin hypercube sampling initialization-based whale optimization algorithm for COVID-19 X-ray multi-threshold image segmentation
Source: Sci Rep. 2024 Jun 9;14:13239. doi: 10.1038/s41598-024-63739-9 (PMC11163015; doi:10.1038/s41598-024-63739-9)
Supplement: Supplementary file 1 — Supplementary Tables. [file 41598_2024_63739_MOESM1_ESM.docx]

**Appendix A**

**Table A.1** Introduction of the benchmark functions

IEEE CEC 2014 benchmark functions:

|  | No. | Functions | *Fi*=Fi(x*)* |
| --- | --- | --- | --- |
| Unimodal  Functions | 1 | Rotated High Conditioned Elliptic Function | 100 |
|  | 2 | Rotated Bent Cigar Function | 200 |
|  | 3 | Rotated Discus Function | 300 |
| Simple  Multimodal  Functions | 4 | Shifted and Rotated Rosenbrock’s Function | 400 |
|  | 5 | Shifted and Rotated Ackley’s Function | 500 |
|  | 6 | Shifted and Rotated Weierstrass Function | 600 |
|  | 7 | Shifted and Rotated Griewank’s Function | 700 |
|  | 8 | Shifted Rastrigin’s Function | 800 |
|  | 9 | Shifted and Rotated Rastrigin’s Function | 900 |
|  | 10 | Shifted Schwefel’s Function | 1000 |
|  | 11 | Shifted and Rotated Schwefel’s Function | 1100 |
|  | 12 | Shifted and Rotated Katsuura Function | 1200 |
|  | 13 | Shifted and Rotated HappyCat Function | 1300 |
|  | 14 | Shifted and Rotated HGBat Function | 1400 |
|  | 15 | Shifted and Rotated Expanded Griewank’s plus Rosenbrock’s Function | 1500 |
|  | 16 | Shifted and Rotated Expanded Scaffer’s F6 Function | 1600 |
| Hybrid  Functions | 17 | Hybrid Function 1 (N=3) | 1700 |
|  | 18 | Hybrid Function 2 (N=3) | 1800 |
|  | 19 | Hybrid Function 3 (N=4) | 1900 |
|  | 20 | Hybrid Function 4 (N=4) | 2000 |
|  | 21 | Hybrid Function 5 (N=5) | 2100 |
|  | 22 | Hybrid Function 6 (N=5) | 2200 |
| Composition  Functions | 23 | Composition Function 1 (N=5) | 2300 |
|  | 24 | Composition Function 2 (N=3) | 2400 |
|  | 25 | Composition Function 3 (N=3) | 2500 |
|  | 26 | Composition Function 4 (N=5) | 2600 |
|  | 27 | Composition Function 5 (N=5) | 2700 |
|  | 28 | Composition Function 6 (N=5) | 2800 |
|  | 29 | Composition Function 7 (N=3) | 2900 |
|  | 30 | Composition Function 8 (N=3) | 3000 |
| Search Range: [-100,100] | | | |

IEEE CEC 2019 benchmark functions:

| NO. | Functions | *Fi*=Fi(x*)* | D | Search Range |
| --- | --- | --- | --- | --- |
| 1 | Storn's Chebyshev Polynomial Fitting Problem | 1 | 9 | [-8192, 8192] |
| 2 | Inverse Hilbert Matrix Problem | 1 | 16 | [-16384, 16384] |
| 3 | Lennard-Jones Minimum Energy Cluster | 1 | 18 | [-4,4] |
| 4 | Rastrigin’s Function | 1 | 10 | [-100,100] |
| 5 | Griewangk’s Function | 1 | 10 | [-100,100] |
| 6 | Weierstrass Function | 1 | 10 | [-100,102] |
| 7 | Modified Schwefel’s Function | 1 | 10 | [-100,100] |
| 8 | Expanded Schaffer’s F6 Function | 1 | 10 | [-100,100] |
| 9 | Happy Cat Function | 1 | 10 | [-100,100] |
| 10 | Ackley Function | 1 | 10 | [-100,100] |

IEEE CEC 2022 benchmark functions:

|  | No. | Functions | Fi* |
| --- | --- | --- | --- |
| Unimodal  Function | 1 | Shifted and full Rotated Zakharov Function | 300 |
| Basic Functions | 2 | Shifted and full Rotated Rosenbrock’s Function | 400 |
|  | 3 | Shifted and full Rotated Expanded Schaffer’s f6 Function | 600 |
|  | 4 | Shifted and full Rotated Non-Continuous Rastrigin’s Function | 800 |
|  | 5 | Shifted and full Rotated Levy Function | 900 |
| Hybrid Functions | 6 | Hybrid Function 1 (N = 3) | 1800 |
|  | 7 | Hybrid Function 2 (N = 6) | 2000 |
|  | 8 | Hybrid Function 3 (N = 5) | 2200 |
| Composition Functions | 9 | Composition Function 1 (N = 5) | 2300 |
|  | 10 | Composition Function 2 (N = 4) | 2400 |
|  | 11 | Composition Function 3 (N = 5) | 2600 |
|  | 12 | Composition Function 4 (N = 6) | 2700 |
| Search range: [-100,100] | | | |

**Table A.2** The analysis results of AVG and STD of parameter $\rho$

|  | F1 |  | F2 |  | F3 |  |
| --- | --- | --- | --- | --- | --- | --- |
|  | AVG | STD | AVG | STD | AVG | STD |
| **0.1** | 2.7125E+06 | 3.1660E+06 | **9.2656E+03** | **7.0149E+03** | 3.8451E+03 | 1.8081E+03 |
| **0.2** | 2.6136E+06 | 4.7232E+06 | 1.0839E+04 | 8.8264E+03 | 3.6888E+03 | 1.9623E+03 |
| **0.3** | 1.6418E+06 | 2.9777E+06 | 1.3915E+04 | 1.0339E+04 | 3.9906E+03 | 1.6190E+03 |
| **0.4** | 1.0933E+06 | 8.7278E+05 | 1.2589E+04 | 9.0408E+03 | 4.0383E+03 | 1.8043E+03 |
| **0.5** | 9.7228E+05 | 7.8421E+05 | 1.1696E+04 | 8.4341E+03 | 3.9490E+03 | 1.7348E+03 |
| **0.6** | 8.4729E+05 | 7.4822E+05 | 1.2309E+04 | 9.8676E+03 | 4.3118E+03 | 1.9201E+03 |
| **0.7** | 8.1400E+05 | 6.8383E+05 | 1.1292E+04 | 9.5819E+03 | 3.9740E+03 | 1.6354E+03 |
| **0.8** | 8.5317E+05 | 5.1110E+05 | 1.3741E+04 | 1.0782E+04 | **3.6888E+03** | 2.0230E+03 |
| **0.9** | 7.5467E+05 | 5.4369E+05 | 1.4400E+04 | 8.9958E+03 | 3.8952E+03 | **1.6021E+03** |
| **1** | **5.9791E+05** | **3.3219E+05** | 1.3333E+04 | 1.1216E+04 | 4.5132E+03 | 1.9888E+03 |
|  | F4 |  | F5 |  | F6 |  |
|  | AVG | STD | AVG | STD | AVG | STD |
| **0.1** | 5.4453E+02 | 4.2926E+01 | 5.2000E+02 | 4.0000E-04 | **6.2903E+02** | **2.5854E+00** |
| **0.2** | 5.2569E+02 | 4.9913E+01 | 5.2000E+02 | 2.0000E-04 | 6.2913E+02 | 2.8203E+00 |
| **0.3** | 4.8916E+02 | **2.1213E+01** | 5.2000E+02 | 3.0000E-04 | 6.3162E+02 | 3.8207E+00 |
| **0.4** | 4.7926E+02 | 2.9900E+01 | **5.2000E+02** | 4.0000E-04 | 6.2940E+02 | 4.2935E+00 |
| **0.5** | 4.8152E+02 | 3.8682E+01 | 5.2000E+02 | **2.0000E-04** | 6.2994E+02 | 4.4996E+00 |
| **0.6** | 4.7279E+02 | 3.4949E+01 | 5.2000E+02 | 3.0000E-04 | 6.2970E+02 | 3.6522E+00 |
| **0.7** | 4.7156E+02 | 3.7882E+01 | 5.2000E+02 | 3.0000E-04 | 6.3126E+02 | 3.2530E+00 |
| **0.8** | **4.6018E+02** | 3.4653E+01 | 5.2000E+02 | 3.0000E-04 | 6.3039E+02 | 3.2246E+00 |
| **0.9** | 4.6564E+02 | 3.2241E+01 | 5.2000E+02 | 2.0000E-04 | 6.3124E+02 | 2.8451E+00 |
| **1** | 4.6023E+02 | 3.8651E+01 | 5.2000E+02 | 2.0000E-04 | 6.3110E+02 | 3.0946E+00 |
|  | F7 |  | F8 |  | F9 |  |
|  | AVG | STD | AVG | STD | AVG | STD |
| **0.1** | 7.0001E+02 | 1.6000E-02 | 9.0834E+02 | 1.6129E+01 | 1.0839E+03 | 1.3647E+01 |
| **0.2** | 7.0002E+02 | 1.2800E-02 | 9.0753E+02 | 1.7070E+01 | **1.0778E+03** | 1.2709E+01 |
| **0.3** | 7.0002E+02 | 1.3000E-02 | 9.0767E+02 | 1.7782E+01 | 1.0875E+03 | 1.6042E+01 |
| **0.4** | 7.0002E+02 | 4.0300E-02 | 9.0837E+02 | 1.4576E+01 | 1.0857E+03 | 1.2934E+01 |
| **0.5** | 7.0001E+02 | 1.5900E-02 | 9.0983E+02 | 1.4601E+01 | 1.0819E+03 | **1.0353E+01** |
| **0.6** | 7.0001E+02 | **9.5000E-03** | 9.0903E+02 | **1.1459E+01** | 1.0846E+03 | 1.3889E+01 |
| **0.7** | **7.0001E+02** | 1.0100E-02 | 9.1043E+02 | 1.8249E+01 | 1.0832E+03 | 1.2914E+01 |
| **0.8** | 7.0001E+02 | 1.1400E-02 | 9.0899E+02 | 1.3766E+01 | 1.0815E+03 | 1.3501E+01 |
| **0.9** | 7.0002E+02 | 1.7900E-02 | 9.1106E+02 | 1.8092E+01 | 1.0831E+03 | 1.3429E+01 |
| **1** | 7.0001E+02 | 1.3500E-02 | **9.0727E+02** | 1.2728E+01 | 1.0819E+03 | 1.2084E+01 |
|  | F10 |  | F11 |  | F12 |  |
|  | AVG | STD | AVG | STD | AVG | STD |
| **0.1** | 2.5571E+03 | 6.3607E+02 | 5.1792E+03 | **4.9596E+02** | 1.2011E+03 | 4.7550E-01 |
| **0.2** | 2.7416E+03 | 6.4781E+02 | 5.1282E+03 | 5.8988E+02 | 1.2009E+03 | 3.4030E-01 |
| **0.3** | 2.5173E+03 | 5.2091E+02 | 5.1441E+03 | 6.4193E+02 | 1.2009E+03 | 3.7210E-01 |
| **0.4** | 2.6764E+03 | 6.9609E+02 | **5.0058E+03** | 6.5013E+02 | 1.2008E+03 | 3.4790E-01 |
| **0.5** | 2.7941E+03 | 8.7168E+02 | 5.0600E+03 | 6.3322E+02 | 1.2008E+03 | 2.9850E-01 |
| **0.6** | 2.7027E+03 | **4.8874E+02** | 5.1480E+03 | 5.7605E+02 | 1.2008E+03 | 3.1770E-01 |
| **0.7** | 2.4637E+03 | 5.1378E+02 | 5.1580E+03 | 6.4341E+02 | 1.2007E+03 | 2.9990E-01 |
| **0.8** | 2.6289E+03 | 5.7225E+02 | 5.2055E+03 | 6.9109E+02 | 1.2007E+03 | 3.3030E-01 |
| **0.9** | **2.3939E+03** | 6.1055E+02 | 5.0752E+03 | 5.5200E+02 | 1.2007E+03 | **2.7280E-01** |
| **1** | 2.7551E+03 | 5.8273E+02 | 5.2839E+03 | 7.0652E+02 | **1.2007E+03** | 3.2210E-01 |
|  | F13 |  | F14 |  | F15 |  |
|  | AVG | STD | AVG | STD | AVG | STD |
| **0.1** | 1.3005E+03 | **8.3200E-02** | 1.4003E+03 | 1.4650E-01 | 1.5445E+03 | 1.6369E+01 |
| **0.2** | 1.3005E+03 | 1.3130E-01 | 1.4003E+03 | 1.1840E-01 | 1.5524E+03 | 1.9619E+01 |
| **0.3** | 1.3005E+03 | 9.2700E-02 | 1.4003E+03 | 5.4000E-02 | **1.5426E+03** | 1.5316E+01 |
| **0.4** | **1.3005E+03** | 1.1730E-01 | 1.4003E+03 | 1.4090E-01 | 1.5521E+03 | 1.5742E+01 |
| **0.5** | 1.3005E+03 | 1.3220E-01 | 1.4003E+03 | **4.2200E-02** | 1.5453E+03 | **1.2521E+01** |
| **0.6** | 1.3005E+03 | 9.9600E-02 | 1.4003E+03 | 1.0160E-01 | 1.5577E+03 | 2.3872E+01 |
| **0.7** | 1.3005E+03 | 1.2510E-01 | 1.4003E+03 | 5.6800E-02 | 1.5546E+03 | 2.4211E+01 |
| **0.8** | 1.3005E+03 | 1.0730E-01 | 1.4003E+03 | 5.0200E-02 | 1.5527E+03 | 2.2505E+01 |
| **0.9** | 1.3005E+03 | 1.0240E-01 | 1.4003E+03 | 5.3000E-02 | 1.5650E+03 | 2.0096E+01 |
| **1** | 1.3005E+03 | 1.1150E-01 | **1.4003E+03** | 5.6700E-02 | 1.5546E+03 | 1.7988E+01 |
|  | F16 |  | F17 |  | F18 |  |
|  | AVG | STD | AVG | STD | AVG | STD |
| **0.1** | **1.6122E+03** | 5.3980E-01 | 3.5980E+05 | 2.7732E+05 | 4.2664E+03 | 2.4081E+03 |
| **0.2** | 1.6123E+03 | 4.5950E-01 | 5.0520E+05 | 2.9644E+05 | 3.6267E+03 | 2.2548E+03 |
| **0.3** | 1.6122E+03 | 5.3670E-01 | 3.8952E+05 | **1.9944E+05** | 3.6291E+03 | 2.3796E+03 |
| **0.4** | 1.6123E+03 | 4.6000E-01 | 3.8342E+05 | 3.1018E+05 | 3.7782E+03 | 2.9724E+03 |
| **0.5** | 1.6122E+03 | 6.1140E-01 | 5.1264E+05 | 4.1999E+05 | 1.1959E+04 | 4.6647E+04 |
| **0.6** | 1.6124E+03 | 4.8700E-01 | **3.2964E+05** | 2.2577E+05 | **3.2909E+03** | **1.3477E+03** |
| **0.7** | 1.6122E+03 | 5.2490E-01 | 4.2710E+05 | 3.0645E+05 | 3.9584E+03 | 2.3798E+03 |
| **0.8** | 1.6123E+03 | **3.2290E-01** | 4.1415E+05 | 3.0760E+05 | 4.5155E+03 | 3.6910E+03 |
| **0.9** | 1.6122E+03 | 5.7170E-01 | 4.4283E+05 | 2.7282E+05 | 4.3799E+03 | 4.0313E+03 |
| **1** | 1.6122E+03 | 5.5600E-01 | 4.5593E+05 | 3.1048E+05 | 3.5031E+03 | 2.7662E+03 |
|  | F19 |  | F20 |  | F21 |  |
|  | AVG | STD | AVG | STD | AVG | STD |
| **0.1** | 1.9164E+03 | 9.6520E+00 | 5.0155E+03 | 2.6860E+03 | **3.1281E+05** | **1.9655E+05** |
| **0.2** | 1.9157E+03 | 3.5826E+00 | 3.8472E+03 | 1.4262E+03 | 3.8873E+05 | 3.6056E+05 |
| **0.3** | 1.9162E+03 | 3.6913E+00 | 4.3183E+03 | 1.3867E+03 | 4.3811E+05 | 3.0963E+05 |
| **0.4** | **1.9147E+03** | 4.5328E+00 | 4.1973E+03 | 2.2481E+03 | 5.2829E+05 | 4.5761E+05 |
| **0.5** | 1.9173E+03 | 7.0587E+00 | 3.7225E+03 | 1.4111E+03 | 4.6072E+05 | 3.7826E+05 |
| **0.6** | 1.9166E+03 | 6.0771E+00 | 4.5173E+03 | 1.7538E+03 | 4.0580E+05 | 3.1585E+05 |
| **0.7** | 1.9150E+03 | 3.3394E+00 | 3.8036E+03 | 1.3169E+03 | 4.6881E+05 | 4.1086E+05 |
| **0.8** | 1.9149E+03 | 3.5611E+00 | 3.6123E+03 | **1.0792E+03** | 4.6498E+05 | 4.1177E+05 |
| **0.9** | 1.9148E+03 | **3.3168E+00** | **3.3641E+03** | 1.2554E+03 | 5.0503E+05 | 4.3101E+05 |
| **1** | 1.9167E+03 | 4.8131E+00 | 3.5509E+03 | 1.4341E+03 | 4.4047E+05 | 2.5815E+05 |
|  | F22 |  | F23 |  | F24 |  |
|  | AVG | STD | AVG | STD | AVG | STD |
| **0.1** | 2.8764E+03 | 1.9110E+02 | **2.5000E+03** | 0.0000E+00 | 2.6001E+03 | 5.5600E-02 |
| **0.2** | 2.8218E+03 | 2.3991E+02 | 2.5000E+03 | 0.0000E+00 | 2.6001E+03 | 6.5900E-02 |
| **0.3** | 2.8262E+03 | 1.8314E+02 | 2.5000E+03 | **0.0000E+00** | 2.6001E+03 | 3.5200E-02 |
| **0.4** | 2.8731E+03 | 2.2235E+02 | 2.5000E+03 | 0.0000E+00 | 2.6001E+03 | 1.0300E-01 |
| **0.5** | **2.7761E+03** | **1.4805E+02** | 2.5000E+03 | 0.0000E+00 | 2.6001E+03 | 5.6300E-02 |
| **0.6** | 2.8520E+03 | 2.0700E+02 | 2.5000E+03 | 0.0000E+00 | 2.6001E+03 | 4.5500E-02 |
| **0.7** | 2.8734E+03 | 1.9327E+02 | 2.5000E+03 | 0.0000E+00 | 2.6001E+03 | 5.8300E-02 |
| **0.8** | 2.8407E+03 | 2.3859E+02 | 2.5000E+03 | 0.0000E+00 | 2.6001E+03 | 6.5300E-02 |
| **0.9** | 2.8652E+03 | 2.0639E+02 | 2.5000E+03 | 0.0000E+00 | **2.6000E+03** | **3.3700E-02** |
| **1** | 2.8025E+03 | 1.9361E+02 | 2.5000E+03 | 0.0000E+00 | 2.6001E+03 | 4.9000E-02 |
|  | F25 |  | F26 |  | F27 |  |
|  | AVG | STD | AVG | STD | AVG | STD |
| **0.1** | **2.7000E+03** | **0.0000E+00** | 2.7005E+03 | 1.1850E-01 | **2.9000E+03** | 0.0000E+00 |
| **0.2** | 2.7000E+03 | 0.0000E+00 | **2.7005E+03** | 1.1510E-01 | 2.9000E+03 | 0.0000E+00 |
| **0.3** | 2.7000E+03 | 0.0000E+00 | 2.7005E+03 | 1.3270E-01 | 2.9000E+03 | 0.0000E+00 |
| **0.4** | 2.7000E+03 | 0.0000E+00 | 2.7005E+03 | 1.4290E-01 | 2.9000E+03 | 0.0000E+00 |
| **0.5** | 2.7000E+03 | 0.0000E+00 | 2.7005E+03 | 1.0850E-01 | 2.9000E+03 | 0.0000E+00 |
| **0.6** | 2.7000E+03 | 0.0000E+00 | 2.7005E+03 | 1.3180E-01 | 2.9000E+03 | 0.0000E+00 |
| **0.7** | 2.7000E+03 | 0.0000E+00 | 2.7005E+03 | **1.0170E-01** | 2.9000E+03 | 0.0000E+00 |
| **0.8** | 2.7000E+03 | 0.0000E+00 | 2.7005E+03 | 1.1080E-01 | 2.9000E+03 | 0.0000E+00 |
| **0.9** | 2.7000E+03 | 0.0000E+00 | 2.7005E+03 | 1.2410E-01 | 2.9000E+03 | **0.0000E+00** |
| **1** | 2.7000E+03 | 0.0000E+00 | 2.7005E+03 | 1.2730E-01 | 2.9000E+03 | 0.0000E+00 |
|  | F28 |  | F29 |  | F30 |  |
|  | AVG | STD | AVG | STD | AVG | STD |
| **0.1** | **3.0000E+03** | 0.0000E+00 | 3.1000E+03 | 0.0000E+00 | 3.2000E+03 | 4.9000E-03 |
| **0.2** | 3.0000E+03 | 0.0000E+00 | 3.1000E+03 | 0.0000E+00 | 3.2000E+03 | 5.0000E-03 |
| **0.3** | 3.0000E+03 | 0.0000E+00 | **3.1000E+03** | 0.0000E+00 | 3.2000E+03 | 4.8000E-03 |
| **0.4** | 3.0000E+03 | 0.0000E+00 | 3.1000E+03 | 0.0000E+00 | 3.2000E+03 | **3.6000E-03** |
| **0.5** | 3.0000E+03 | 0.0000E+00 | 3.1000E+03 | 0.0000E+00 | 3.2000E+03 | 4.4000E-03 |
| **0.6** | 3.0000E+03 | 0.0000E+00 | 3.1000E+03 | 0.0000E+00 | **3.2000E+03** | 3.7000E-03 |
| **0.7** | 3.0000E+03 | 0.0000E+00 | 3.1000E+03 | 0.0000E+00 | 3.2000E+03 | 4.7000E-03 |
| **0.8** | 3.0000E+03 | 0.0000E+00 | 3.1000E+03 | **0.0000E+00** | 3.2000E+03 | 3.6000E-03 |
| **0.9** | 3.0000E+03 | 0.0000E+00 | 3.1000E+03 | 0.0000E+00 | 3.2000E+03 | 4.3000E-03 |
| **1** | 3.0000E+03 | **0.0000E+00** | 3.1000E+03 | 0.0000E+00 | 3.2000E+03 | 4.2000E-03 |

**Table A.3** The analysis results of AVG and STD of various CAGWOAs

|  | F1 |  | F2 |  | F3 |  |
| --- | --- | --- | --- | --- | --- | --- |
|  | AVG | STD | AVG | STD | AVG | STD |
| **CAGWOA** | **1.4602E+06** | 1.0667E+06 | 1.2495E+04 | 8.8656E+03 | 4.1629E+03 | 1.9097E+03 |
| **WOA** | 3.1377E+07 | 1.1213E+07 | 2.9375E+06 | 1.7291E+06 | 3.3434E+04 | 2.0296E+04 |
| **CWOA** | 2.4033E+07 | 8.9429E+06 | 2.8249E+06 | 1.6850E+06 | 2.0541E+04 | 1.0094E+04 |
| **AWOA** | 3.7433E+06 | 2.9218E+06 | 1.0361E+04 | 7.9760E+03 | 1.2819E+04 | 1.1336E+04 |
| **GWOA** | 4.1116E+06 | 2.7624E+06 | 1.4001E+04 | 1.2800E+04 | 9.6246E+03 | 1.0672E+04 |
| **CAWOA** | 2.8693E+06 | 2.6856E+06 | **6.7450E+03** | **6.3601E+03** | 6.8114E+03 | 2.1969E+03 |
| **CGWOA** | 2.2256E+06 | **1.0019E+06** | 1.1180E+04 | 9.6419E+03 | **3.4825E+03** | **1.4795E+03** |
| **AGWOA** | 2.5164E+06 | 1.8831E+06 | 1.4456E+04 | 1.0388E+04 | 6.8821E+03 | 5.6063E+03 |
|  | F4 |  | F5 |  | F6 |  |
|  | AVG | STD | AVG | STD | AVG | STD |
| **CAGWOA** | **4.9416E+02** | **3.1390E+01** | **5.2000E+02** | **0.0000E+00** | 6.2925E+02 | 4.2465E+00 |
| **WOA** | 5.7458E+02 | 4.0820E+01 | 5.2032E+02 | 2.2000E-01 | 6.3533E+02 | 4.1285E+00 |
| **CWOA** | 5.8568E+02 | 5.9840E+01 | 5.2003E+02 | 5.0000E-02 | 6.3184E+02 | 2.9414E+00 |
| **AWOA** | 5.0831E+02 | 5.0360E+01 | 5.2000E+02 | 0.0000E+00 | 6.3622E+02 | 3.6786E+00 |
| **GWOA** | 5.1074E+02 | 4.4500E+01 | 5.2016E+02 | 1.4000E-01 | 6.3209E+02 | 4.3549E+00 |
| **CAWOA** | 4.9949E+02 | 4.0080E+01 | 5.2000E+02 | 0.0000E+00 | 6.3417E+02 | **2.9367E+00** |
| **CGWOA** | 5.2416E+02 | 3.2710E+01 | 5.2000E+02 | 0.0000E+00 | **6.2789E+02** | 3.4069E+00 |
| **AGWOA** | **4.9209E+02** | 3.4520E+01 | 5.2000E+02 | **0.0000E+00** | 6.3276E+02 | 3.8906E+00 |
|  | F7 |  | F8 |  | F9 |  |
|  | AVG | STD | AVG | STD | AVG | STD |
| **CAGWOA** | 7.0001E+02 | 2.0000E-02 | 9.1459E+02 | 1.4150E+01 | **1.0860E+03** | 1.1281E+01 |
| **WOA** | 7.0103E+02 | 6.0000E-02 | 9.8560E+02 | 4.3220E+01 | 1.1142E+03 | 4.1342E+01 |
| **CWOA** | 7.0101E+02 | 8.0000E-02 | 9.6205E+02 | 2.1080E+01 | 1.0939E+03 | **1.0947E+01** |
| **AWOA** | **7.0001E+02** | **1.0000E-02** | 9.7415E+02 | 3.7890E+01 | 1.1444E+03 | 7.8166E+01 |
| **GWOA** | 7.0004E+02 | 3.0000E-02 | 9.3431E+02 | 3.0520E+01 | 1.1439E+03 | 6.4071E+01 |
| **CAWOA** | 7.0001E+02 | 1.0000E-02 | 9.6406E+02 | 1.4330E+01 | 1.0956E+03 | 1.1307E+01 |
| **CGWOA** | 7.0004E+02 | 3.0000E-02 | **9.0244E+02** | **1.1930E+01** | 1.0886E+03 | 1.3792E+01 |
| **AGWOA** | 7.0001E+02 | 1.0000E-02 | 9.4228E+02 | 3.0290E+01 | 1.1595E+03 | 7.7208E+01 |
|  | F10 |  | F11 |  | F12 |  |
|  | AVG | STD | AVG | STD | AVG | STD |
| **CAGWOA** | 2.7442E+03 | 9.1402E+02 | 5.2115E+03 | 5.6787E+02 | 1.2009E+03 | 3.8140E-01 |
| **WOA** | 4.8309E+03 | 7.5833E+02 | 5.9443E+03 | 9.2010E+02 | 1.2016E+03 | 3.9580E-01 |
| **CWOA** | 4.3062E+03 | 5.3534E+02 | 5.5694E+03 | 5.6171E+02 | 1.2013E+03 | 4.1610E-01 |
| **AWOA** | 4.6678E+03 | 7.6586E+02 | 5.6479E+03 | 6.7088E+02 | 1.2012E+03 | 4.1030E-01 |
| **GWOA** | 3.5076E+03 | 6.9459E+02 | 5.3541E+03 | 8.6415E+02 | 1.2011E+03 | **3.5530E-01** |
| **CAWOA** | 4.3577E+03 | 5.1959E+02 | 5.3859E+03 | **5.5258E+02** | 1.2012E+03 | 4.8150E-01 |
| **CGWOA** | **2.2206E+03** | **5.0423E+02** | **5.0110E+03** | 5.7734E+02 | **1.2008E+03** | 3.8020E-01 |
| **AGWOA** | 3.9769E+03 | 5.3273E+02 | 5.4298E+03 | 7.1694E+02 | 1.2010E+03 | 4.1790E-01 |
|  | F13 |  | F14 |  | F15 |  |
|  | AVG | STD | AVG | STD | AVG | STD |
| **CAGWOA** | **1.3005E+03** | 1.3000E-01 | 1.4003E+03 | 1.3000E-01 | 1.5501E+03 | 1.9233E+01 |
| **WOA** | 1.3005E+03 | 1.2000E-01 | **1.4003E+03** | 5.0000E-02 | 1.5744E+03 | 2.4217E+01 |
| **CWOA** | 1.3005E+03 | **1.0000E-01** | 1.4003E+03 | 5.0000E-02 | 1.5675E+03 | 2.5598E+01 |
| **AWOA** | 1.3005E+03 | 1.2000E-01 | 1.4003E+03 | **4.0000E-02** | 1.5998E+03 | 3.7730E+01 |
| **GWOA** | 1.3005E+03 | 1.2000E-01 | 1.4003E+03 | 1.1000E-01 | **1.5379E+03** | 1.3452E+01 |
| **CAWOA** | 1.3005E+03 | 1.1000E-01 | 1.4003E+03 | 5.0000E-02 | 1.6118E+03 | 5.3704E+01 |
| **CGWOA** | 1.3005E+03 | 1.1000E-01 | 1.4003E+03 | 9.0000E-02 | 1.5384E+03 | **1.3148E+01** |
| **AGWOA** | 1.3005E+03 | 1.4000E-01 | 1.4003E+03 | 1.3000E-01 | 1.5543E+03 | 2.1029E+01 |
|  | F16 |  | F17 |  | F18 |  |
|  | AVG | STD | AVG | STD | AVG | STD |
| **CAGWOA** | **1.6122E+03** | 5.2000E-01 | **3.6856E+05** | **3.0010E+05** | **3.4986E+03** | 3.0115E+03 |
| **WOA** | 1.6127E+03 | 4.7000E-01 | 3.9595E+06 | 3.0825E+06 | 1.2601E+04 | 3.6250E+04 |
| **CWOA** | 1.6124E+03 | **3.9000E-01** | 2.4791E+06 | 2.2981E+06 | 3.6030E+03 | 2.4819E+03 |
| **AWOA** | 1.6126E+03 | 5.9000E-01 | 9.5192E+05 | 5.3213E+05 | 1.4584E+04 | 2.5097E+04 |
| **GWOA** | 1.6123E+03 | 4.7000E-01 | 1.3672E+06 | 1.0169E+06 | 5.7675E+03 | 3.9406E+03 |
| **CAWOA** | 1.6125E+03 | 5.6000E-01 | 4.4260E+05 | 3.7833E+05 | 4.1175E+03 | 2.3056E+03 |
| **CGWOA** | 1.6122E+03 | 5.3000E-01 | 7.1415E+05 | 3.3378E+05 | 3.8482E+03 | **1.8125E+03** |
| **AGWOA** | 1.6127E+03 | 4.3000E-01 | 8.1438E+05 | 6.8402E+05 | 1.2296E+04 | 1.9842E+04 |
|  | F19 |  | F20 |  | F21 |  |
|  | AVG | STD | AVG | STD | AVG | STD |
| **CAGWOA** | **1.9156E+03** | 3.5900E+00 | **3.6295E+03** | **1.4936E+03** | 5.8414E+05 | 4.7559E+05 |
| **WOA** | 1.9506E+03 | 3.8050E+01 | 3.2916E+04 | 2.0100E+04 | 1.7735E+06 | 2.1602E+06 |
| **CWOA** | 1.9252E+03 | 1.0860E+01 | 1.9304E+04 | 9.8066E+03 | 7.5594E+05 | 5.3667E+05 |
| **AWOA** | 1.9311E+03 | 1.1680E+01 | 1.7690E+04 | 9.6755E+03 | 8.1260E+05 | 5.3546E+05 |
| **GWOA** | 1.9365E+03 | 3.8150E+01 | 5.0823E+03 | 2.8675E+03 | 6.1118E+05 | 3.8778E+05 |
| **CAWOA** | 1.9289E+03 | 8.0300E+00 | 1.0685E+04 | 4.3977E+03 | 6.5128E+05 | 6.7415E+05 |
| **CGWOA** | 1.9156E+03 | **1.8300E+00** | 4.1886E+03 | 2.2963E+03 | **3.9240E+05** | **2.3807E+05** |
| **AGWOA** | 1.9188E+03 | 1.3800E+01 | 6.5992E+03 | 3.4630E+03 | 8.3150E+05 | 7.7165E+05 |
|  | F22 |  | F23 |  | F24 |  |
|  | AVG | STD | AVG | STD | AVG | STD |
| **CAGWOA** | 2.8185E+03 | **1.7567E+02** | 2.5000E+03 | 0.0000E+00 | 2.6000E+03 | **3.8900E-02** |
| **WOA** | 2.9953E+03 | 2.3637E+02 | 2.6364E+03 | 1.2860E+01 | 2.6053E+03 | 3.2639E+00 |
| **CWOA** | 3.0150E+03 | 2.6689E+02 | **2.5000E+03** | 0.0000E+00 | 2.6000E+03 | 5.8400E-02 |
| **AWOA** | 2.9601E+03 | 2.4594E+02 | 2.6182E+03 | 5.6500E+00 | 2.6108E+03 | 2.2309E+01 |
| **GWOA** | **2.8083E+03** | 2.1663E+02 | 2.6156E+03 | 4.7000E-01 | 2.6319E+03 | 6.8891E+00 |
| **CAWOA** | 2.9291E+03 | 2.2905E+02 | 2.5000E+03 | **0.0000E+00** | **2.6000E+03** | 4.5800E-02 |
| **CGWOA** | 2.8116E+03 | 1.9191E+02 | 2.5000E+03 | 0.0000E+00 | 2.6001E+03 | 4.7200E-02 |
| **AGWOA** | 2.8525E+03 | 1.9720E+02 | 2.6151E+03 | 2.2900E+00 | 2.6427E+03 | 2.4375E+01 |
|  | F25 |  | F26 |  | F27 |  |
|  | AVG | STD | AVG | STD | AVG | STD |
| **CAGWOA** | 2.7000E+03 | 0.0000E+00 | 2.7005E+03 | 1.3000E-01 | 2.9000E+03 | 0.0000E+00 |
| **WOA** | 2.7153E+03 | 1.5970E+01 | **2.7004E+03** | 1.3000E-01 | 3.8738E+03 | 3.0114E+02 |
| **CWOA** | **2.7000E+03** | **0.0000E+00** | 2.7469E+03 | 5.0480E+01 | **2.9000E+03** | **0.0000E+00** |
| **AWOA** | 2.7143E+03 | 1.4990E+01 | 2.7005E+03 | 1.6000E-01 | 3.7405E+03 | 3.8909E+02 |
| **GWOA** | 2.7218E+03 | 1.0770E+01 | 2.7005E+03 | **1.2000E-01** | 3.7267E+03 | 3.2167E+02 |
| **CAWOA** | 2.7000E+03 | 0.0000E+00 | 2.7535E+03 | 5.0510E+01 | 2.9000E+03 | 0.0000E+00 |
| **CGWOA** | 2.7000E+03 | 0.0000E+00 | 2.7005E+03 | 1.4000E-01 | 2.9000E+03 | 0.0000E+00 |
| **AGWOA** | 2.7231E+03 | 9.0000E+00 | 2.7005E+03 | 1.2000E-01 | 3.7804E+03 | 2.4457E+02 |
|  | F28 |  | F29 |  | F30 |  |
|  | AVG | STD | AVG | STD | AVG | STD |
| **CAGWOA** | 3.0000E+03 | 0.0000E+00 | 3.1000E+03 | 0.0000E+00 | 3.2000E+03 | 4.3000E-03 |
| **WOA** | 5.2381E+03 | 5.0390E+02 | 5.0119E+06 | 5.2052E+06 | 9.6365E+04 | 9.7155E+04 |
| **CWOA** | **3.0000E+03** | **0.0000E+00** | **3.1000E+03** | **0.0000E+00** | **3.2000E+03** | **1.5000E-03** |
| **AWOA** | 5.1535E+03 | 6.8187E+02 | 1.9570E+05 | 1.4566E+05 | 2.0008E+04 | 1.2942E+04 |
| **GWOA** | 4.8361E+03 | 3.7755E+02 | 6.6836E+06 | 3.7493E+06 | 2.0179E+04 | 1.0744E+04 |
| **CAWOA** | 3.0000E+03 | 0.0000E+00 | 3.1000E+03 | 0.0000E+00 | 3.2000E+03 | 2.1000E-03 |
| **CGWOA** | 3.0000E+03 | 0.0000E+00 | 3.1000E+03 | 0.0000E+00 | 3.2000E+03 | 4.5000E-03 |
| **AGWOA** | 4.8238E+03 | 6.4657E+02 | 1.5221E+05 | 9.0506E+04 | 1.0023E+04 | 7.3012E+03 |

**Table A.4** The analysis results of AVG and STD of WOA variants

|  | F1 |  | F2 |  | F3 |  |
| --- | --- | --- | --- | --- | --- | --- |
|  | AVG | STD | AVG | STD | AVG | STD |
| **CAGWOA** | 1.4039E+06 | 1.4333E+06 | **1.5172E+04** | **1.0361E+04** | 4.0299E+03 | 1.6578E+03 |
| **WOA** | 3.1629E+07 | 1.4570E+07 | 5.6182E+06 | 1.0841E+07 | 4.0307E+04 | 2.5568E+04 |
| **PSO** | 7.7878E+06 | 2.0072E+06 | 1.4800E+08 | 2.1714E+07 | 9.2916E+02 | **1.1963E+02** |
| **SCA** | 2.4482E+08 | 8.2832E+07 | 1.6758E+10 | 3.5562E+09 | 3.6560E+04 | 4.3258E+03 |
| **BA** | **8.0149E+05** | **3.5545E+05** | 5.7485E+05 | 3.3894E+05 | **4.4129E+02** | 2.0976E+02 |
| **GWO** | 4.9423E+07 | 3.0306E+07 | 1.8427E+09 | 1.4704E+09 | 2.9961E+04 | 9.8990E+03 |
| **FA** | 2.4527E+08 | 4.7415E+07 | 1.6006E+10 | 1.9605E+09 | 6.3045E+04 | 9.9644E+03 |
| **WDO** | 1.3761E+07 | 3.9930E+06 | 5.7434E+07 | 1.4748E+07 | 3.0293E+03 | 7.7096E+02 |
| **MFO** | 8.6626E+07 | 1.0618E+08 | 1.2467E+10 | 6.4064E+09 | 9.1654E+04 | 5.3649E+04 |
|  | F4 |  | F5 |  | F6 |  |
|  | AVG | STD | AVG | STD | AVG | STD |
| **CAGWOA** | 4.9067E+02 | **2.7822E+01** | **5.2000E+02** | **0.0000E+00** | 6.2986E+02 | 4.1310E+00 |
| **WOA** | 5.9038E+02 | 5.7756E+01 | 5.2034E+02 | 1.3100E-01 | 6.3579E+02 | 2.9930E+00 |
| **PSO** | 4.7604E+02 | 3.7448E+01 | 5.2095E+02 | 5.0000E-02 | 6.2302E+02 | 3.5130E+00 |
| **SCA** | 1.3891E+03 | 2.3767E+02 | 5.2094E+02 | 4.4000E-02 | 6.3372E+02 | 2.3020E+00 |
| **BA** | **4.3507E+02** | 3.4916E+01 | 5.2094E+02 | 6.3000E-02 | 6.3358E+02 | 3.7490E+00 |
| **GWO** | 6.5247E+02 | 8.7985E+01 | 5.2091E+02 | 1.5400E-01 | **6.1404E+02** | 2.6860E+00 |
| **FA** | 1.5510E+03 | 1.7481E+02 | 5.2095E+02 | 4.8000E-02 | 6.3363E+02 | **1.2040E+00** |
| **WDO** | 6.0072E+02 | 5.2068E+01 | 5.2086E+02 | 1.1100E-01 | 6.3299E+02 | 3.3490E+00 |
| **MFO** | 1.2697E+03 | 7.0381E+02 | 5.2026E+02 | 1.5900E-01 | 6.2437E+02 | 3.5390E+00 |
|  | F7 |  | F8 |  | F9 |  |
|  | AVG | STD | AVG | STD | AVG | STD |
| **CAGWOA** | **7.0001E+02** | **1.4000E-02** | 9.0725E+02 | 1.3906E+01 | 1.0823E+03 | 1.2684E+01 |
| **WOA** | 7.0101E+02 | 8.1000E-02 | 9.7307E+02 | 3.2824E+01 | 1.1356E+03 | 5.3976E+01 |
| **PSO** | 7.0226E+02 | 1.1800E-01 | 9.7459E+02 | 1.7654E+01 | 1.1097E+03 | 2.6925E+01 |
| **SCA** | 8.3978E+02 | 2.7958E+01 | 1.0453E+03 | 1.5394E+01 | 1.1690E+03 | 2.1490E+01 |
| **BA** | 7.0063E+02 | 1.4900E-01 | 1.0146E+03 | 5.0882E+01 | 1.1657E+03 | 7.1376E+01 |
| **GWO** | 7.2606E+02 | 2.3300E+01 | **8.8200E+02** | 1.6832E+01 | **9.9528E+02** | 1.8533E+01 |
| **FA** | 8.3413E+02 | 1.7555E+01 | 1.0238E+03 | **1.1233E+01** | 1.1613E+03 | **1.0914E+01** |
| **WDO** | 7.1064E+02 | 1.5440E+00 | 9.5725E+02 | 2.2444E+01 | 1.0790E+03 | 2.1060E+01 |
| **MFO** | 8.2768E+02 | 6.7942E+01 | 9.5142E+02 | 3.9631E+01 | 1.1148E+03 | 4.6832E+01 |
|  | F10 |  | F11 |  | F12 |  |
|  | AVG | STD | AVG | STD | AVG | STD |
| **CAGWOA** | **2.3787E+03** | 5.6144E+02 | 5.3158E+03 | 7.0811E+02 | 1.2009E+03 | 4.0800E-01 |
| **WOA** | 5.0291E+03 | 6.5068E+02 | 6.2852E+03 | 7.5371E+02 | 1.2019E+03 | 5.4200E-01 |
| **PSO** | 5.3086E+03 | 6.2933E+02 | 5.8806E+03 | 5.6380E+02 | 1.2024E+03 | **2.0000E-01** |
| **SCA** | 6.8722E+03 | 5.8474E+02 | 8.0554E+03 | **2.8750E+02** | 1.2024E+03 | 3.4900E-01 |
| **BA** | 5.5465E+03 | 5.3075E+02 | 5.7340E+03 | 7.1558E+02 | 1.2012E+03 | 3.8800E-01 |
| **GWO** | 3.0912E+03 | 5.3382E+02 | **3.9261E+03** | 5.3124E+02 | 1.2017E+03 | 1.1620E+00 |
| **FA** | 7.5513E+03 | **1.9616E+02** | 7.9657E+03 | 3.1803E+02 | 1.2025E+03 | 2.6700E-01 |
| **WDO** | 5.3969E+03 | 6.6111E+02 | 5.7388E+03 | 8.9365E+02 | 1.2014E+03 | 3.3200E-01 |
| **MFO** | 4.6545E+03 | 9.0732E+02 | 5.1671E+03 | 6.9051E+02 | **1.2005E+03** | 2.7000E-01 |
|  | F13 |  | F14 |  | F15 |  |
|  | AVG | STD | AVG | STD | AVG | STD |
| **CAGWOA** | 1.3005E+03 | 1.1000E-01 | 1.4003E+03 | 1.6600E-01 | 1.5566E+03 | 2.2461E+01 |
| **WOA** | 1.3006E+03 | 1.2500E-01 | 1.4003E+03 | 1.2000E-01 | 1.5703E+03 | 2.0239E+01 |
| **PSO** | **1.3004E+03** | 8.0000E-02 | **1.4003E+03** | 1.1100E-01 | **1.5168E+03** | **1.1210E+00** |
| **SCA** | 1.3029E+03 | 2.9400E-01 | 1.4441E+03 | 1.0712E+01 | 4.6273E+03 | 2.6506E+03 |
| **BA** | 1.3005E+03 | 1.5000E-01 | 1.4003E+03 | 1.2200E-01 | 1.5279E+03 | 4.7640E+00 |
| **GWO** | 1.3004E+03 | 8.7000E-02 | 1.4048E+03 | 7.2290E+00 | 1.5782E+03 | 9.9130E+01 |
| **FA** | 1.3027E+03 | 2.9600E-01 | 1.4404E+03 | 6.8630E+00 | 1.5777E+04 | 5.1501E+03 |
| **WDO** | 1.3005E+03 | **6.0000E-02** | 1.4003E+03 | **9.4000E-02** | 1.5301E+03 | 4.1490E+00 |
| **MFO** | 1.3022E+03 | 1.3780E+00 | 1.4319E+03 | 2.5466E+01 | 3.8420E+05 | 8.0631E+05 |
|  | F16 |  | F17 |  | F18 |  |
|  | AVG | STD | AVG | STD | AVG | STD |
| **CAGWOA** | 1.6123E+03 | 5.6000E-01 | 4.3908E+05 | 3.4062E+05 | **3.6452E+03** | **2.0328E+03** |
| **WOA** | 1.6125E+03 | 5.6500E-01 | 4.1794E+06 | 2.7998E+06 | 9.5404E+03 | 1.2440E+04 |
| **PSO** | 1.6120E+03 | 4.9900E-01 | 2.5865E+05 | 1.2297E+05 | 2.0951E+06 | 5.4319E+05 |
| **SCA** | 1.6128E+03 | 2.8100E-01 | 5.2519E+06 | 2.9136E+06 | 1.6700E+08 | 8.4485E+07 |
| **BA** | 1.6132E+03 | 3.2100E-01 | **9.6413E+04** | **5.2317E+04** | 8.3460E+04 | 3.3275E+04 |
| **GWO** | **1.6108E+03** | 7.3500E-01 | 1.3844E+06 | 1.2822E+06 | 7.4057E+06 | 2.3918E+07 |
| **FA** | 1.6129E+03 | **1.7700E-01** | 6.2929E+06 | 2.1005E+06 | 2.8828E+08 | 8.7966E+07 |
| **WDO** | 1.6130E+03 | 4.0300E-01 | 3.1067E+05 | 1.0880E+05 | 1.8978E+05 | 4.9139E+04 |
| **MFO** | 1.6128E+03 | 4.4200E-01 | 2.3220E+06 | 2.9765E+06 | 2.0420E+05 | 4.4331E+05 |
|  | F19 |  | F20 |  | F21 |  |
|  | AVG | STD | AVG | STD | AVG | STD |
| **CAGWOA** | **1.9160E+03** | 5.1360E+00 | 3.5387E+03 | 1.3174E+03 | 4.2631E+05 | 2.4848E+05 |
| **WOA** | 1.9539E+03 | 4.7014E+01 | 3.0147E+04 | 1.8266E+04 | 1.1227E+06 | 8.6353E+05 |
| **PSO** | 1.9180E+03 | **2.5480E+00** | **2.3236E+03** | **8.0000E+01** | 9.9089E+04 | 6.4381E+04 |
| **SCA** | 1.9972E+03 | 2.6184E+01 | 1.6059E+04 | 4.2697E+03 | 1.1852E+06 | 5.0460E+05 |
| **BA** | 1.9242E+03 | 2.2413E+01 | 2.3842E+03 | 1.4110E+02 | **6.5018E+04** | **3.0046E+04** |
| **GWO** | 1.9385E+03 | 2.5337E+01 | 1.6054E+04 | 6.5459E+03 | 7.8484E+05 | 1.8691E+06 |
| **FA** | 2.0036E+03 | 1.6137E+01 | 1.9233E+04 | 7.5457E+03 | 1.6708E+06 | 6.5313E+05 |
| **WDO** | 1.9384E+03 | 3.2442E+01 | 2.8212E+03 | 4.1900E+02 | 1.5999E+05 | 6.9561E+04 |
| **MFO** | 1.9808E+03 | 7.6633E+01 | 7.1476E+04 | 5.4665E+04 | 6.1530E+05 | 9.9705E+05 |
|  | F22 |  | F23 |  | F24 |  |
|  | AVG | STD | AVG | STD | AVG | STD |
| **CAGWOA** | 2.7826E+03 | 2.0182E+02 | **2.5000E+03** | **0.0000E+00** | 2.6001E+03 | 6.5000E-02 |
| **WOA** | 3.0125E+03 | 2.1968E+02 | 2.6343E+03 | 9.9780E+00 | 2.6065E+03 | 4.0310E+00 |
| **PSO** | 2.8772E+03 | 2.3187E+02 | 2.6159E+03 | 5.0400E-01 | 2.6279E+03 | 5.2490E+00 |
| **SCA** | 2.9805E+03 | 1.4718E+02 | 2.6654E+03 | 1.0753E+01 | 2.6001E+03 | 4.4000E-02 |
| **BA** | 3.3149E+03 | 3.0962E+02 | 2.6152E+03 | 3.0000E-03 | 2.6633E+03 | 2.1721E+01 |
| **GWO** | **2.5896E+03** | 1.7463E+02 | 2.6370E+03 | 1.2764E+01 | 2.6000E+03 | 1.0000E-03 |
| **FA** | 2.9312E+03 | **1.2759E+02** | 2.7356E+03 | 1.8666E+01 | 2.7068E+03 | 4.2790E+00 |
| **WDO** | 3.1755E+03 | 2.3848E+02 | 2.6054E+03 | 4.2032E+01 | **2.6000E+03** | **0.0000E+00** |
| **MFO** | 2.9573E+03 | 3.0256E+02 | 2.6610E+03 | 3.9138E+01 | 2.6819E+03 | 2.9514E+01 |
|  | F25 |  | F26 |  | F27 |  |
|  | AVG | STD | AVG | STD | AVG | STD |
| **CAGWOA** | 2.7000E+03 | 0.0000E+00 | 2.7005E+03 | **1.5000E-01** | **2.9000E+03** | **0.0000E+00** |
| **WOA** | 2.7258E+03 | 1.9020E+01 | **2.7005E+03** | 1.5300E-01 | 3.7334E+03 | 4.0160E+02 |
| **PSO** | 2.7117E+03 | 6.6190E+00 | 2.7738E+03 | 4.5016E+01 | 3.5187E+03 | 2.7641E+02 |
| **SCA** | 2.7241E+03 | 6.1110E+00 | 2.7025E+03 | 5.8600E-01 | 3.4858E+03 | 3.3295E+02 |
| **BA** | 2.7327E+03 | 1.3775E+01 | 2.7100E+03 | 5.1801E+01 | 3.7624E+03 | 4.4358E+02 |
| **GWO** | 2.7113E+03 | 4.7650E+00 | 2.7602E+03 | 4.9581E+01 | 3.3689E+03 | 8.8144E+01 |
| **FA** | 2.7345E+03 | 3.8970E+00 | 2.7023E+03 | 2.8100E-01 | 3.8044E+03 | 3.6159E+01 |
| **WDO** | **2.7000E+03** | **0.0000E+00** | 2.7901E+03 | 3.0283E+01 | 3.6127E+03 | 4.3373E+02 |
| **MFO** | 2.7155E+03 | 6.4650E+00 | 2.7023E+03 | 1.6020E+00 | 3.6117E+03 | 2.4202E+02 |
|  | F28 |  | F29 |  | F30 |  |
|  | AVG | STD | AVG | STD | AVG | STD |
| **CAGWOA** | **3.0000E+03** | **0.0000E+00** | **3.1000E+03** | **0.0000E+00** | **3.2000E+03** | **4.8000E-03** |
| **WOA** | 5.0639E+03 | 7.0734E+02 | 6.3186E+06 | 4.5776E+06 | 7.3672E+04 | 4.9428E+04 |
| **PSO** | 7.1157E+03 | 8.8667E+02 | 5.3110E+04 | 1.2114E+05 | 1.2237E+04 | 4.4893E+03 |
| **SCA** | 4.8343E+03 | 4.4486E+02 | 1.1721E+07 | 7.8868E+06 | 2.2939E+05 | 8.9883E+04 |
| **BA** | 5.5237E+03 | 5.0712E+02 | 3.5543E+07 | 3.3220E+07 | 1.1047E+04 | 3.9416E+03 |
| **GWO** | 3.9359E+03 | 2.1878E+02 | 1.5462E+06 | 4.1512E+06 | 4.7753E+04 | 2.5724E+04 |
| **FA** | 4.2296E+03 | 1.2674E+02 | 3.1790E+06 | 1.1469E+06 | 1.6095E+05 | 3.6744E+04 |
| **WDO** | 8.8539E+03 | 9.2298E+02 | 6.0030E+04 | 2.7369E+04 | 2.3152E+04 | 4.1517E+03 |
| **MFO** | 3.9707E+03 | 2.8255E+02 | 2.5081E+06 | 3.7997E+06 | 5.1366E+04 | 5.3904E+04 |

**Table A.5** The results of AVG and STD of CAGWOA and other improved algorithms on IEEE CEC 2014

|  | F1 |  | F2 |  | F3 |  |
| --- | --- | --- | --- | --- | --- | --- |
|  | AVG | STD | AVG | STD | AVG | STD |
| **CAGWOA** | **1.3617E+06** | **1.0792E+06** | 1.3052E+04 | 1.0507E+04 | **4.0560E+03** | 1.9288E+03 |
| **ASCA_PSO** | 3.3678E+07 | 4.9411E+07 | 9.2789E+08 | 1.3753E+09 | 1.9618E+04 | 6.1825E+03 |
| **CBA** | 4.5577E+06 | 1.8741E+06 | **1.2281E+04** | **9.2243E+03** | 5.0297E+03 | 5.4582E+03 |
| **CGSCA** | 2.7458E+08 | 7.6975E+07 | 1.8522E+10 | 4.1417E+09 | 4.1393E+04 | 4.9602E+03 |
| **HGWO** | 1.7239E+08 | 4.8461E+07 | 8.8659E+09 | 1.8995E+09 | 6.8249E+04 | 6.4079E+03 |
| **AMFOA** | 2.2168E+09 | 1.3987E+08 | 9.3728E+10 | 1.3863E+09 | 1.1972E+07 | 1.6961E+07 |
| **OBSCA** | 4.0152E+08 | 1.0432E+08 | 2.3405E+10 | 3.5029E+09 | 5.1396E+04 | 8.3462E+03 |
| **CIFOA** | 2.3486E+09 | 4.9564E+07 | 9.4540E+10 | 5.3526E+08 | 8.2745E+04 | **8.0940E+02** |
| **DSMFO** | 7.3818E+08 | 2.7365E+08 | 6.5007E+10 | 1.5269E+10 | 8.2989E+04 | 5.5766E+03 |
|  | F4 |  | F5 |  | F6 |  |
|  | AVG | STD | AVG | STD | AVG | STD |
| **CAGWOA** | **4.9560E+02** | **2.8055E+01** | **5.2000E+02** | **3.0000E-04** | 6.2864E+02 | 3.5844E+00 |
| **ASCA_PSO** | 5.7160E+02 | 1.5786E+02 | 5.2093E+02 | 5.4100E-02 | **6.2510E+02** | 3.4089E+00 |
| **CBA** | 4.9708E+02 | 3.8485E+01 | 5.2012E+02 | 1.2750E-01 | 6.4089E+02 | 3.4219E+00 |
| **CGSCA** | 1.6087E+03 | 2.5867E+02 | 5.2094E+02 | 5.0900E-02 | 6.3299E+02 | 2.4162E+00 |
| **HGWO** | 9.2506E+02 | 5.9714E+01 | 5.2081E+02 | 1.3020E-01 | 6.2551E+02 | 2.0399E+00 |
| **AMFOA** | 2.3750E+04 | 2.6894E+03 | 5.2117E+02 | 8.6200E-02 | 6.4864E+02 | 9.5290E-01 |
| **OBSCA** | 2.3956E+03 | 6.3226E+02 | 5.2097E+02 | 3.7000E-02 | 6.3193E+02 | 1.1943E+00 |
| **CIFOA** | 2.2145E+04 | 1.7023E+02 | 5.2106E+02 | 4.6600E-02 | 6.4763E+02 | **8.0750E-01** |
| **DSMFO** | 9.4097E+03 | 3.0445E+03 | 5.2098E+02 | 5.8200E-02 | 6.3902E+02 | 3.1831E+00 |
|  | F7 |  | F8 |  | F9 |  |
|  | AVG | STD | AVG | STD | AVG | STD |
| **CAGWOA** | **7.0001E+02** | **1.3000E-02** | **9.0823E+02** | 1.4768E+01 | **1.0792E+03** | 1.5574E+01 |
| **ASCA_PSO** | 7.0898E+02 | 1.3674E+01 | 9.6501E+02 | 2.2284E+01 | 1.1125E+03 | 3.1078E+01 |
| **CBA** | 7.0001E+02 | 1.6000E-02 | 1.0154E+03 | 4.9008E+01 | 1.1487E+03 | 6.3069E+01 |
| **CGSCA** | 8.7016E+02 | 3.4164E+01 | 1.0580E+03 | 1.6821E+01 | 1.1822E+03 | 1.5882E+01 |
| **HGWO** | 7.4526E+02 | 1.1796E+01 | 1.0036E+03 | 1.0678E+01 | 1.1326E+03 | 1.1472E+01 |
| **AMFOA** | 1.6666E+03 | 1.4952E+01 | 1.2892E+03 | 1.5928E+01 | 1.3005E+03 | **8.5510E-01** |
| **OBSCA** | 9.1320E+02 | 4.2053E+01 | 1.0593E+03 | 1.5712E+01 | 1.1983E+03 | 2.3841E+01 |
| **CIFOA** | 1.6859E+03 | 4.8880E+00 | 1.2209E+03 | **5.2670E+00** | 1.2499E+03 | 6.6872E+00 |
| **DSMFO** | 1.0862E+03 | 1.1826E+02 | 1.1158E+03 | 2.8977E+01 | 1.2625E+03 | 3.5018E+01 |
|  | F10 |  | F11 |  | F12 |  |
|  | AVG | STD | AVG | STD | AVG | STD |
| **CAGWOA** | **2.6689E+03** | 6.5890E+02 | **5.1969E+03** | 4.6101E+02 | **1.2007E+03** | 2.8580E-01 |
| **ASCA_PSO** | 5.2864E+03 | 5.6080E+02 | 6.3089E+03 | 1.0692E+03 | 1.2024E+03 | 2.7290E-01 |
| **CBA** | 5.9338E+03 | 6.9271E+02 | 5.8789E+03 | 7.7387E+02 | 1.2011E+03 | 6.2980E-01 |
| **CGSCA** | 6.9823E+03 | 4.9740E+02 | 8.0959E+03 | 2.8357E+02 | 1.2024E+03 | 3.0190E-01 |
| **HGWO** | 5.6941E+03 | 3.0821E+02 | 6.6908E+03 | 6.1558E+02 | 1.2013E+03 | 2.9310E-01 |
| **AMFOA** | 1.0759E+04 | 2.7361E+02 | 1.1588E+04 | 3.4545E+02 | 1.2081E+03 | 5.0390E-01 |
| **OBSCA** | 6.2446E+03 | 4.9814E+02 | 7.4802E+03 | 4.7834E+02 | 1.2023E+03 | 3.2540E-01 |
| **CIFOA** | 9.3462E+03 | **1.6346E+02** | 1.0360E+04 | **2.2722E+02** | 1.2025E+03 | **2.5770E-01** |
| **DSMFO** | 7.9028E+03 | 8.3188E+02 | 8.4361E+03 | 6.8962E+02 | 1.2029E+03 | 4.8800E-01 |
|  | F13 |  | F14 |  | F15 |  |
|  | AVG | STD | AVG | STD | AVG | STD |
| **CAGWOA** | 1.3005E+03 | **1.2900E-01** | **1.4003E+03** | **5.4000E-02** | **1.5418E+03** | 1.6471E+01 |
| **ASCA_PSO** | 1.3006E+03 | 1.4300E-01 | 1.4012E+03 | 2.2640E+00 | 1.5469E+03 | 8.8462E+01 |
| **CBA** | **1.3005E+03** | 1.4100E-01 | 1.4003E+03 | 6.4000E-02 | 1.5607E+03 | **1.5637E+01** |
| **CGSCA** | 1.3032E+03 | 2.8000E-01 | 1.4503E+03 | 9.3400E+00 | 7.1108E+03 | 4.9951E+03 |
| **HGWO** | 1.3019E+03 | 4.3600E-01 | 1.4227E+03 | 4.2560E+00 | 1.9114E+03 | 3.2258E+02 |
| **AMFOA** | 1.3099E+03 | 3.1800E-01 | 1.7369E+03 | 1.4295E+01 | 5.7869E+05 | 6.9709E+04 |
| **OBSCA** | 1.3036E+03 | 3.6800E-01 | 1.4646E+03 | 1.2418E+01 | 1.5711E+04 | 9.2506E+03 |
| **CIFOA** | 1.3103E+03 | 1.8800E-01 | 1.7773E+03 | 6.8780E+00 | 5.9385E+05 | 2.4100E+04 |
| **DSMFO** | 1.3069E+03 | 1.1980E+00 | 1.5461E+03 | 4.0693E+01 | 9.6910E+04 | 4.7354E+04 |
|  | F16 |  | F17 |  | F18 |  |
|  | AVG | STD | AVG | STD | AVG | STD |
| **CAGWOA** | **1.6123E+03** | 5.0100E-01 | 4.1110E+05 | 3.0280E+05 | **6.1989E+03** | 1.0166E+04 |
| **ASCA_PSO** | 1.6124E+03 | 3.4000E-01 | 1.0063E+06 | 9.3576E+05 | 3.3916E+06 | 1.2360E+06 |
| **CBA** | 1.6134E+03 | 3.6100E-01 | **2.5408E+05** | **1.2657E+05** | 8.5136E+03 | **8.1691E+03** |
| **CGSCA** | 1.6128E+03 | 2.2800E-01 | 6.3561E+06 | 2.8372E+06 | 1.4746E+08 | 8.2547E+07 |
| **HGWO** | 1.6126E+03 | 2.8500E-01 | 6.2390E+06 | 2.5443E+06 | 1.2973E+08 | 4.8928E+07 |
| **AMFOA** | 1.6144E+03 | 1.9900E-01 | 8.2185E+08 | 1.8479E+08 | 1.3132E+10 | 5.2821E+08 |
| **OBSCA** | 1.6130E+03 | 2.0700E-01 | 1.2550E+07 | 7.0307E+06 | 1.7716E+08 | 1.2779E+08 |
| **CIFOA** | 1.6139E+03 | **4.4000E-02** | 4.4415E+08 | 1.9349E+08 | 1.1775E+10 | 2.2086E+09 |
| **DSMFO** | 1.6127E+03 | 3.2500E-01 | 7.4511E+07 | 7.3398E+07 | 1.6666E+09 | 1.2030E+09 |
|  | F19 |  | F20 |  | F21 |  |
|  | AVG | STD | AVG | STD | AVG | STD |
| **CAGWOA** | **1.9152E+03** | **3.2790E+00** | 4.1299E+03 | 1.2834E+03 | 3.8673E+05 | 2.5491E+05 |
| **ASCA_PSO** | 1.9229E+03 | 2.6943E+01 | 6.2220E+03 | 2.7780E+03 | 2.7836E+05 | 2.3631E+05 |
| **CBA** | 1.9298E+03 | 2.9004E+01 | **2.9262E+03** | **8.2907E+02** | **1.0480E+05** | **6.2435E+04** |
| **CGSCA** | 2.0026E+03 | 2.6128E+01 | 2.0237E+04 | 7.5298E+03 | 1.7120E+06 | 7.9429E+05 |
| **HGWO** | 1.9931E+03 | 1.1496E+01 | 5.6135E+04 | 2.5381E+04 | 2.1593E+06 | 1.3742E+06 |
| **AMFOA** | 2.5422E+03 | 3.9054E+01 | 2.2121E+08 | 3.4048E+08 | 8.1554E+08 | 2.5024E+08 |
| **OBSCA** | 2.0123E+03 | 2.2953E+01 | 3.2290E+04 | 1.3043E+04 | 1.8419E+06 | 9.6818E+05 |
| **CIFOA** | 2.5970E+03 | 8.1730E+00 | 9.1717E+07 | 1.0914E+08 | 3.4061E+08 | 2.3506E+08 |
| **DSMFO** | 2.2889E+03 | 1.6984E+02 | 1.1689E+05 | 1.0460E+05 | 1.9544E+07 | 1.7794E+07 |
|  | F22 |  | F23 |  | F24 |  |
|  | AVG | STD | AVG | STD | AVG | STD |
| **CAGWOA** | 2.8424E+03 | 2.7242E+02 | 2.5000E+03 | 0.0000E+00 | 2.6001E+03 | 6.7900E-02 |
| **ASCA_PSO** | **2.7627E+03** | 1.6203E+02 | 2.6253E+03 | 6.3180E+00 | 2.6403E+03 | 8.4543E+00 |
| **CBA** | 3.3520E+03 | 3.4045E+02 | 2.6158E+03 | 2.9000E-01 | 2.6736E+03 | 2.8566E+01 |
| **CGSCA** | 3.0656E+03 | **1.3746E+02** | **2.5000E+03** | **0.0000E+00** | 2.6000E+03 | 4.0000E-06 |
| **HGWO** | 2.9478E+03 | 1.7115E+02 | 2.5167E+03 | 5.1155E+01 | **2.6000E+03** | **0.0000E+00** |
| **AMFOA** | 1.3657E+06 | 3.4258E+05 | 2.5000E+03 | 0.0000E+00 | 2.6000E+03 | 2.6000E-03 |
| **OBSCA** | 3.1268E+03 | 1.4973E+02 | 2.6846E+03 | 1.7357E+01 | 2.6000E+03 | 3.9600E-04 |
| **CIFOA** | 2.8513E+05 | 5.7037E+05 | 2.5000E+03 | 0.0000E+00 | 2.6000E+03 | 0.0000E+00 |
| **DSMFO** | 4.1158E+03 | 2.7039E+03 | 2.5000E+03 | 0.0000E+00 | 2.6000E+03 | 0.0000E+00 |
|  | F25 |  | F26 |  | F27 |  |
|  | AVG | STD | AVG | STD | AVG | STD |
| **CAGWOA** | 2.7000E+03 | 0.0000E+00 | **2.7005E+03** | 1.1200E-01 | 2.9000E+03 | 0.0000E+00 |
| **ASCA_PSO** | 2.7146E+03 | 6.3420E+00 | 2.7006E+03 | 1.5600E-01 | 3.5522E+03 | 2.2021E+02 |
| **CBA** | 2.7377E+03 | 1.4660E+01 | 2.7005E+03 | 1.5100E-01 | 3.9706E+03 | 4.5201E+02 |
| **CGSCA** | **2.7000E+03** | **0.0000E+00** | 2.7028E+03 | 5.0200E-01 | **2.9000E+03** | **0.0000E+00** |
| **HGWO** | 2.7000E+03 | 0.0000E+00 | 2.7672E+03 | 4.6040E+01 | 3.5684E+03 | 2.5010E+02 |
| **AMFOA** | 2.7000E+03 | 0.0000E+00 | 2.8000E+03 | **0.0000E+00** | 2.9000E+03 | 1.0000E-04 |
| **OBSCA** | 2.7000E+03 | 5.0000E-04 | 2.7040E+03 | 3.5600E-01 | 3.2497E+03 | 5.1160E+01 |
| **CIFOA** | 2.7000E+03 | 0.0000E+00 | 2.7944E+03 | 1.7113E+01 | 2.9000E+03 | 0.0000E+00 |
| **DSMFO** | 2.7000E+03 | 0.0000E+00 | 2.7723E+03 | 4.3054E+01 | 2.9000E+03 | 0.0000E+00 |
|  | F28 |  | F29 |  | F30 |  |
|  | AVG | STD | AVG | STD | AVG | STD |
| **CAGWOA** | 3.0000E+03 | 0.0000E+00 | 3.1000E+03 | 0.0000E+00 | 3.2000E+03 | 5.1000E-03 |
| **ASCA_PSO** | 4.4338E+03 | 3.8314E+02 | 4.4853E+06 | 6.5318E+06 | 4.0157E+04 | 3.5122E+04 |
| **CBA** | 5.3708E+03 | 7.7499E+02 | 2.8166E+07 | 3.1723E+07 | 1.6189E+04 | 8.0241E+03 |
| **CGSCA** | **3.0000E+03** | **0.0000E+00** | **3.1000E+03** | **0.0000E+00** | 2.0789E+04 | 6.7759E+04 |
| **HGWO** | 4.1136E+03 | 2.5383E+02 | 4.2440E+06 | 3.3981E+06 | 3.2001E+03 | 4.5060E-01 |
| **AMFOA** | 3.0000E+03 | 0.0000E+00 | 4.6618E+03 | 4.0051E+02 | 3.2952E+03 | 1.2328E+01 |
| **OBSCA** | 5.4389E+03 | 3.3920E+02 | 2.0982E+07 | 1.0536E+07 | 3.9275E+05 | 1.3786E+05 |
| **CIFOA** | 3.0000E+03 | 0.0000E+00 | 3.1000E+03 | 0.0000E+00 | **3.2000E+03** | **0.0000E+00** |
| **DSMFO** | 3.0000E+03 | 0.0000E+00 | 3.1000E+03 | 0.0000E+00 | 3.2000E+03 | 0.0000E+00 |

**Table A.6** The results of AVG and STD of CAGWOA and other improved algorithms on IEEE CEC 2019&2022

|  | F1 |  | F2 |  | F3 |  |
| --- | --- | --- | --- | --- | --- | --- |
|  | AVG | STD | AVG | STD | AVG | STD |
| **CAGWOA** | **1.0000E+00** | **0.0000E+00** | 4.9878E+00 | 4.8864E-02 | **1.7959E+00** | 1.4779E+00 |
| **ASCA_PSO** | 3.0936E+05 | 4.7877E+05 | 1.0505E+03 | 6.5164E+02 | 9.7511E+00 | 5.5716E-01 |
| **CBA** | 6.5607E+06 | 1.9556E+07 | 7.9144E+03 | 4.8433E+03 | 8.5401E+00 | 1.9709E+00 |
| **CGSCA** | 1.0000E+00 | 3.3454E-10 | **4.3022E+00** | 5.7827E-02 | 5.3690E+00 | 9.7922E-01 |
| **HGWO** | 1.0000E+00 | 0.0000E+00 | 4.3228E+00 | 1.9013E-01 | 3.8179E+00 | 2.0735E+00 |
| **AMFOA** | 1.0000E+00 | 7.2883E-08 | 5.0001E+00 | 3.4258E-05 | 1.1220E+06 | 3.5640E+06 |
| **OBSCA** | 1.0000E+00 | 1.3318E-10 | 1.0535E+01 | 2.6295E+01 | 1.9692E+00 | 5.5754E-01 |
| **CIFOA** | 1.0000E+00 | 0.0000E+00 | 5.0000E+00 | **0.0000E+00** | 1.2746E+01 | **3.8586E-01** |
| **DSMFO** | 1.0000E+00 | 0.0000E+00 | 5.0000E+00 | 0.0000E+00 | 4.8704E+00 | 8.4533E-01 |
|  | F4 |  | F5 |  | F6 |  |
|  | AVG | STD | AVG | STD | AVG | STD |
| **CAGWOA** | 3.8643E+01 | 1.0649E+01 | 1.6073E+00 | 2.8188E-01 | 5.6457E+00 | 1.2423E+00 |
| **ASCA_PSO** | **2.1088E+01** | 7.8285E+00 | 2.0150E+00 | **6.7369E-02** | **4.4891E+00** | 1.3560E+00 |
| **CBA** | 6.2906E+01 | 2.2463E+01 | **1.3435E+00** | 5.3670E-01 | 1.0464E+01 | 1.9825E+00 |
| **CGSCA** | 4.0716E+01 | 8.0503E+00 | 6.2623E+00 | 1.2234E+00 | 6.2157E+00 | 6.3193E-01 |
| **HGWO** | 3.4734E+01 | 4.0805E+00 | 3.8859E+00 | 7.3197E-01 | 5.0904E+00 | 6.1707E-01 |
| **AMFOA** | 1.2486E+02 | 3.5166E+00 | 1.6602E+02 | 1.3722E+01 | 1.6001E+01 | 5.2404E-01 |
| **OBSCA** | 4.4758E+01 | 6.5202E+00 | 8.8278E+00 | 4.0827E+00 | 6.3337E+00 | 7.6839E-01 |
| **CIFOA** | 1.1845E+02 | **2.3690E+00** | 1.8111E+02 | 2.6784E+01 | 1.5363E+01 | **5.1960E-01** |
| **DSMFO** | 8.4627E+01 | 3.1951E+01 | 4.3034E+01 | 2.9980E+01 | 8.9516E+00 | 1.8261E+00 |
|  | F7 |  | F8 |  | F9 |  |
|  | AVG | STD | AVG | STD | AVG | STD |
| **CAGWOA** | **8.0310E+02** | 2.5320E+02 | 4.0161E+00 | 3.4693E-01 | 1.3098E+00 | 9.4371E-02 |
| **ASCA_PSO** | 9.5874E+02 | 2.0327E+02 | **3.8522E+00** | 3.3752E-01 | 1.2949E+00 | 9.0227E-02 |
| **CBA** | 1.4917E+03 | 3.1884E+02 | 4.8563E+00 | 1.9336E-01 | 1.4035E+00 | 2.8346E-01 |
| **CGSCA** | 1.2303E+03 | 2.1311E+02 | 4.0758E+00 | 1.9747E-01 | 1.4225E+00 | 7.7937E-02 |
| **HGWO** | 1.2860E+03 | 1.9465E+02 | 4.2594E+00 | 2.7431E-01 | **1.2451E+00** | 6.4907E-02 |
| **AMFOA** | 2.9674E+03 | 2.1013E+02 | 5.5894E+00 | 1.4344E-01 | 7.5800E+00 | **3.3936E-07** |
| **OBSCA** | 1.2734E+03 | 2.3503E+02 | 4.4685E+00 | 1.7308E-01 | 1.4667E+00 | 9.8462E-02 |
| **CIFOA** | 2.5582E+03 | **9.8069E+01** | 5.4234E+00 | **1.2330E-01** | 6.2223E+00 | 7.4238E-01 |
| **DSMFO** | 1.7751E+03 | 2.6145E+02 | 4.5236E+00 | 3.2676E-01 | 1.8732E+00 | 8.1784E-01 |
|  | F10 |  | F11 |  | F12 |  |
|  | AVG | STD | AVG | STD | AVG | STD |
| **CAGWOA** | 2.0986E+01 | **4.1990E-03** | **3.0000E+02** | **4.0963E-13** | 4.0316E+02 | **1.5697E+00** |
| **ASCA_PSO** | 2.0267E+01 | 3.9597E+00 | 3.1960E+02 | 4.3664E+00 | 4.0529E+02 | 2.8151E+00 |
| **CBA** | 2.1035E+01 | 7.7289E-02 | 3.0000E+02 | 1.8800E-07 | **4.0195E+02** | 1.5866E+00 |
| **CGSCA** | 2.0940E+01 | 1.4135E+00 | 2.7728E+03 | 8.0605E+02 | 4.2788E+02 | 9.1048E+00 |
| **HGWO** | 2.0147E+01 | 2.7407E+00 | 1.7508E+04 | 4.0030E+03 | 4.1966E+02 | 9.2982E+00 |
| **AMFOA** | 2.1958E+01 | 3.2376E-01 | 1.6129E+10 | 1.6384E+10 | 9.0593E+02 | 1.4498E+02 |
| **OBSCA** | **1.8944E+01** | 2.8604E+00 | 4.9661E+03 | 2.1138E+03 | 4.3389E+02 | 8.5527E+00 |
| **CIFOA** | 2.1162E+01 | 3.5638E-02 | 1.3885E+07 | 3.2824E+07 | 1.1596E+03 | 1.3840E+01 |
| **DSMFO** | 2.1431E+01 | 8.9180E-02 | 1.8490E+04 | 8.2314E+03 | 6.7463E+02 | 1.7564E+02 |
|  | F13 |  | F14 |  | F15 |  |
|  | AVG | STD | AVG | STD | AVG | STD |
| **CAGWOA** | 6.0561E+02 | 4.4157E+00 | 8.4851E+02 | 1.1203E+01 | 1.1440E+03 | 2.5631E+02 |
| **ASCA_PSO** | **6.0292E+02** | 1.4333E+00 | **8.2115E+02** | 6.3818E+00 | **9.0270E+02** | **6.0198E-01** |
| **CBA** | 6.4695E+02 | 1.3055E+01 | 8.6926E+02 | 2.1806E+01 | 2.7428E+03 | 6.9660E+02 |
| **CGSCA** | 6.1321E+02 | 2.3838E+00 | 8.4519E+02 | 6.3836E+00 | 1.0497E+03 | 5.4910E+01 |
| **HGWO** | 6.0823E+02 | 2.0485E+00 | 8.4029E+02 | 5.5488E+00 | 9.9662E+02 | 3.3774E+01 |
| **AMFOA** | 6.7197E+02 | 3.4264E+00 | 9.3970E+02 | 6.6144E+00 | 4.2093E+03 | 3.7468E+02 |
| **OBSCA** | 6.1366E+02 | 2.7751E+00 | 8.5076E+02 | **5.2822E+00** | 1.0992E+03 | 6.6512E+01 |
| **CIFOA** | 6.5239E+02 | **1.0844E+00** | 9.1621E+02 | 5.7863E+00 | 3.6476E+03 | 3.9440E+02 |
| **DSMFO** | 6.2969E+02 | 1.0052E+01 | 8.7362E+02 | 1.7250E+01 | 1.6934E+03 | 5.8576E+02 |
|  | F16 |  | F17 |  | F18 |  |
|  | AVG | STD | AVG | STD | AVG | STD |
| **CAGWOA** | **6.0034E+03** | **1.8998E+03** | **2.0346E+03** | 1.3909E+01 | 2.2288E+03 | 1.9803E+01 |
| **ASCA_PSO** | 6.3176E+04 | 3.4940E+04 | 2.0354E+03 | **5.8988E+00** | **2.2258E+03** | 5.4562E+00 |
| **CBA** | 1.2817E+04 | 1.1149E+04 | 2.1056E+03 | 3.8160E+01 | 2.2948E+03 | 6.1921E+01 |
| **CGSCA** | 1.0141E+06 | 9.9653E+05 | 2.0548E+03 | 6.3927E+00 | 2.2303E+03 | **2.4197E+00** |
| **HGWO** | 6.5806E+03 | 3.5181E+03 | 2.0485E+03 | 1.2088E+01 | 2.2536E+03 | 4.4554E+01 |
| **AMFOA** | 7.7141E+08 | 1.6008E+08 | 2.3868E+03 | 5.8849E+01 | 2.8108E+03 | 2.4448E+02 |
| **OBSCA** | 1.7162E+06 | 1.2439E+06 | 2.0621E+03 | 8.4245E+00 | 2.2320E+03 | 4.8035E+00 |
| **CIFOA** | 9.1694E+08 | 2.5569E+08 | 2.2834E+03 | 8.0183E+01 | 2.5981E+03 | 2.5733E+02 |
| **DSMFO** | 1.7678E+06 | 2.8281E+06 | 2.0487E+03 | 1.2651E+01 | 2.2666E+03 | 5.0829E+01 |
|  | F19 |  | F20 |  | F21 |  |
|  | AVG | STD | AVG | STD | AVG | STD |
| **CAGWOA** | **2.4000E+03** | **6.1121E-07** | **2.5000E+03** | **1.3860E-07** | **2.6000E+03** | **2.4774E-04** |
| **ASCA_PSO** | 2.5403E+03 | 1.4524E+00 | 2.5503E+03 | 3.7709E+00 | 2.6103E+03 | 9.2577E+00 |
| **CBA** | 2.4000E+03 | 1.8825E-02 | 2.8294E+03 | 5.2933E+02 | 2.6000E+03 | 2.6694E-04 |
| **CGSCA** | 2.4120E+03 | 4.0218E+00 | 2.5037E+03 | 3.3888E+00 | 2.6327E+03 | 2.2566E+00 |
| **HGWO** | 2.4000E+03 | 5.0316E-03 | 2.5024E+03 | 5.0012E-01 | 2.6193E+03 | 8.1977E+00 |
| **AMFOA** | 2.5314E+03 | 2.3778E+00 | 2.5354E+03 | 1.0534E-01 | 2.8672E+03 | 1.5524E+00 |
| **OBSCA** | 2.4207E+03 | 1.1262E+01 | 2.5040E+03 | 1.2810E+00 | 2.6354E+03 | 3.4172E+00 |
| **CIFOA** | 2.4827E+03 | 6.8263E+00 | 2.5229E+03 | 1.1939E+00 | 2.8303E+03 | 2.4015E+01 |
| **DSMFO** | 2.5192E+03 | 1.6651E+01 | 2.5350E+03 | 5.8974E+00 | 2.7004E+03 | 6.8458E+01 |
|  | F22 |  |  |  |  |  |
|  | AVG | STD |  |  |  |  |
| **CAGWOA** | **2.8500E+03** | 7.3957E+01 |  |  |  |  |
| **ASCA_PSO** | 2.9545E+03 | 1.4343E-01 |  |  |  |  |
| **CBA** | 2.9550E+03 | 2.6513E-01 |  |  |  |  |
| **CGSCA** | 2.9491E+03 | 2.4355E+01 |  |  |  |  |
| **HGWO** | 2.9476E+03 | 1.4765E+01 |  |  |  |  |
| **AMFOA** | 2.9841E+03 | 3.3496E+00 |  |  |  |  |
| **OBSCA** | 2.9094E+03 | 3.7945E+01 |  |  |  |  |
| **CIFOA** | 2.9188E+03 | 1.1825E+01 |  |  |  |  |
| **DSMFO** | 2.9550E+03 | **3.1308E-02** |  |  |  |  |

**Table A.7** The results of AVG and STD of CAGWOA and WOA variants

|  | F1 |  | F2 |  | F3 |  |
| --- | --- | --- | --- | --- | --- | --- |
|  | AVG | STD | AVG | STD | AVG | STD |
| **CAGWOA** | **1.5769E+06** | 1.4531E+06 | **1.0107E+04** | **9.4980E+03** | 4.2434E+03 | 1.9653E+03 |
| **OBWOA** | 4.5863E+07 | 2.4633E+07 | 3.6215E+07 | 3.5772E+07 | 2.4971E+04 | 7.4450E+03 |
| **MWOA** | 2.4519E+09 | 9.8170E+08 | 1.0361E+11 | 1.9428E+10 | 9.8614E+05 | 2.0153E+06 |
| **LWOA** | 4.1123E+06 | **1.3161E+06** | 5.0434E+05 | 1.1782E+05 | **1.0125E+03** | **4.0160E+02** |
| **ACWOA** | 1.3195E+08 | 7.3480E+07 | 7.1931E+09 | 3.8711E+09 | 5.0721E+04 | 8.4653E+03 |
| **BWOA** | 7.8858E+07 | 3.7278E+07 | 2.3955E+08 | 1.5940E+08 | 3.5936E+04 | 1.2085E+04 |
| **CCMWOA** | 3.2302E+08 | 1.3229E+08 | 2.9199E+10 | 8.2670E+09 | 6.2015E+04 | 8.0125E+03 |
| **BMWOA** | 1.1445E+08 | 4.4203E+07 | 2.5641E+08 | 1.1534E+08 | 5.3270E+04 | 1.0292E+04 |
| **WOA** | 3.6574E+07 | 1.3880E+07 | 7.2651E+06 | 1.0254E+07 | 3.4771E+04 | 2.2835E+04 |
|  | F4 |  | F5 |  | F6 |  |
|  | AVG | STD | AVG | STD | AVG | STD |
| **CAGWOA** | **4.8993E+02** | **3.1029E+01** | **5.2000E+02** | **0.0000E+00** | 6.3032E+02 | 3.5930E+00 |
| **OBWOA** | 6.3740E+02 | 7.3017E+01 | 5.2067E+02 | 1.1800E-01 | 6.3365E+02 | 3.3680E+00 |
| **MWOA** | 2.2947E+04 | 7.4277E+03 | 5.2130E+02 | 9.1000E-02 | 6.4616E+02 | 2.6380E+00 |
| **LWOA** | 5.0970E+02 | 3.6356E+01 | 5.2048E+02 | 1.1100E-01 | **6.2945E+02** | 4.2000E+00 |
| **ACWOA** | 1.0973E+03 | 2.0676E+02 | 5.2082E+02 | 1.5100E-01 | 6.3437E+02 | 2.5520E+00 |
| **BWOA** | 7.2733E+02 | 1.2263E+02 | 5.2071E+02 | 1.4600E-01 | 6.3583E+02 | 3.0670E+00 |
| **CCMWOA** | 3.0769E+03 | 1.0801E+03 | 5.2088E+02 | 1.4700E-01 | 6.3555E+02 | **2.2470E+00** |
| **BMWOA** | 6.6815E+02 | 5.7409E+01 | 5.2096E+02 | 7.7000E-02 | 6.3202E+02 | 3.2150E+00 |
| **WOA** | 5.8649E+02 | 6.9237E+01 | 5.2032E+02 | 1.8600E-01 | 6.3552E+02 | 3.6560E+00 |
|  | F7 |  | F8 |  | F9 |  |
|  | AVG | STD | AVG | STD | AVG | STD |
| **CAGWOA** | **7.0001E+02** | **1.2000E-02** | 9.0659E+02 | **1.1384E+01** | **1.0832E+03** | **1.3332E+01** |
| **OBWOA** | 7.0133E+02 | 1.7700E-01 | 9.8433E+02 | 2.2836E+01 | 1.1231E+03 | 2.0545E+01 |
| **MWOA** | 1.4583E+03 | 1.9065E+02 | 1.2287E+03 | 3.7693E+01 | 1.4020E+03 | 5.9169E+01 |
| **LWOA** | 7.0070E+02 | 8.4000E-02 | **8.7047E+02** | 1.8154E+01 | 1.1236E+03 | 4.6210E+01 |
| **ACWOA** | 7.3887E+02 | 2.4448E+01 | 9.8043E+02 | 1.9724E+01 | 1.1328E+03 | 2.3726E+01 |
| **BWOA** | 7.0224E+02 | 5.8000E-01 | 9.5800E+02 | 2.0297E+01 | 1.0945E+03 | 1.7819E+01 |
| **CCMWOA** | 9.0024E+02 | 7.6003E+01 | 1.0363E+03 | 2.7800E+01 | 1.1689E+03 | 2.2416E+01 |
| **BMWOA** | 7.0271E+02 | 9.3800E-01 | 9.6415E+02 | 1.8759E+01 | 1.1232E+03 | 3.0283E+01 |
| **WOA** | 7.0102E+02 | 6.1000E-02 | 1.0014E+03 | 5.3753E+01 | 1.1418E+03 | 6.1137E+01 |
|  | F10 |  | F11 |  | F12 |  |
|  | AVG | STD | AVG | STD | AVG | STD |
| **CAGWOA** | 2.4169E+03 | 5.9042E+02 | **4.9459E+03** | 6.4351E+02 | 1.2009E+03 | 3.5900E-01 |
| **OBWOA** | 4.7370E+03 | 6.5961E+02 | 6.0565E+03 | 9.1264E+02 | 1.2017E+03 | 4.6900E-01 |
| **MWOA** | 9.6233E+03 | 5.6273E+02 | 1.0239E+04 | **5.0378E+02** | 1.2054E+03 | 9.3400E-01 |
| **LWOA** | **2.1086E+03** | **4.6568E+02** | 5.2946E+03 | 8.4425E+02 | **1.2008E+03** | **3.4700E-01** |
| **ACWOA** | 4.5655E+03 | 6.0227E+02 | 6.1908E+03 | 8.9923E+02 | 1.2018E+03 | 4.2500E-01 |
| **BWOA** | 5.0925E+03 | 8.1851E+02 | 6.1971E+03 | 1.0998E+03 | 1.2019E+03 | 3.9800E-01 |
| **CCMWOA** | 5.8847E+03 | 4.8023E+02 | 7.1874E+03 | 6.1198E+02 | 1.2020E+03 | 8.3400E-01 |
| **BMWOA** | 4.7027E+03 | 6.9570E+02 | 6.8632E+03 | 7.3593E+02 | 1.2025E+03 | 4.4300E-01 |
| **WOA** | 5.2153E+03 | 7.1253E+02 | 5.9451E+03 | 9.3834E+02 | 1.2017E+03 | 4.1600E-01 |
|  | F13 |  | F14 |  | F15 |  |
|  | AVG | STD | AVG | STD | AVG | STD |
| **CAGWOA** | 1.3005E+03 | 1.2900E-01 | 1.4003E+03 | 1.1800E-01 | 1.5510E+03 | 1.7286E+01 |
| **OBWOA** | **1.3005E+03** | 1.1800E-01 | **1.4003E+03** | 5.5000E-02 | 1.6083E+03 | 4.1797E+01 |
| **MWOA** | 1.3086E+03 | 1.2720E+00 | 1.7123E+03 | 6.5288E+01 | 4.0352E+06 | 3.9030E+06 |
| **LWOA** | 1.3005E+03 | 1.1800E-01 | 1.4003E+03 | **3.9000E-02** | **1.5233E+03** | **6.1050E+00** |
| **ACWOA** | 1.3017E+03 | 9.2400E-01 | 1.4173E+03 | 1.1412E+01 | 2.1301E+03 | 6.3336E+02 |
| **BWOA** | 1.3005E+03 | 1.1400E-01 | 1.4003E+03 | 1.2700E-01 | 1.6174E+03 | 3.5967E+01 |
| **CCMWOA** | 1.3041E+03 | 6.4300E-01 | 1.4663E+03 | 2.2315E+01 | 7.3838E+03 | 4.2522E+03 |
| **BMWOA** | 1.3006E+03 | 1.1700E-01 | 1.4003E+03 | 6.2000E-02 | 1.5878E+03 | 3.6048E+01 |
| **WOA** | 1.3005E+03 | **1.1100E-01** | 1.4003E+03 | 1.4100E-01 | 1.5738E+03 | 2.0917E+01 |
|  | F16 |  | F17 |  | F18 |  |
|  | AVG | STD | AVG | STD | AVG | STD |
| **CAGWOA** | 1.6122E+03 | 5.0700E-01 | **4.4057E+05** | 4.4182E+05 | **3.7079E+03** | **2.9158E+03** |
| **OBWOA** | 1.6123E+03 | 4.4400E-01 | 3.4841E+06 | 2.3054E+06 | 5.0584E+08 | 1.4298E+09 |
| **MWOA** | 1.6141E+03 | **3.2900E-01** | 2.3573E+08 | 1.4016E+08 | 5.8156E+09 | 2.5892E+09 |
| **LWOA** | 1.6124E+03 | 5.1000E-01 | 5.4059E+05 | **2.7245E+05** | 1.5568E+04 | 2.8335E+04 |
| **ACWOA** | **1.6121E+03** | 5.7200E-01 | 1.3144E+07 | 7.7545E+06 | 5.7569E+07 | 7.1236E+07 |
| **BWOA** | 1.6124E+03 | 5.8700E-01 | 8.2021E+06 | 6.1444E+06 | 3.6250E+04 | 7.3046E+04 |
| **CCMWOA** | 1.6131E+03 | 3.7100E-01 | 2.6457E+07 | 1.9692E+07 | 1.4478E+08 | 1.8550E+08 |
| **BMWOA** | 1.6126E+03 | 3.8500E-01 | 6.2255E+06 | 4.4839E+06 | 2.1767E+05 | 7.1034E+05 |
| **WOA** | 1.6125E+03 | 5.6400E-01 | 4.4480E+06 | 2.3187E+06 | 1.6081E+04 | 4.9568E+04 |
|  | F19 |  | F20 |  | F21 |  |
|  | AVG | STD | AVG | STD | AVG | STD |
| **CAGWOA** | **1.9159E+03** | **4.2730E+00** | 4.5818E+03 | 1.9408E+03 | 4.9331E+05 | 3.7621E+05 |
| **OBWOA** | 1.9551E+03 | 3.7569E+01 | 2.7581E+04 | 1.1752E+04 | 3.3880E+06 | 8.2359E+06 |
| **MWOA** | 2.7620E+03 | 3.8237E+02 | 9.3418E+06 | 9.5991E+06 | 1.1330E+08 | 9.2365E+07 |
| **LWOA** | 1.9204E+03 | 2.1004E+01 | **3.2152E+03** | **9.4117E+02** | **2.7011E+05** | **1.9607E+05** |
| **ACWOA** | 2.0096E+03 | 3.0433E+01 | 4.0633E+04 | 1.9158E+04 | 5.5641E+06 | 3.5304E+06 |
| **BWOA** | 1.9590E+03 | 3.5828E+01 | 4.0102E+04 | 2.3608E+04 | 3.0660E+06 | 2.7300E+06 |
| **CCMWOA** | 2.0684E+03 | 4.6974E+01 | 5.1684E+04 | 2.8613E+04 | 6.9805E+06 | 5.6225E+06 |
| **BMWOA** | 1.9391E+03 | 3.2670E+01 | 4.2002E+04 | 3.1380E+04 | 1.7893E+06 | 1.4102E+06 |
| **WOA** | 1.9448E+03 | 3.6445E+01 | 2.4666E+04 | 1.2514E+04 | 1.3327E+06 | 1.3599E+06 |
|  | F22 |  | F23 |  | F24 |  |
|  | AVG | STD | AVG | STD | AVG | STD |
| **CAGWOA** | **2.8461E+03** | **1.7099E+02** | 2.5000E+03 | 0.0000E+00 | 2.6000E+03 | 3.7000E-02 |
| **OBWOA** | 3.4154E+03 | 1.0949E+03 | **2.5000E+03** | 0.0000E+00 | 2.6010E+03 | 9.5400E-01 |
| **MWOA** | 2.4630E+04 | 8.9186E+04 | 3.6621E+03 | 4.0175E+02 | 2.8111E+03 | 4.0558E+01 |
| **LWOA** | 3.0128E+03 | 2.6442E+02 | 2.6154E+03 | 1.2300E-01 | 2.6042E+03 | 4.9000E+00 |
| **ACWOA** | 3.0647E+03 | 2.4293E+02 | 2.5182E+03 | 5.5834E+01 | 2.6000E+03 | 0.0000E+00 |
| **BWOA** | 3.0330E+03 | 2.8187E+02 | 2.5000E+03 | **0.0000E+00** | **2.6000E+03** | **0.0000E+00** |
| **CCMWOA** | 3.3607E+03 | 4.8409E+02 | 2.5000E+03 | 0.0000E+00 | 2.6000E+03 | 0.0000E+00 |
| **BMWOA** | 2.9431E+03 | 2.4572E+02 | 2.5005E+03 | 4.2000E-01 | 2.6003E+03 | 2.7000E-01 |
| **WOA** | 2.9848E+03 | 2.2163E+02 | 2.6282E+03 | 2.5069E+01 | 2.6106E+03 | 2.8301E+01 |
|  | F25 |  | F26 |  | F27 |  |
|  | AVG | STD | AVG | STD | AVG | STD |
| **CAGWOA** | 2.7000E+03 | 0.0000E+00 | 2.7005E+03 | 1.3100E-01 | 2.9000E+03 | 0.0000E+00 |
| **OBWOA** | **2.7000E+03** | 0.0000E+00 | 2.7910E+03 | 2.6360E+01 | 2.9662E+03 | 1.3509E+02 |
| **MWOA** | 2.8382E+03 | 4.4683E+01 | 2.7860E+03 | 1.1207E+02 | 4.4379E+03 | 2.0842E+02 |
| **LWOA** | 2.7171E+03 | 1.1522E+01 | 2.7038E+03 | 1.8215E+01 | 3.6649E+03 | 3.2372E+02 |
| **ACWOA** | 2.7000E+03 | **0.0000E+00** | 2.7603E+03 | 4.9491E+01 | 3.6561E+03 | 3.6839E+02 |
| **BWOA** | 2.7000E+03 | 0.0000E+00 | 2.7502E+03 | 5.0629E+01 | **2.9000E+03** | **0.0000E+00** |
| **CCMWOA** | 2.7000E+03 | 0.0000E+00 | 2.8000E+03 | **0.0000E+00** | 2.9000E+03 | 0.0000E+00 |
| **BMWOA** | 2.7000E+03 | 1.1000E-02 | 2.7006E+03 | 1.3000E-01 | 2.9001E+03 | 2.4100E-01 |
| **WOA** | 2.7153E+03 | 1.4119E+01 | **2.7005E+03** | 1.0400E-01 | 3.7529E+03 | 3.4884E+02 |
|  | F28 |  | F29 |  | F30 |  |
|  | AVG | STD | AVG | STD | AVG | STD |
| **CAGWOA** | 3.0000E+03 | 0.0000E+00 | 3.1000E+03 | 0.0000E+00 | 3.2000E+03 | 5.0000E-03 |
| **OBWOA** | 3.4710E+03 | 9.7280E+02 | 7.0297E+05 | 2.6852E+06 | 2.3703E+04 | 3.8881E+04 |
| **MWOA** | 8.1397E+03 | 8.0491E+02 | 2.5310E+08 | 1.0263E+08 | 5.4881E+06 | 3.0297E+06 |
| **LWOA** | 4.6222E+03 | 4.6083E+02 | 6.6892E+06 | 3.7487E+06 | 1.1863E+04 | 8.1006E+03 |
| **ACWOA** | 3.7688E+03 | 1.1225E+03 | 2.4228E+07 | 1.8791E+07 | 3.9327E+05 | 1.9095E+05 |
| **BWOA** | **3.0000E+03** | **0.0000E+00** | 4.9189E+03 | 7.5900E+03 | 4.0648E+03 | 4.7367E+03 |
| **CCMWOA** | 3.0000E+03 | 0.0000E+00 | **3.1000E+03** | **0.0000E+00** | **3.2000E+03** | **0.0000E+00** |
| **BMWOA** | 3.0001E+03 | 1.4900E-01 | 8.1293E+05 | 1.8224E+06 | 6.2527E+04 | 4.7631E+04 |
| **WOA** | 5.0168E+03 | 5.3725E+02 | 6.0173E+06 | 4.6659E+06 | 7.8059E+04 | 6.2381E+04 |

**Table A.8** Results of SSIM analysis at low threshold levels

| Threshold |  | CAGWOA | WOA | HHO | IWOA | BLPSO | CLPSO | SCADE |
| --- | --- | --- | --- | --- | --- | --- | --- | --- |
| **4** | +/-/= | ~ | 1/0/5 | 1/0/5 | 2/0/4 | 2/0/4 | 1/0/5 | 1/1/4 |
|  | Mean | 1.8333 | 5.0000 | 4.8333 | 4.1667 | 5.1667 | 4.0000 | 3.0000 |
|  | Rank | 1 | 6 | 5 | 4 | 7 | 3 | 2 |
| **6** | +/-/= | ~ | 2/0/4 | 1/0/5 | 2/0/4 | 2/0/4 | 2/0/4 | 3/0/3 |
|  | Mean | 2.6667 | 3.6667 | 3.0000 | 4.6667 | 5.0000 | 4.0000 | 5.0000 |
|  | Rank | 1 | 3 | 2 | 5 | 6 | 4 | 6 |
| **8** | +/-/= | ~ | 2/0/4 | 2/0/4 | 2/0/4 | 2/0/4 | 3/0/3 | 3/0/3 |
|  | Mean | 1.6667 | 4.0000 | 4.3333 | 5.0000 | 3.5000 | 3.1667 | 6.3333 |
|  | Rank | 1 | 4 | 5 | 6 | 3 | 2 | 7 |

**Table A.9** Results of FSIM analysis at low threshold levels

| Threshold |  | CAGWOA | WOA | HHO | IWOA | BLPSO | CLPSO | SCADE |
| --- | --- | --- | --- | --- | --- | --- | --- | --- |
| **4** | +/-/= | ~ | 0/0/6 | 0/0/6 | 0/0/6 | 3/0/3 | 1/0/5 | 1/0/5 |
|  | Mean | 2.1667 | 4.0000 | 2.5000 | 3.8333 | 5.6667 | 4.3333 | 5.5000 |
|  | Rank | 1 | 4 | 2 | 3 | 7 | 5 | 6 |
| **6** | +/-/= | ~ | 1/0/5 | 0/0/6 | 1/0/5 | 3/0/3 | 1/0/5 | 4/0/2 |
|  | Mean | 2.3333 | 2.6667 | 2.6667 | 4.5000 | 5.3333 | 4.5000 | 6.0000 |
|  | Rank | 1 | 2 | 2 | 4 | 6 | 4 | 7 |
| **8** | +/-/= | ~ | 1/0/5 | 4/0/2 | 4/0/2 | 4/0/2 | 5/0/1 | 6/0/0 |
|  | Mean | 1.5000 | 2.0000 | 4.0000 | 3.5000 | 55.0000 | 5.5000 | 6.5000 |
|  | Rank | 1 | 2 | 4 | 3 | 5 | 6 | 7 |

**Table A.10** Results of SSIM analysis at high threshold levels

| Threshold |  | CAGWOA | WOA | HHO | IWOA | BLPSO | CLPSO | SCADE |
| --- | --- | --- | --- | --- | --- | --- | --- | --- |
| **10** | +/-/= | ~ | 4/0/2 | 3/0/3 | 5/0/1 | 2/0/4 | 3/0/3 | 6/0/0 |
|  | Mean | 1.3333 | 3.1667 | 3.6667 | 5.1667 | 2.8333 | 4.8333 | 7.0000 |
|  | Rank | 1 | 3 | 4 | 6 | 2 | 5 | 7 |
| **12** | +/-/= | ~ | 1/0/5 | 2/0/4 | 4/0/2 | 3/0/3 | 4/0/2 | 6/0/0 |
|  | Mean | 1.3333 | 2.0000 | 4.5000 | 5.6667 | 3.3333 | 4.3333 | 6.8333 |
|  | Rank | 1 | 2 | 5 | 6 | 3 | 4 | 7 |
| **14** | +/-/= | ~ | 1/0/5 | 5/0/1 | 5/0/1 | 5/0/1 | 5/0/1 | 6/0/0 |
|  | Mean | 1.1667 | 1.8333 | 5.0000 | 5.3333 | 4.1667 | 4.5000 | 6.0000 |
|  | Rank | 1 | 2 | 5 | 6 | 3 | 4 | 7 |

**Table A.10** Results of FSIM analysis at high threshold levels

| Threshold |  | CAGWOA | WOA | HHO | IWOA | BLPSO | CLPSO | SCADE |
| --- | --- | --- | --- | --- | --- | --- | --- | --- |
| **10** | +/-/= | ~ | 0/0/6 | 4/0/2 | 6/0/0 | 5/0/1 | 5/0/1 | 6/0/0 |
|  | Mean | 1.1667 | 1.8333 | 3.5000 | 5.3333 | 4.5000 | 4.6667 | 7.0000 |
|  | Rank | 1 | 2 | 3 | 6 | 4 | 5 | 7 |
| **12** | +/-/= | ~ | 2/0/4 | 5/0/1 | 6/0/0 | 5/0/1 | 6/0/0 | 6/0/0 |
|  | Mean | 1.3333 | 1.8333 | 3.0000 | 5.3333 | 5.1667 | 4.3333 | 7.0000 |
|  | Rank | 1 | 2 | 3 | 6 | 5 | 4 | 7 |
| **14** | +/-/= | ~ | 1/0/5 | 5/0/1 | 6/0/0 | 6/0/0 | 6/0/0 | 6/0/0 |
|  | Mean | 1.1667 | 1.8333 | 4.1667 | 5.1667 | 4.8333 | 4.3333 | 6.5000 |
|  | Rank | 1 | 2 | 3 | 6 | 5 | 4 | 7 |
